# Supplementary material for: Association between childhood adversities and long-term suicidality among South Africans from the results of the South African Stress and Health study: a cross-sectional study
Source: BMJ Open. 2014 Jun 11;4(6):e004644. doi: 10.1136/bmjopen-2013-004644 (PMC4067829; doi:10.1136/bmjopen-2013-004644)
Supplement: Author's manuscript [file bmjopen-2013-004644.draft_revisions.pdf]

Association between childhood adversities and long-term suicidality among South Africans: Results from the South African Stress and Health Study

Journal:	*BMJ Open*
Manuscript ID:	bmjopen-2013-004644
Article Type:	Research
Date Submitted by the Author:	09-Dec-2013

Complete List of Authors:	Bruwer, Belinda; Stellenbosch University, Psychiatry<br>Govender, Ravi; Stellenbosch University, Psychiatry<br>Bishop, Melanie; Stellenbosch University, Psychiatry<br>Williams, David; Harvard University, Human Development and Health<br>Stein, Dan; University of Cape Town, Psychiatry and mental health<br>Seedat, Soraya; Stellenbosch University, Psychiatry

**Primary Subject Heading**:	Mental health
Secondary Subject Heading:	Mental health
Keywords:	MENTAL HEALTH, Child & adolescent psychiatry < PSYCHIATRY, Adult psychiatry < PSYCHIATRY, PSYCHIATRY, Suicide & self-harm < PSYCHIATRY

SCHOLARONE™
Manuscripts

For peer review only

Association between childhood adversities and long-term suicidality among South Africans: Results from the South African Stress and Health Study

Belinda Bruwer*[a], Ravi Govender[a], Melanie Bishop[a], David R Williams[b] Dan J Stein[c], Soraya Seedat[a]

[a] Department of Psychiatry, Stellenbosch University, PO Box 19063, Tygerberg, 7505, Republic of South Africa.

[b] Department of Society, Human Development and Health, Harvard School of Public Health, and Department of African and African American Studies, Harvard University. Department of Society, Human Development and Health, 677 Huntington Avenue, 6th floor, Boston, MA 02115, United States of America

[c] Department of Psychiatry and Mental Health, University of Cape Town, Groote Schuur Hospital (J2), Anzio Road, Observatory, 7925, Cape Town, Republic of South Africa

*Corresponding author: Department of Psychiatry, University of Stellenbosch, PO Box 19063, Tygerberg, 7505, Republic of South Africa.

Tel nr: +27219404467. Fax nr: +27219404543, bbruwer@sun.ac.za

Keywords: Childhood adversities, suicidal ideation, suicidal attempts, suicide, suicidal behaviour

Word count: 4615

For peer review only

ABSTRACT

Objective:

Suicide and suicidal behaviours are significant public health problems and a leading cause of death worldwide and in South Africa. We examined the association between childhood adversities and suicidal behaviour over the life course.

Methods:

A national probability sample of 4,351 South African adult participants (aged 18 years and older) in the South African Stress and Health (SASH) study was interviewed, as part of the World Mental Health Survey initiative. Respondents provided socio-demographic and diagnostic information, as well as an account of suicide-related thoughts and behaviours. Outcomes were defined as suicide attempts and suicidal ideation in the total sample, and suicide plans and attempts among ideators. Childhood adversities included physical abuse, sexual abuse, parental death, parental divorce, other parental loss, family violence, physical illness and financial adversity. The association between suicidality and childhood adversities was examined using discrete-time survival models.

Results:

More than a third of respondents with suicidal behaviour experienced at least 1 childhood adversity, with physical abuse, parental death and parental divorce the most prevalent adversities. Physical abuse, sexual abuse and parental divorce were identified as significant risk markers for lifetime suicide attempts, while physical abuse and parental divorce were significantly correlated with suicidal ideation. Two or more childhood adversities were associated with a 2-fold higher risk of lifetime suicide attempts. Sexual abuse (OR=9.3, childhood, parental divorce (OR=3.1)

For peer review only - http://bmjopen.bmj.com/site/about/guidelines.xhtml

and physical abuse (OR=2.2) had the strongest associations with lifetime suicide attempts. The effect of childhood adversities on suicidal tendencies varied over the *life course.* For example, sexual abuse was significantly associated with suicide attempts during childhood and teen years, but not during young and later adulthood.

Conclusions:

Childhood adversities, especially sexual abuse, physical abuse and parental divorce are important risk factors for the onset and persistence of suicidal behaviour, with this risk greatest in childhood and adolescence. The risk for suicidal behaviour was greatest in childhood and adolescence. Suicidal risk in childhood and adolescence was significantly associated with the following childhood adversities: sexual abuse, physical abuse and parental divorce.

For peer review only

Strengths and limitations

- These findings extend previous work done in other developing countries that have found childhood adversities to be a significant risk factor for suicidality (20; 57; 58; 59).
- Recall bias might have impacted on the accuracy of recall of childhood adversities.
- Variables such as culture, ethnicity and mental status at the time of the interview might have influenced the recall and reporting of suicidal behaviour.
- Owing to the cross-sectional design, details regarding childhood adversities and suicidal incidents were not assessed. Some of the participants might have been scared to tell the interviewers about their suicidal behaviours. Stigma associated with mental health may have also played a role in reporting suicidal tendencies. The status of the participant's mental health, the role of ethnicity, culture and generational factors may have also contributed to the under-reporting of suicidality.
- The survey was conducted in adults living in households and hostel quarters thus the findings are not generalizable to homeless and institutionalized persons who were not included in the survey.
- The CIDI instrument which was used in this study is a lay-administered instrument which does not include an assessment of several key DSM-IV diagnoses (such as bipolar disorder and psychosis), are associated with elevated rates of suicidality. As a result, some participants with suicidality may have not have been diagnosed with a disorder.

For peer review only - http://bmjopen.bmj.com/site/about/guidelines.xhtml

- In view of the large confidence intervals and small sample sizes for some of these analyses caution is required in drawing conclusions.
- We did not control for other unmeasured causes of childhood adversities and suicidailty, or protective (resiliency) factors that may have contributed to the associations observed in these data.

For peer review only

For peer review only - http://bmjopen.bmj.com/site/about/guidelines.xhtml

INTRODUCTION

Suicide and suicidal behaviour are significant public health problems. Suicide is one of the leading causes of death worldwide with almost 1 million people committing suicide each year (1). This figure is likely to grow to approximately 1.2 million suicides in 2020 (2). In South Africa, the annual rate of suicide is high (3; 4) mirroring international trends (5). So, too, are rates of suicidal behaviour with an estimated prevalence of 9.1% for lifetime suicidal ideation and 2.9% for suicide attempts among South Africans according to the South African Stress and Health Survey (SASH) (6).

Despite the enormity of the problem, the aetiology of suicidal behaviour is not fully understood. There are controversies in the literature regarding prior psychiatric disorder and risk for suicide attempts. While some authors have argued that pre-existing disorder is an important risk factor (7-11), others have argued that suicide attempts are not neccessarily associated with prior psychopathology (12). Genetic factors also play an important role in suicidal behaviour (13-16). While there is stronger evidence pointing towards environmental or experiential factors (17; 18) such as exposure to childhood adversities (19-28). Recent multi-level country data from the World Mental Health Surveys (WMHS) initiative has allowed for cross-national comparisons of suicidality. The WMHS investigated the association between childhood adversities and suicidal behaviour (20), the persistence of suicidality over time, and the extent to which associations between childhood trauma and suicidality changed over the life course. The WMHS found a dose-response relationship between the number of adversities and suicidal behaviour. Sexual abuse and physical abuse were the strongest risk factors for both the onset and persistence of suicidal behaviours, with the risk for suicidality greatest during childhood (age 4-12 years) and adolescence (age 13-19 years) (20).

For peer review only

Numerous studies have examined the link between childhood sexual abuse and suicidality (29-41). All of these authors have found that exposure to childhood sexual abuse increases the risk for mental disorders, including suicidality. Furthermore, the majority of studies that have focused on the link between childhood physical abuse and suicidality have found that exposure to childhood physical abuse increases the risk for suicidality (42; 43). There also appears to be an association between the number of childhood adversities experienced and the later suicidal behaviour (24; 44; 23; 21)

Exposure to early life stress is prevalent among South Africans. In one sample of South African rural youth, the prevalence of physical and sexual abuse was shown to be very high with 94.4% of males exposed to physical abuse and 39.1% of females to sexual abuse (45). More than a quarter of adults who were interviewed endorsed exposure to childhood adversity (parental death, parental separation or parental divorce) in the SASH study (46). Significantly more females were prone to be victims of domestic violence than men (46). Women also reported twice as many suicidal attempts than the male participants in the SASH study (9).

Objective

We report in more detail on data from a South African dataset gathered as part of the World Mental Health Surveys, which allowed for comparison with data from the overall cross-national sample. This data are particularly interesting as South Africa is a middle income African country with high rates of violent trauma exposure. The present study aimed to examine the relationship between the type and frequency of childhood adversity exposure to suicidal behaviour over the life trajectory of South Africans, given that there are no published nationally representative data that may be useful in informing both clinical practice and policy.

For peer review only - http://bmjopen.bmj.com/site/about/guidelines.xhtml

METHODS

Sample

Data for the SASH Study were collected between January 2002 and June 2004. WMH surveys were carried out in 21 countries which included Nigeria and South Africa (46). For detailed information on study methods see Williams et al., 2004 (46). The research protocol for the SASH study was approved by the Human Subjects Committee of the University of Michigan, by Harvard Medical School ethics committee and by a single project assurance of compliance from the Medical University of South Africa (MEDUNSA), and by the National Institute of Mental Health. It was a national probability sample of 4,351 South African adults (persons aged 18 years and older) living in households or in hostel accommodation. All racial and ethnic groups were represented, with the sample selected using a three-stage probability sample design. The response rate was 85.5%.

Sampling approach

Sampling was divided into three stages. Primary sampling units was selected during the first stage, which was based on the 2001 SA census Enumeration Areas (EAs). The second stage involved sampling of household units within clusters selected in each EA. During the third stage, one adult respondent in each sampled housing unit was selected. A total of 5089 households was selected. Field interviews were conducted with 4433 (87.1%) of designated respondents. Based on quality control, 4351 interviews were retained for use in the analysis. There were no differences in response rates across the four designated racial groups.

Diagnostic Interview

SASH used version 3 of the World Health Organization Composite Diagnostic Interview (WHO CIDI) (47). Interviewers were trained within a one week period and conducted the interviews in

For peer review only - http://bmjopen.bmj.com/site/about/guidelines.xhtml

seven different languages, namely English, Afrikaans, Zulu, Xhosa, Northern Sotho, Southern Sotho, and Tswana. Translations of the CIDI into several native South African languages were conducted in accordance with WHO requirements. Multilingual and bilingual expert panels conducted the back-translations (46; 48). Informed consent was obtained from participants after a complete description of the study was provided. Respondents provided socio-demographic and diagnostic information, as well as an account of suicidal behaviours during the interviews. The core diagnostic assessment of mental disorders included anxiety disorders (panic disorder, agoraphobia, social phobia, generalized anxiety disorder, post-traumatic stress disorder), mood disorders (major depressive disorder, dysthymia), substance use disorders (alcohol abuse, alcohol dependence, drug abuse, drug dependence) and intermittent explosive disorder (49, 50). Overall, percentages were weighted to adjust for differences in selection probabilities, differential non-response, oversampling of cases, and residual differences on sociodemographic variables between the sample and the population (46; 51).

Suicidal behaviour

The CIDI 3.0 module on suicidal behaviour was used to assess the age-of-first-onset, age of most recent episode, and lifetime occurrence of suicidal ideation, suicide plans and suicide attempts. Suicidal ideation, suicide plans and suicide attempts was assessed with questions such as "Have you ever seriously **thought** about committing suicide?", "Have you ever made a **plan** for committing suicide?", and "Have you ever **attempted** suicide?", respectively. The outcomes considered in this study were: suicide attempts in the total sample; suicide ideation in the total sample; suicide plans among ideators; suicide attempts among ideators with a plan (planned attempts), and suicide attempts among ideators in the absence of a plan (unplanned or impulsive attempts).

For peer review only - http://bmjopen.bmj.com/site/about/guidelines.xhtml

Childhood adversities

Physical abuse, sexual abuse, parental death, parental divorce, other parental loss, family violence, physical illness and financial adversity were the various childhood adversities assessed. Biological and non-biological parents were included in measures of parental death, divorce or other parental loss. Financial adversities were assessed with questions on whether the family had insufficient funds to pay for basic necessities. Questions about repeated fondling, attempted rape or rape were asked to assess for sexual abuse. A modified version of the Conflict Tactics Scale was used to assess family violence and physical abuse. A standard chronic conditions checklist assessed for life-threatening physical illnesses in childhood (52).

Data analysis

All data analyses were processed and analysed centrally by a team of statisticians at the Harvard School of Public Health (Boston, USA) using the SAS version 9.1.3 software package. Discrete-time survival analysis with time-varying covariates was used to study the risk factors of lifetime suicide ideation, plans and attempts. Data were weighted to adjust for the stratified multistage sample design, differential probability of selection within households as a function of household size and clustering of data, and differential non-response. A post-stratification weight was also used to make the sample distribution comparable, for age, gender, and province, with the population distribution in the 2001 South African census. Both weighted and geographic clustering of data were taken into account in the data analyses by using a jackknife repeated replications simulation method implemented in SAS macro 14. The survival coefficients were exponentiated and are reported below in the form of odds ratios.

The association between suicidality and childhood adversity was examined using discrete-time survival models with the analysis unit being person-years. Bivariate analyses (considering one

For peer review only - http://bmjopen.bmj.com/site/about/guidelines.xhtml

adversity at a time) and multivariate analyses (considering all adversities simultaneously) were conducted. Two types of multivariate models were tested: multivariate additive models (simultaneously considering all childhood adversities) and multivariate interactive models (with number and type of childhood adversities experienced by each respondent included as dummy variables). The analysis also examined interactions between the life stage (13-19 years, 20-29 years, 30+ years) of respondents and each childhood adversity, as well as the influence each adversity had on early-, middle- and later- onset suicidality. Analyses were conducted using SUDAAN version 8.1 to adjust for clustering and weighting. Odds ratios (ORs) with a 95% confidence interval (CIs) are reported. Wald X^2- tests were used to examine multivariate significance. Associations between adversities and suicide outcomes were adjusted for gender, age, educational level, marital status, interactions between demographic variables, life course and parental psychopathology. Analyses also examined the influence of respondents' lifetime mental disorders on suicidality, as well as interactions between gender and each childhood adversity. Statistical significance using two-sided tests was set at p <.05 (20).

RESULTS

Demographic details

In the sample, (n = 4351), there were slightly more female (53.7%) than male respondents. There were more black (76.2%) than coloured (10.4%), white (10%), and Indian/Asian (3.4%) respondents. Furthermore, half of the sample was married and most were unemployed (69.2%), had less than 12 years of education (62.7%) and lived in an urban area (59.7%) (see Table 1).

Prevalence of childhood adversities among the total sample

In the total sample, 35.4% of participants with one adversity had a suicide attempt, compared with 23.4% with one adversity that had not made an attempt. Physical abuse (24.9%), parental

For peer review only - http://bmjopen.bmj.com/site/about/guidelines.xhtml

divorce (14.2%) and parental death (11.6%) were most prevalent among those suicide attempters. Among those exposed to one childhood adversity, without a suicide attempt, the two most prevalent adversities reported were physical abuse (12.2%) and parental death (11.3%). In the total sample 15.4% of participants exposed to two or more adversities had a suicide attempt. In contrast, 8.6% of participants exposed to two or more adversities had not made an attempt (Table 2).

Prevalence of childhood adversities among suicidal ideators in the total sample

In the sample as a whole, 35.9% of those with one adversity had suicidal ideation compared with 22 .7% of those with one adversity who had no ideation. The most prevalent adversities associated with suicidal ideation were physical abuse (21.1%), parental death (13.9%), and parental divorce (7.9%). Among those without suicidal ideation, physical abuse (11.8%) and parental death (11.3%) were the most commonly endorsed childhood adversities. Of those who endorsed two or more childhood adversities, 10.8% reported suicidal ideation and 8.6% did not (Table 2). In summary, the most prevalent childhood adversities reported among the total sample with/without suicidal ideation were firstly, physical abuse and secondly, the death of a parent.

Prevalence of suicide attempts in the total sample

In the total sample, 24.9% of those with childhood physical abuse had attempted suicide while 12.2% of respondents with no physical abuse had no attempt. Of those exposed to parental divorce, 14.2% had attempted suicide and 4.8% had made no attempt. The second most prevalent childhood adversity was parental death with 11.6% of those with parental death attempting suicide and 11.3% of those with parental death with no attempts (Table 2).

For peer review only - http://bmjopen.bmj.com/site/about/guidelines.xhtml

For peer review only

Prevalence of childhood adversities among suicidal ideators

With/without a plan

Among suicidal ideators with a plan, 32.9% had experienced one childhood adversity. Among ideators with no plan, 41.7% had one childhood adversity. Among ideators with a plan, the following were the most prevalent childhood adversities: physical abuse (24.3%), parental death (12.2%), and parental divorce (9.7%). Among ideators without a plan, 27.9% endorsed physical abuse, 16.1% parental death, and 9.2% parental divorce (Table 2). In both groups (ideators with and without a plan), physical abuse was the most prevalent childhood adversity, followed by parental death and parental divorce.

With or without an attempt

Among suicidal ideators who had attempted suicide, 35.4% were exposed to one childhood adversity and 15.4% were exposed to two or more childhood adversities. In the group of ideators who had made an attempt, 24.9% had experienced physical abuse, 14.2% parental divorce, and 11.6% parental death (Table 2). 40.5% of those with one adversity, and 9.6% of those exposed to two or more adversities were suicidal ideators with no attempts. In this group, the most prevalent adversities were physical abuse (24.5%); parental death (15.6%) and parental divorce (6.7%) reported (Table 2).

Among all ideators (with/without a plan, with/without an attempt), the most prevalent childhood adversity was physical abuse, followed by parental death and parental divorce. Of note, in the group of ideators with an attempted suicide parental divorce was more prevalent than parental death.

Bivariate and multivariate results: Type of childhood adversity

For peer review only - http://bmjopen.bmj.com/site/about/guidelines.xhtml

Bivariate and multivariate analyses were performed to examine the associations between the different childhood adversities (physical abuse, sexual abuse, parental death, parental divorce, other parental loss, family violence, physical illness, financial adversity) and lifetime suicidal ideation, plans and attempts.

In the total sample, bivariate analysis revealed significant associations between (i) sexual abuse (OR=7.9, p=0.003), (ii) physical abuse (OR 2, p=0.007) and (iii) parental divorce (OR 2.8, p<.001), and lifetime suicide attempts. Among ideators in the sample, physical abuse (OR=1.7, p<.001) was significantly associated with suicidal ideation. The relationship between childhood adversities and lifetime plans was not statistically significant. However, a significant association was found between parental divorce and lifetime suicidal attempts among ideators (OR=3.0, p<.001) (See Table 7).

Multivariate analysis also revealed a significant association between (i) sexual abuse (OR=7.6, p=0.003), (ii) physical abuse (OR 2.0, p=0.006) and (iii) parental divorce (OR 2.7, p=0.001) and lifetime suicide attempts, in the total sample. Physical abuse (OR=1.7, p<.001) and parental divorce (OR = 1.6, p=0.038) were both significantly associated with suicidal ideation in the overall sample. Among ideators, no significant associations were found between any of the childhood adversities and lifetime plans. However, the relationship between parental divorce and lifetime suicidal attempts among ideators was significant (OR=3.1, p=0.023) (Table 3).

Findings from multivariate analysis, therefore, confirm findings of bivariate analysis for all groups, except for ideators. Among ideators bivariate analysis revealed a significant relationship between physical abuse and suicidal ideation. This was confirmed in multivariate analysis where

For peer review only - http://bmjopen.bmj.com/site/about/guidelines.xhtml

the association between parental divorce and suicidal ideation was significant for the whole sample.

Bivariate associations between the number of adversities and lifetime suicidality

The relationship between the number of childhood adversities and lifetime suicidal ideation, plans and attempts was further examined. There was a significant relationship between the number of childhood adversities and lifetime suicide attempts. Two or more childhood adversities were associated with a 2-fold higher risk of lifetime suicide attempts in the total sample (OR=2.1, $p<.001$). A significant relationship was also established between one, as well as two or more adversities with ideators in the total sample. Among ideators, no significant association was found between the number of childhood adversities and lifetime plans. A significant relationship was found between two or more adversities and lifetime attempts among ideators (OR=2.7, $p=0.016$), indicating a more than 2-fold higher risk of lifetime suicide attempts in this group (Table 4).

Multivariate associations between number of childhood adversities and lifetime suicidality

In the final multivariate model which included 2 or more adversities as a predictor variable, sexual abuse (OR=9.3, $p<.001$), childhood physical abuse (OR=2.2, $p=0.003$) and parental divorce (OR=3.1, $p<.001$) retained significant associations with lifetime suicide attempts in the total sample. Physical abuse (OR=2.1, $p<.001$), parental death (OR=1.7, $p=0.010$), parental divorce (OR=1.9, $p=0.004$) and other parental loss (OR = 2.1, $p=0.004$) were significant predictors of suicidal ideation. Physical abuse (OR=0.4, $p=0.038$) was significantly associated with lifetime plans among ideators. There were no significant associations between childhood adversities and lifetime attempts among those with suicidal ideation (Table 5).

For peer review only - http://bmjopen.bmj.com/site/about/guidelines.xhtml

Associations between the types of childhood adversity and lifetime suicidality over the life course

Multivariate analyses were performed to examine the association between the types of childhood adversity and lifetime suicidal ideation, plans and attempts during childhood years (age 4- 12), teenage years (age 13-19), young adulthood (age 20-29) and later adulthood (30 years and older) (See tables 6, 6a, 6b, 6c and 6d).

Childhood years (4-12). Sexual abuse (OR=61.6, p=0.002) in early childhood (4-12 years of age) was significantly associated with lifetime suicide attempts in the total sample (OR = 61.6, CI=4.5-841.0, p=0.002). Both sexual abuse (OR=34.8, CI= 3.1-392.6, p=0.003) and physical abuse (OR=3.7, p=0.041) were associated with a higher risk for suicidal ideation among the total sample. No significant associations were found between any of the childhood adversities and lifetime plans in the group of ideators. Among those with suicidal ideation, parental death (OR=22.7, p=0.021) was significantly associated with suicide attempts in childhood years (See Table 6a).

Teen years (13-19). Sexual abuse (OR=20.3, p=0.010), physical abuse (OR=3.7, p=0.004), and parental divorce (OR=4.6, p=0.002) were significantly associated with suicide attempts in the total sample of teenagers. Physical abuse (OR=3.6, p<.001) and parental death (OR=2.2, p=0.021) significantly increased the risk for suicidal ideation among the total group of teens. Physical illness (OR=9.9, p=0.007) significantly increased the risk of suicidal plans in teens with suicidal ideation. Suicide attempts among teens with suicidal ideation was significantly predicted by parental divorce (OR=4.3, p=0.035) (See Table 6b).

For peer review only - http://bmjopen.bmj.com/site/about/guidelines.xhtml

Young adulthood (20-29). None of the childhood adversities were significantly associated with lifetime suicide attempts during young adulthood in the sample overall. An explanation could be that suicide attempts spike earlier and later in life among South Africans, contributing to the lack of significance. Parental loss other than parental death was significantly associated with suicidal ideation (OR=2.9, p=0.019) (See Table 6c).

Later adulthood (≥ 30). Childhood physical abuse (OR=2.2, p=0.035) was significantly predictive of suicidal attempts. The likelihood of suicidal ideation significantly increased in later adulthood if parental loss other than parental death (OR=5.1, p<.001) or physical illness had been present during childhood (OR=4.3, p=0.028). No significant relationship was found between any of the childhood adversities and lifetime plans in the group of ideators although a significant relationship was found between two or more adversities and lifetime plans among those who were ideators (OR=44.5, p<0.008). None of the childhood adversities were significantly associated with suicide attempts among ideators in this age group (See Table 6d).

DISCUSSION

Rates of childhood adversities and suicidal behaviours were both high among South Africans, with more than a third of respondents in the total sample who attempted suicide experiencing one childhood adversity, and 15.4% experiencing two or more adversities. Overall, physical abuse, sexual abuse, parental divorce and physical illness were far more prevalent in those with a suicide attempt than in those without. The most prevalent childhood adversities endorsed overall were physical abuse followed by parental death. Physical abuse, parental divorce and death of a parent were also the most prevalent adversities experienced in those with a suicide attempt as well as in those with suicidal ideation. These findings are somewhat dissimilar to other country samples; for example in the 21 countries that participated in the WMHS, physical abuse (29.3%),

For peer review only - http://bmjopen.bmj.com/site/about/guidelines.xhtml

family violence (24.8%) and neglect (19.3%) were the most prevalent childhood adversities among those with a lifetime suicide attempt, while physical abuse (20.6%), family violence (17.6%) and death of a parent (14.2%) were most often reported among participants with lifetime suicidal ideation (20). Cross-nationally, it would appear that physical abuse is the commonest childhood adversity associated with lifetime suicide attempts and ideation (20).

The estimate lifetime prevalence of 2.9% for attempted suicide among South Africans is close to the rates of 4.6% and 4.1% reported for general and Black populations respectively in USA. In addition the 9.1% estimated prevalence of suicide ideation is comparable with previous estimates from studies in South African clinical samples. Joe et al. (2008) reported for the first time on the rates of suicide ideation, plan and attempts among the different ethnic groups, in data from the SASH study (9). Overall, the results suggest that people in SA engage in suicidal thought and behaviours at levels nearly comparable with those of Western nations.

When examining suicidal behaviour risk in the context of childhood adversity, sexual abuse, physical abuse and parental divorce emerged as significant risk factors for lifetime suicide attempts in the total sample. Furthermore, physical abuse and parental divorce were significant risk factors for suicidal ideation in the total sample, while parental divorce emerged as a significant risk factor among ideators with lifetime suicide. These findings are largely consistent with the data from the overall cross-national WMHS, which found that physical and sexual abuse significantly increased the likelihood of suicidal ideation and attempts, while neglect was a risk factor for suicidal behaviour in multivariate additive analyses (20).

Of the adversities implicated, sexual and physical abuse were more significant risk factors than other adversities, highlighting the fact that intrusive and aggressive experiences in childhood

For peer review only - http://bmjopen.bmj.com/site/about/guidelines.xhtml

may have more devastating and longer lasting effects (53). This may be due to the extreme powerlessness and loss of control that such abuse causes, or to physically aggressive assaults resulting in the devaluation of one's body and consequent susceptibility to self harm (28). In a country with high rates of sexual and physical abuse (45) this is particularly concerning. The impact of parental divorce on suicidality supports previous findings that parental divorce, if accompanied by other adversities such as childhood abuse, increases the risk of suicidal behaviour (54).

We also found that exposure to *two or more childhood adversities* significantly increased the risk of suicide attempts among ideators. This confirms earlier work showing exposure to multiple childhood adversities increases the risk of suicidal behaviour (21; 23; 24; 55; 56). Bruffaerts et al (2010) found a sub-additive effect with regards to the onset of suicidal behaviour when considering multiple adversities (20). Thus, the impact of multiple adversities was not equal to the sum of the odds ratios of individual adversities. In the overall WMHS analysis exposure to multiple childhood adversities had a significant effect on the persistence of suicide when considering every additional childhood adversity exposed to, however in the current study it was not possible to stratify the number of adversities beyond two or more adversities (i.e. into more than 2 categories) given the relatively small number of cases in the sample overall with non-fatal suicidal behaviour. Physical abuse, parental death, parental loss other than through death, and parental divorce emerged as independent risk factors for suicidal ideation in the total sample. Moreover, the effects of childhood adversities on suicidal tendencies tended to differ over the *life course*. Consistent with nationally representative data in WMHS, childhood adversities were associated with the highest risk of suicide attempts in childhood, with a decrease in risk in

For peer review only - http://bmjopen.bmj.com/site/about/guidelines.xhtml

adolescence and young adulthood, followed by an increase in risk again during later adulthood (20).

In *childhood*, sexual abuse was significantly associated with lifetime suicide attempts in the total sample, while sexual and physical abuse was significantly associated with suicidal ideation. Among suicidal ideators, parental death was significantly associated with lifetime suicide attempts. Exposure to childhood sexual abuse, physical abuse or parental divorce significantly increased suicide attempts during *teenage years*, while physical abuse and parental death were associated with suicidal ideation in teens. Among teen suicidal ideators, physical illness was significantly associated with suicidal plans, while parental divorce was associated with suicide attempts. These findings emphasize the need to focus suicide prevention strategies at youth in particular. In *young adulthood,* parental loss other than the death of a parent was significantly associated with suicidal ideation in the total sample. Interestingly, childhood physical abuse was identified as a significant risk factor for suicidal attempts in *later adulthood*, while childhood physical illness and parental loss other than the death of a parent significantly increased the risk for ideation.

Similar to findings from SASH, childhood sexual abuse emerged as a particularly robust risk factor for suicide attempts in younger participants in the WMH cross-national analysis, with a 10.9 times higher odds of suicide attempts in children, a 6.1 times higher likelihood in adolescents and a 2.9-fold risk in young adults who were exposed (20). This is in keeping with Enns hypothesis that sexual abuse results in suicidal behaviour at a younger age (21). Consistent with other studies, childhood physical and sexual abuse, in particular, emerged as risk factors for the emergence and persistence of suicidal behaviour, especially in adolescence. Loss of a parent, physical ill-health and family violence has also been found to be associated with persistence of

For peer review only - http://bmjopen.bmj.com/site/about/guidelines.xhtml

For peer review only

suicidality (20; 28; 53). These findings extend previous work done in other developing countries that have found childhood adversities to be a significant risk factor for suicidality (20; 57; 58; 59).

Limitations

The following limitations need to be highlighted. First, recall bias might have impacted on the accuracy of recall of childhood adversities. This said, participants were asked questions about childhood adversities in sequence which may have facilitated more accurate recall (60). Systematic reviews have also found that recall of past experiences can be accurate and can provide valuable data (61; 62). Thus, there is evidence to support the validity of accurate recall of childhood adversities (62). Further, studies have shown that responses to questions on childhood adversities, similar to those asked in the SASH study, generally remain stable over time (63; 64). Second, variables such as culture, ethnicity and mental status at the time of the interview might have influenced the recall and reporting of suicidal behaviour. Third, owing to the cross-sectional design, details regarding childhood adversities and suicidal incidents were not assessed. However, it is much more likely that adversities and suicidality were under-reported rather than over-reported (6; 20; 62; 65). Some of the participants might have been scared to tell the interviewers about their suicidal behaviours. Stigma associated with mental health may have also played a role in reporting suicidal tendencies. The status of the participant's mental health, the role of ethnicity, culture and generational factors may have also contributed to the under-reporting of suicidality. Fourth, the survey was conducted in adults living in households and hostel quarters thus the findings are not generalizable to homeless and institutionalized persons who were not included in the survey. Fifth, the CIDI instrument which was used in this study is a lay-administered instrument which does not include an assessment of several key DSM-IV

For peer review only - http://bmjopen.bmj.com/site/about/guidelines.xhtml

diagnoses (such as bipolar disorder and psychosis), are associated with elevated rates of suicidality. As a result, some participants with suicidality may have not have been diagnosed with a disorder. Furthermore, in view of the large confidence intervals and small sample sizes for some of these analyses caution is required in drawing conclusions. Lastly, we did not control for other unmeasured causes of childhood adversities and suicidailty, or protective (resiliency) factors that may have contributed to the associations observed in these data. Both other risk and resiliency factors may have contributed to both the prevalence of non-fatal suicidal behaviours and to the associations with different forms of childhood adversity and warrant further investigation.Notwithstanding these limitations, this study represents the first investigation among South Africans of a wide range of childhood adversities and their impact on the onset and persistence of suicidality over the life course.

Conclusions

Childhood adversities especially sexual abuse, physical abuse and parental divorce are important risk factors for the onset and persistence of suicidal behaviour with the risk greatest in children and adolescents. Public health efforts aimed at prevention of early childhood sexual and physical abuse, in particular, may have a significant impact on reducing suicidality over the life course and improving mental health outcomes.

For peer review only

Acknowledgements

The South African Stress and Health Study were conducted in conjunction with the World Health Organization World Mental Health (WMH) Survey initiative. We thank the WMH staff for assistance with instrumentation, fieldwork and data analysis. The US National Institute of Mental Health, the John D. and Catherine T. MacArthur Foundation, the Pfizer Foundation, US Public Health Service, the Fogarty International Center, Pan American Health Organization, Eli Lilly and Company, Ortho-McNeil Pharmaceutical, GlaxoSmithKline and Bristol-Myers Squibb supported the activities of this initiative. The SASH Study was funded by grant R01-MH059575 from the National Institute of Mental Health and the National Institute of Drug Abuse.

Declarations of interest and funding

This study was funded by NIH. Additional funding was received from the South African Department of Health and the University of Michigan. D.J.S. and S.S. are also supported by the Medical Research Council of South Africa. D.J.S. has received research grants and / or consultancy honoraria from AstraZeneca, Eli Lilly, GlaxoSmithKline, Lundbeck, Orion, Pfizer, Pharmacia, Roche, Servier, Solvay, Sumitomo and Wyeth. S.S. has received research grants or travel sponsorship from AstraZeneca, Eli Lilly, GlaxoSmithKline, Lundbeck and Servier. S.S. is supported by the by the South African Research Chairs Initiative of the Department of Science and Technology and the National Research Foundation. B.B has received congress sponsorship from Janssen-Cilag.

For peer review only

For peer review only - http://bmjopen.bmj.com/site/about/guidelines.xhtml

REFERENCES

1. World Health Organization. Suicide Prevention (SUPRE). Geneva, Switzerland. 2007. http://www.who.int/mental_helath/prevention/suicide/suicideprevention/en/)

2. Murray, C.L., Lopez, A.D. The global burden of disease: a comprehensive assessment of mortality and disability from disease, injuries and risk factors in 1990 and projected to 2020. Cambridge, MA: Harvard University Press, 1996.

3. Burrows, S., Laflamme, L. Pattern analysis of suicide mortality surveillance data in urban South Africa. Suicide and Life- Threatening Behaviour 2008;**38**:209-220.

4. Meel, B.I. Epidemiology of suicide by hanging in Transkei. South Africa. Am J Forensic Med Pathol. 2006;**27**:75-78.

5. Flisher, A.J., Liang, H., Laubscher, R. Suicide trends in South Africa, 1968-90. Scand J Public Health 2004;**32**:411-418.

6. Joe, S., Stein, DJ., Seedat, S., Herman, A., Williams, DR. non-fatal suicidal behavior among South Africans: Results from the South Africa Stress and Health Study. Social Psychiatry Epidemiology 2008;**43**(6):454–461.doi:10.1007/s00127-008-0348-7.

7. Beautrais, A.L., Joyce, P/R/. & Mulder, R.T. (1996). Risk factors for serious suicide attempts among youth aged 13 through 24 years. J Am Acad Child Adolesc Psychiatry 1996;**35**(9):1174-1182.

8. Harrison, EC, Barraclough, B. (1997). Suicide as an outcome for mental disorders: A meta-analysis. Br J Psychiatry 1997;**170**:205-228

For peer review only - http://bmjopen.bmj.com/site/about/guidelines.xhtml

9. Joe, S., Stein, D.J., Seedat, S., et al. Prevalence and correlates of non-fatal suicidal behaviour among South Africans. Br J Psychiatry 2008;**192**:310-311.

10. Nock, M.K., Borges, G., Bromet, E.J., et al. Suicide and Suicidal Behaviour. Epidemiologic Reviews 2008;**30**:133-154.

11. Nock, M.K., Borges, G., Bromet, E.J., et al. Cross-national prevalence and risk factors for suicidal ideation, plans and attempts. Br J Psychiatry 2008;**192**:98-105.

12. Nock, M.K., Hwang, I., Sampson, N.A., et al. Cross-national analysis of the associations among mental disorders and suicidal behaviour: Findings from the WHO World Mental Health Surveys. PLos Medicine 2009;**6**(8).e1000123.

13. Bondy, B., Buettner, A., Zill, P. Genetics of suicide. Molecular Psychiatry 2006;**11**:336-351.

14. Kohli, M.A., Salyakina, D., Pfennig, A., et al. Association of genetic variants in the neurotrophic receptor encoding gene NTRK2 and a lifetime history of suicide attempts in depressed patients. Arch Gen Psychiatry 2010;**67**:348-59.

15. Roy, A., Hu, X-Z., Janal, M.N., & Goldman, D. Interaction between childhood trauma and serotonin transporter gene variation and suicide. Neuropsychopharmacology 2007;**32**:2046–2052

16. Risch, N., Herrell, R., Lehner, T., et al. Interaction between the serotonin transporter gene (5-HTTLPR), stressful life events, and the risk of depression: A meta-analysis. JAMA 2009;**301**:2462–2471.

For peer review only

17. Borges, G., Benjet, C., Medina-Mora, M.E., et al. Traumatic events and suicide related outcomes among Mexico City adolescents. J Child Psychol Psychiatry 2008;**6**:654-666.

18. Weissman MM, Bland RC, Canino GJ, Greenwald S, Hwu HG, Joyce PR, et al. Prevalence of suicide ideation and suicide attempts in nine countries. Psychology Med 1999;**29**:9–17.

19. Brodsky, BS & Stanley, B. Adverse childhood experiences and suicidal behaviour. Psychiatry Clinical Northern America 2008;**31**:223-235

20. Bruffaerts, R., Demyttenaere, K., Borges, G., et al. Childhood adversities as risk factors for onset and persistence of suicidal beahviour. Br J Psychiatry 2010;**197**:20-27.

21. Enns, M.W., Cox, B.J., Afifi, T.O., et al. Childhood adversities and risk for suicidal ideation and attempts: a longitudinal population-based study. Psychological Medicine 2006;**36**:1769-1778.

22. Johnson, J.G., Cohen, P., Gould, M.S., et al. Childhood adversities, interpersonal difficulties, and risk for suicide attempts during late adolescence and early adulthood. Arch Gen Psychiatry 2002;**59**:741-749.

23. Dube, S.R., Anda, R.F., Felitti, V.J., et al. Childhood abuse, household dysfunction, and the risk of attempted suicide throughout the life span: Findings from the Adverse Childhood Experiences Study. JAMA 2001;**286**:3089-3096.

24. Afifi, T.O., Enns, M.W., Cox, B.J., et al. Population attributable fractions of psychiatric disorders and suicide ideation and attempts associated with adverse childhood experiences. Am J Public Health 2008;**98**:946-952.

For peer review only - http://bmjopen.bmj.com/site/about/guidelines.xhtml

25. Burke, A.K., Galfalvy, H., Everett, B., et al. Effect of exposure to suicidal behavior on suicide attempt in a high-risk sample of offspring of depressed parents. J Am Acad Child Adolesc Psychiatry 2010;**49**:114-121.

26. Labonte, B., Suderman, M., Maussion, G., Navaro, L., Yerko, V., Mahar, I., & Turecki, G. Genome-wide epigenetic regulation by early-life trauma. Arch Gen Psychiatry 2012;**69**(7):722-731.Doi:10.1001/archgenpsychiatry.2011.2287

27. Lipschitz, D.S., Winegar, R.K., Nicolaou, A.L., et al. Perceived abuse and neglect as risk factors for suicidal behaviour in adolescent inpatients. J Nerv Ment Dis 1999;**187**:32-39.

28. Ystgaard, M., Hestetun, I., Loeb, M., & Mehlum, L. Is there a specific relationship between childhood sexual and physical abuse and repeated suicidal behaviour? Child Abuse Neg 2004;**28**:863-875.

29. Boudewyn, A., & Liem, J. Childhood sexual abuse as a presecutor to depression and self-destructive behavior in adulthood. J Trauma Stress 1995;**8**:445-459.

30. Brown, J., Cohen, P., Johnson, J.G., & Smailes, E.M. Childhood abuse and neglect: Specificity of effects on adolescent and young adult depression and suicidality. J Am Acad Child Adolesc Psychiatry 1999;**38**:1490-1496.

31. Bryant, S.L., & Range, L.M. Suicidality in college women who were sexually and physically abused and physically punished by parents. Violence Vict 1995;**10**:195-201.

32. Davidson, J.R.T., Hughes, D.C., George, L.K., & Blazer, D.G. The association of sexual assault and attempted suicide within the community. Arch Gen Psychiatry 1996;**53**:550-555

For peer review only

33. Fergusson, D.M., & Mullen, P.E. Childhood Sexual abuse – An evidence based perspective. Sage, CA: Thousand Oaks, 1999.

34. Finkelhor, D. Early and long-term effects of child sexual abuse: An update. Professional Psychology: Research & Practice 1990;**21**(5):325-330.

35. Finkelhor, D., & Hashima, P.Y. (2001). The victimization of children and youth: A comprehensive overview. In S.O. White (Ed.) Handbook of youth and justice. The Plenum series in crime and justice. Dordrecht: Plenum, 2001:49-78.

36. Holmes, W.C., & Slap, G.B. Sexual abuse of boys: Definition, prevalence, correlates, sequelae, and management. JAMA: JAMA 1998;**280**(21):1855-1862

37. Kendall-Tackett, K.A., Williams, L.M., & Finkelhor, D. Impact of sexual abuse on children: A review and synthesis of recent empirical studies. Psychol Bull 1993;**113**(1):164-180.

38. Martin, G. Reported family dynamics, sexual abuse, and suicidal behaviors in community adolescents. Arch Suicide Res 1996;**2**:183-195.

39. Peters, D.K., & Range, L.M. Childhood sexual abuse and current suicidality in college women and men. Child Abuse Negl 1995;**19**:335-341.

40. Putman, F.W. Ten-year research update review: Child sexual abuse. J Am Acad Child Adolesc Psychiatry 2003;**42**(3):269-278

41. Stepakoff, S. Effects of sexual victimization on suicidal ideation and behaviour in US college women. Suicide and Life-Threatening Behavior 1998;**28**:107-126.

For peer review only - http://bmjopen.bmj.com/site/about/guidelines.xhtml

For peer review only

42. Malinosky-Rummel, R., & Hansen, D.J. Long-term consequences of childhood physical abuse. Psychol Bull 1993;**144**:68-79

43. Silverman, A.B., Reinherz, H., & Giaconia, R.M. The long-term sequelae of child and adolescent abuse: A longitudinal community study. Child Abuse Negl 1996;**20**:709-723

44. Chapman, D.P., Whitfield, C.L., Felitti, V.J., Dube, S.R., Edwards, V.J., & Anda, R.F. Adverse childhood experiences and the risk of depression in adulthood. J Affect Disord 2004;**82**:217-225

45. Jewkes, R.K., Dunkle, K., Nduna, M., et al. Associations between childhood adversity and depression, substance abuse and HIV and HSV2 incident infections in rural South African youth. Child Abuse Negl 2010;**34**:833-841.

46. Williams, D.R., Herman, A., Kessler, R.C., et al. The South Africa Stress and Health Study: Rationale and Design. Metab Brain Dis 2004;**19**(1/2):135-147.

47. Kessler, R.C., Üstün, T.B. The World Mental Health (WMH) Survey Initiative Version of the World Health Organization (WHO) Composite International Diagnostic Interview (CIDI). Int J Methods Psychiatr Res 2004;**13**:61-98.

48. Seedat, S., Stein, D.J., Herman, A., et al. Twelve-month treatment of Psychiatric disorders in South Africa Stress and Health Study (World Mental Health Survey Initiative). Psychiatric Epidemiology 2008;**38**:211-220.

For peer review only

49. World Health Organization. World Health Organization Manual of the international statistical classification of diseases, injuries and causes of death, ninth revision. Geneva, Switzerland, 1992.

50. American Psychiatric Association. Diagnostic and statistical manual of mental disorders (DSM-IV), 4th Edition. Washington: American Psychiatric Association Press, 1994.

51. Stein, D.J., Chiu, W.T., Hwang, I., et al. Cross-national analysis of the associations between traumatic events and suicidal behavior: Findings from the WHO World Mental Health Surveys. PloS ONE 2010;**5**(5):e10574.

52. Kessler, R.C., McLaughlin, K.A., Green, J.G. Childhood adversities and adult psychopathology in the WHO World Mental Health Surveys. Br J Psychiatry 2010;**197**:378-385.

53. Joiner Jr, T.E., Sachs-Ericsson, N.J., Wingate, L.R. Childhood physical and sexual abuse and lifetime number of suicide attempts: A persistent and theoretically important relationship. Behav Res Ther 2007;**45**:539-547.

54. Afifi, T.O., Boman, J., Fleisher, W., et al. The relationship between child abuse, parental divorce, and lifetime mental disorders and suicidality in a nationally representative adult sample. Child Abuse Negl 2009;**33**:139–147.

55. Bebbington, P.E., Cooper, C.C., Minot, S., et al. Suicide attempts, gender, and sexual abuse: data from the 2000 British Psychiatric Morbidity Survey. Am J Psychiatry 2009;**166**:1135-1140.

For peer review only

For peer review only - http://bmjopen.bmj.com/site/about/guidelines.xhtml

56. Molner, B, Buka, S, & Kessler, R. Child sexual abuse and subsequent psychopathology: results from the National Comorbidity Survey. American Journal Public Health 2001;**91**:753-760.

57. Borges, G., Angst, J., Nock, M.K., et al. Risk factors for the incidence and persistence of suicide related outcomes: a 10 year follow up study using the National Comorbidity Surveys. J Affect Disord 2008;**105**:25-33

58. Gureje, O., Kola, L., Uwakwe, R., et al. The profile and risks of suicidal behaviours in the Nigerian Survey of Mental Health and Well Being. Psychol Med 2007;**37**:821-830.

59. Xing, X-Y., Tao, F-B., Wan, Y-H., et al. Family factors associated with suicide attempts among Chinese adolescent students: A national cross-sectional survey. J Adolesc Health 2010;**46**:592-599.

60. Knauper, BC., CF, Schwarz, N., Bruce, ML., Keesler, RC. Improving the accuracy of major depression age of onset reports in the US National Comorbidity Survey. Int J Methods Psychiatr Res 1999;**8**(1):39-48

61. Brewin, CR., Andrews, B., Botlib, IH. Psychopathology and early experience: a reappraisal of retrospective reports. Psychol Bull 1993;**113**:82-98.

62. Hardt, J., Rutter, M. Validity of adult retrospective reports of adverse childhood experiences: a review of the evidence. J Child Psychol Psychiatry 2004;**45**:260-273.

For peer review only

63. Dube, SR., Williamson, DF., Thompson, T., Felitti, VJ, Anda, RF. Assessing the reliability of retrospective reports of adverse childhood experiences amond adult HMO members attending a primary care clinic. Child Abuse Negl 2004;**28**(7):729-737.

64. Yancura, LA., Aldwin, CM. (2009). Stability and change in retrospective reports of childhood experiences over a 5-year period: Findings from the David Longitudinal Study. Psychol Aging 2009;**24**(3):715-721

65. Wilsnack, S.C., Wonderlich, S.A., Kristjanson, A.F., et al. (2002). Self reports of forgetting and remembering childhood sexual abuse in a nationally representative sample of US women. Child Abuse Negl 2002;**26**:139-147.

For peer review only

For peer review only - http://bmjopen.bmj.com/site/about/guidelines.xhtml

Table 1: Descriptive Characteristics (N= 4351)

Mean Age (yrs) (SE)	37.0 (0.26)
**Age categories (yrs)**	
18 – 29	39.1%
30 – 39	22.1%
40 - 49	18.1%
≥ 50	20.7%
**Gender**	
Male	46.3%
Female	53.7%
**Race**	
Black	76.2%
Coloured	10.4%
White	10.0%
Indian/Asian	3.4%
**Married**	50.1%
**Location**	
Rural	38.4%
Urban	61.6%
**Education**	
None	6.8 %
Grade 1-7	19.1%
Grade 8-11	35.4%
Matric	23.5%
Matric +	15.3%
**Employed**	31.0%
**Income Category** (Rands), (mean SD)	
0	13.7%
1 - 2500	29.5%
2501 – 5000	15.4%
5001 – 10 000	19.6%
≥ 10001	21.8%
**Province**	
Eastern Cape	13.1%
Free State	6.2%
Guateng	23.0%
Kwazulu Natal	19.5%
Limpopo	10.5%
Mpumalanga	6.6%
Northern Cape	1.9%
North West	8.3%
Western Cape	11.1%

For peer review only

Table 2: Prevalence of childhood adversities and suicidal behaviour in South Africa

[% (S.E.)]

	Total Sample		Total Sample		Suicidal Ideators		Suicidal Ideators	
	With Attempt	No attempt	With Ideation	No ideation	With Plan	No plan	With Attempt	No attempt
Physical Abuse	24.9 (4.6)	12.2 (0.8)	21.1 (2.5)	11.8 (0.7)	24.3 (4.6)	27.9 (4.0)	24.9 (4.6)	24.5 (3.6)
Sexual Abuse	2.1 (1.2)	0.1 (0.0)	0.7 (0.4)	0.1 (0.0)	1.6 (0.9)	0.0 (0.0)	2.1 (1.2)	0.0 (0.0)
Parent Died	11.6 (2.4)	11.3 (0.6)	13.9 (2.3)	11.3 (0.6)	12.2 (2.4)	16.1 (4.2)	11.6 (2.4)	15.6 (3.8)
Parent Divorced	14.2 (3.8)	4.8 (0.4)	7.9 (1.6)	4.7 (0.4)	9.7 (2.6)	9.2 (3.7)	14.2 (3.8)	6.7 (2.9)
Other Parent Loss	2.1 (1.2)	2.2 (0.4)	3.9 (1.2)	2.1 (0.4)	1.1 (0.6)	3.0 (1.4)	2.1 (1.2)	2.7 (1.3)
Family Violence	4.3 (1.5)	3.0 (0.3)	4.1 (0.9)	2.9 (0.3)	4.7 (1.5)	6.3 (1.8)	4.3 (1.5)	4.5 (1.4)
Physical Illness	5.0 (2.3)	2.5 (0.3)	4.0 (1.2)	2.4 (0.3)	4.4 (1.8)	4.7 (1.8)	5.0 (2.3)	4.3 (1.6)
Financial Adversity	6.1 (2.4)	5.6 (0.5)	4.1 (0.9)	5.8 (0.5)	6.0 (2.1)	3.3 (1.5)	6.1 (2.4)	2.9 (1.0)
1	35.4 (4.2)	23.4 (1.0)	35.9 (2.8)	22.7 (0.9)	32.9 (4.0)	41.7 (5.2)	35.4 (4.2)	40.5 (4.5)
2+	15.4 (3.4)	8.6 (0.5)	10.8 (1.7)	8.6 (0.5)	14.1 (3.2)	13.2 (3.3)	15.4 (3.4)	9.6 (2.3)
a	(140)	(107309)	(394)	(112243)	(171)	(1976)	(140)	(2212)

[a] Number of cases with the outcome variable; N represents the number of person years.
[b] % represents the percentage of people with the adversity among the cases with the outcome variable indicated in the column header. For example: the first cell is the % of those with physical abuse among those with attempts.

For peer review only - http://bmjopen.bmj.com/site/about/guidelines.xhtml

Table 3: Multivariate model for associations between childhood adversities and lifetime suicidality[1]

	**LT Attempts in total sample**[b]		**Ideators among total sample**[c]		**Suicidal Ideators with LT plans** [d]		**Suicidal Ideators with LT attempts** [e]	
	**OR(95% CI)**	**Chi-square**	**OR(95% CI)**	**Chi-square**	**OR(95% CI)**	**Chi-square**	**OR(95% CI)**	**Chi-square**
Physical Abuse	2.0* (1.2-3.3)*	7.4(0.006)*	1.7* (1.3-2.3)*	15.2(<.001)*	0.6 (0.3-1.4)	1.3(0.25)	1.0 (0.5-2.3)	0.0(0.93)
Sexual Abuse	7.6* (2.0-29.9)*	8.9(0.003)*	2.6 (0.6-10.6)	1.8(0.18)	---	---	---	---
Parent Died	1.1 (0.6-1.8)	0.1(0.78)	1.4 (0.9-2.1)	2.7(0.10)	0.7 (0.3-1.7)	0.6(0.45)	0.8 (0.5-1.5)	0.4(0.52)
Parent Divorced	2.7* (1.5-5.0)*	10.8(0.001)*	1.6* (1.0-2.4)*	4.3(0.038)*	0.9 (0.3-3.3)	0.0(0.88)	3.1* (1.2-8.6)*	5.2(0.023)*
Other Parent Loss	1.0 (0.3-3.3)	0.0(0.95)	1.7 (1.0-3.0)	3.6(0.06)	0.4 (0.1-2.6)	0.9(0.34)	2.0 (0.2-17.3)	0.4(0.51)
Family Violence	0.7 (0.3-1.7)	0.6(0.42)	0.8 (0.5-1.4)	0.5(0.47)	1.0 (0.4-2.4)	0.0(0.97)	2.4 (0.9-6.3)	3.5(0.06)
Physical Illness	1.1 (0.4-3.5)	0.1(0.81)	1.2 (0.6-2.3)	0.2(0.63)	0.8 (0.2-3.1)	0.1(0.71)	1.2 (0.3-3.9)	0.1(0.80)
Financial Adversity	1.0 (0.4-2.7)	0.0(0.94)	0.6 (0.4-1.1)	3.0(0.08)	2.4 (0.7-8.4)	1.9(0.17)	2.1 (0.7-6.0)	2.1(0.15)

*Significant at the .05 level, two-sided test
LT: lifetime
[b]Models controls for int(1-5 intervals), demographics (sex, age, time-varying education), interaction between intervals(13-19,20-29,30+) and age, education. For parent psychopathology, controlling for number of parental disorders (dummies for 1, 2+ disorders).
[c]Models controls for int(1-5 intervals), demographics (sex, age, time-varying education), interaction between int intervals(13-19,20-29,30+) and age, education. For parent psychopathology, controlling for number of parental disorders (dummies for 1, 2+ disorders).
[d]Models controls for int(1-5 intervals), demographics (sex, age, time-varying education), interaction between int intervals(13-19,20-29,30+) and age, education. For parent psychopathology, controlling for types of parental disorders (6 dummies).
[e]Models controls for int(1-5 intervals), demographics (sex, age, time-varying education), interaction between int intervals(13-19,20-29,30+) and age, education. Parent psychopathology not controlled for due to insignificance in previous models.

For peer review only - http://bmjopen.bmj.com/site/about/guidelines.xhtml

Table 4: Associations between number of childhood adversities and lifetime suicidality[1]

	LT Attempts in total sample[b]		Ideators among total sample[c]		Ideators with LT plans [d]		Ideators with LT attempts [e]	
Number of child adversities	OR(95% CI)	Chi-square	OR(95% CI)	Chi-square	OR(95% CI)	Chi-square	OR(95% CI)	Chi-square
1	1.9* (1.3-2.8)*		1.8* (1.5-2.3)*		0.5 (0.3-1.0)		0.9 (0.5-1.7)	
2+	2.1* (1.2-3.8)*	14.3(<.001)*	1.4* (1.0-2.0)*	28.3(<.001)*	1.1 (0.3-3.3)	4.5(0.10)	2.7* (1.3-5.9)*	8.3(0.016)*

*Significant at the .05 level, two-sided test
LT: lifetime
[b]Models controls for int(1-5 intervals), demographics (sex, age, time-varying education), interaction between int intervals(13-19,20-29,30+) and age, education. For parent psychopathology, controlling for number of parental disorders (dummies for 1, 2+ disorders).
[c]Models controls for int(1-5 intervals), demographics (sex, age, time-varying education), interaction between int intervals(13-19,20-29,30+) and age, education. For parent psychopathology, controlling for number of parental disorders (dummies for 1, 2+ disorders).
[d]Models controls for int(1-5 intervals), demographics (sex, age, time-varying education), interaction between int intervals(13-19,20-29,30+) and age, education. For parent psychopathology, controlling for types of parental disorders (6 dummies).
[e]Models controls for int(1-5 intervals), demographics (sex, age, time-varying education), interaction between int intervals(13-19,20-29,30+) and age, education. Parent psychopathology not controlled for due to insignificance in previous models.

For peer review only - http://bmjopen.bmj.com/site/about/guidelines.xhtml

Table 5: Final multivariate model for associations between childhood adversities and lifetime suicidality[1]

	LT Attempts in total sample[b]		Ideators among total sample[c]		Ideators with LT plans [d]		Ideators with LT attempts [e]	
	OR(95% CI)	Chi-square	OR(95% CI)	Chi-square	OR(95% CI)	Chi-square	OR(95% CI)	Chi-square
Physical Abuse	2.2* (1.3-3.8)*	8.9(0.003)*	2.1* (1.6-2.8)*	25.4(<.001)*	0.4* (0.2-1.0)*	4.3(0.038)*	0.8 (0.3-2.1)	0.3(0.60)
Sexual Abuse	9.3* (2.5-35.2)*	11.2(<.001)*	3.7 (0.9-15.9)	3.3(0.07)	---	---	---	---
Parent Died	1.2 (0.7-2.3)	0.4(0.51)	1.7* (1.1-2.6)*	6.6(0.010)*	0.4 (0.1-1.3)	2.2(0.14)	0.6 (0.3-1.1)	2.8(0.10)
Parent Divorced	3.1* (1.7-5.6)*	14.5(<.001)*	1.9* (1.2-3.0)*	8.1(0.004)*	0.7 (0.2-2.3)	0.4(0.51)	2.4 (0.9-6.4)	3.0(0.08)
Other Parent Loss	1.1 (0.3-4.3)	0.0(0.87)	2.1* (1.3-3.6)*	8.3(0.004)*	0.3 (0.0-2.0)	1.8(0.18)	1.3 (0.1-13.3)	0.1(0.79)
Family Violence	0.9 (0.3-2.3)	0.1(0.76)	1.1 (0.6-2.3)	0.2(0.69)	0.4 (0.1-1.8)	1.6(0.20)	1.2 (0.4-4.1)	0.1(0.76)
Physical Illness	1.4 (0.4-5.3)	0.2(0.63)	1.6 (0.7-3.3)	1.4(0.24)	0.6 (0.1-2.5)	0.5(0.46)	0.9 (0.2-3.3)	0.0(0.85)
Financial Adversity	1.3 (0.4-3.7)	0.2(0.65)	0.9 (0.4-1.7)	0.1(0.71)	1.6 (0.4-6.0)	0.6(0.44)	1.4 (0.5-4.3)	0.4(0.52)
group significance test for all types		29.4(<.001)*		43.0(<.001)*		833.9(<.001) *		11.5(0.18)
significance test for difference between types		13.1(0.07)		9.2(0.24)		805.7(<.001) *		11.8(0.11)
2+ adversities	0.7 (0.2-1.8)	0.7(0.41)	0.5* (0.3-0.9)*	4.9(0.028)*	4.7 (0.8-29.2)	2.9(0.09)	2.9 (0.8-10.6)	2.7(0.10)

*Significant at the .05 level, two-sided test
LT: lifetime
[b]Models controls for int(1-5 intervals), demographics (sex, age, time-varying education), interaction between int intervals(13-19,20-29,30+) and age, education. For parent psychopathology, controlling for number of parental disorders (dummies for 1, 2+ disorders).
[c]Models controls for int(1-5 intervals), demographics (sex, age, time-varying education), interaction between int intervals(13-19,20-29,30+) and age, education. For parent psychopathology, controlling for number of parental disorders (dummies for 1, 2+ disorders).
[d]Models controls for int(1-5 intervals), demographics (sex, age, time-varying education), interaction between int intervals(13-19,20-29,30+) and age, education. For parent psychopathology, controlling for types of parental disorders (6 dummies).
[e]Models controls for int(1-5 intervals), demographics (sex, age, time-varying education), interaction between int intervals(13-19,20-29,30+) and age, education. Parent psychopathology not controlled for due to insignificance in previous models.

For peer review only - http://bmjopen.bmj.com/site/about/guidelines.xhtml

South Africa Table 6. Interactions between child adversity and lifecourse[1]

		LT Attempts in total sample[2]		Ideators among total sample[3]		Among Ideators, LT Plans[4]		Among Ideators, LT Attempts[5]	
		OR(95% CI)	Chisquare	OR(95% CI)	Chisquare	OR(95% CI)	Chisquare	OR(95% CI)	Chisquare
Interactions									
Physical Abuse	13-19	6.2 (0.6-62.0)	3.9(0.27)	1.0 (0.2-4.2)	13.2(0.004)*	0.8 (0.1-7.3)	0.1(0.97)	---	---
	20-29	2.7 (0.3-24.3)		0.3 (0.1-1.2)		0.9 (0.2-4.0)		---	
	30+	3.7 (0.5-28.4)		0.5 (0.1-2.2)		---		---	
Sexual Abuse	13-19	0.3 (0.0-8.7)	70.1(<.001)*	0.1 (0.0-4.4)	147.3(<.001)*	0.0* (0.0-0.0)*	0.6(0.45)	---	66.0(<.001)*
	20-29	0.1 (0.0-4.5)		0.1 (0.0-2.0)		0.0* (0.0-0.0)*		0.0* (0.0-0.0)*	
	30+	0.0* (0.0-0.0)*		0.0* (0.0-0.0)*		---		---	
Parent Died	13-19	0.7 (0.0-18.9)	1.7(0.63)	1.5 (0.1-18.8)	0.9(0.84)	0.1 (0.0-6.6)	3.5(0.32)	0.0* (0.0-0.0)*	130.0(<.001)*
	20-29	0.2 (0.0-8.3)		0.9 (0.1-12.2)		0.1 (0.0-2.6)		0.0* (0.0-0.0)*	
	30+	0.5 (0.0-16.3)		1.1 (0.1-17.9)		0.0 (0.0-1.4)		0.0* (0.0-0.0)*	
Parent Divorced	13-19	1.6 (0.1-25.5)	2.1(0.56)	0.9 (0.1-10.7)	1.9(0.59)	0.1 (0.0-5.5)	1.8(0.63)	---	---
	20-29	0.6 (0.0-9.1)		0.4 (0.0-4.0)		0.2 (0.0-9.8)		---	
	30+	1.5 (0.1-27.8)		0.8 (0.1-8.9)		0.1 (0.0-7.3)		---	
Other Parent Loss	13-19	0.3 (0.1-1.6)	91.1(<.001)*	45.6* (3.8-540.5)*	100.1(<.001)*	---	---	---	---
	20-29	174.6* (11.5-2640.7)*		746.2* (147.5-3775.9)*		---		---	
	30+	179.8* (16.7-1936.1)*		---		---		---	
Family Violence	13-19	211.4* (11.8-3776.4)*	18.7(<.001)*	1.1 (0.1-13.0)	2.8(0.42)	101.4* (1.1-9315.2)*	4.4(0.11)	0.0* (0.0-0.0)*	0.4(0.84)
	20-29	44.3* (2.1-945.4)*		0.3 (0.0-3.1)		11.5 (0.2-696.2)		0.0* (0.0-0.0)*	

	30+	115.1* (7.2-1831.8)*		0.5 (0.0-4.3)		---		0.0* (0.0-0.0)*	
Physical Illness	13-19	567.1* (34.8-9251.4)*	43.3(<.001)*	1.0 (0.1-13.9)	3.3(0.35)	439.3* (7.8-24619.1)*	12.7(0.002)*	0.0* (0.0-0.0)*	98.1(<.001)*
	20-29	49.1* (1.4-1667.6)*		0.7 (0.1-7.9)		9.1 (0.1-1409.7)		0.1 (0.0-2.8)	
	30+	---		3.0 (0.3-33.0)		---		---	
Financial Adversity	13-19	292.0* (26.1-3266.5)*	43.7(<.001)*	0.3 (0.0-4.9)	2.8(0.42)	---	---	0.0* (0.0-0.0)*	130.0(<.001)*
	20-29	121.5* (15.7-939.8)*		0.3 (0.0-3.3)		---		0.0* (0.0-0.0)*	
	30+	328.9* (34.6-3126.2)*		1.0 (0.1-15.9)		---		0.0* (0.0-0.0)*	
significance test for all child adversities			3313.2(<.001)*		1136.6(<.001)*		588.1(<.001)*		352.5(<.001)*
significance test for all child adversities and number of child adversities			5396.2(<.001)*		2805.8(<.001)*		611.3(<.001)*		352.6(<.001)*
2+ adversities	13-19	0.4 (0.0-15.3)	8.5(0.037)*	2.9 (0.2-49.3)	7.9(0.047)*	0.0 (0.0-1.5)	3.3(0.19)	1.2 (0.0-173.4)	0.1(0.95)
	20-29	5.4 (0.1-224.2)		12.1 (0.9-162.3)		0.2 (0.0-9.8)		1.5 (0.1-23.7)	
	30+	0.4 (0.0-14.6)		2.2 (0.2-31.3)		---		---	

*Significant at the .05 level, two-sided test

[1]Models included all controls and interaction terms between int interval dummies (13-19,20-29,30+) and each control. Only the interaction terms between the int dummies and child adversity variables are shown in table. Assessed in Part 2 sample. Controls for the model include three int dummies (13-19,20-29,30+), and country, and also include significant variables from demographic and parent psychopathology models, details in following footnotes

[2]Models controls for int(1-5 intervals), countries, demographics (sex, age, time-varying education), interaction between int intervals(13-19,20-29,30+) and age, education. For parent psychopathology, controlling for number of parental disorders (dummies for 1, 2+ disorders).

[3]Models controls for int(1-5 intervals), countries, demographics (sex, age, time-varying education), interaction between int intervals(13-19,20-29,30+) and age, education. For parent psychopathology, controlling for number of parental disorders (dummies for 1, 2+ disorders).

[4]Models controls for int(1-5 intervals), countries, demographics (sex, age, time-varying education), interaction between int intervals(13-19,20-29,30+) and age, education. For parent psychopathology, controlling for types of parental disorders (6 dummies).

[5]Models controls for int(1-5 intervals), countries, demographics (sex, age, time-varying education), interaction between int intervals(13-19,20-29,30+) and age, education. Parent psychopathology not controlled for due to insignificance in previous models.

For peer review only - http://bmjopen.bmj.com/site/about/guidelines.xhtml

South Africa Table 6a. Multivariate model for associations between child adversity and LT suicidality during childhood years[1]

	Int range 4-12							
	LT Attempts in total sample[2]		Ideators among total sample[3]		Among Ideators, LT Plans[4]		Among Ideators, LT Attempts[5]	
	OR(95% CI)	Chisquare	OR(95% CI)	Chisquare	OR(95% CI)	Chisquare	OR(95% CI)	Chisquare
Physical Abuse	0.6 (0.1-4.3)	0.3(0.60)	3.7* (1.0-13.4)*	4.2(0.041)*	---	---	0.0* (0.0-0.0)*	1177.3(<.001)*
Sexual Abuse	61.6* (4.5-841.0)*	9.9(0.002)*	34.8* (3.1-392.6)*	8.6(0.003)*	---	---	---	---
Parent Died	2.6 (0.1-52.0)	0.4(0.53)	1.5 (0.1-16.9)	0.1(0.76)	8.6 (0.3-234.7)	1.7(0.19)	22.7* (1.5-338.3)*	5.3(0.021)*
Parent Divorced	3.0 (0.2-38.0)	0.7(0.39)	2.9 (0.3-24.8)	0.9(0.33)	4.6 (0.1-215.6)	0.6(0.43)	0.0* (0.0-0.0)*	135.3(<.001)*
Other Parent Loss	0.0* (0.0-0.0)*	53.4(<.001)*	0.0* (0.0-0.0)*	77.1(<.001)*	---	---	---	---
Family Violence	0.0* (0.0-0.1)*	12.9(<.001)*	1.9 (0.3-13.1)	0.5(0.48)	---	---	---	---
Physical Illness	0.0* (0.0-0.0)*	44.3(<.001)*	1.4 (0.2-13.2)	0.1(0.75)	---	---	---	---
Financial Adversity	0.0* (0.0-0.0)*	64.2(<.001)*	2.0 (0.2-22.3)	0.3(0.57)	0.0* (0.0-0.0)*	26.8(<.001)*	0.0* (0.0-0.2)*	10.0(0.002)*
group significance test for all types		347.6(<.001)*		822.4(<.001)*		204.6(<.001)*		1425.4(<.001)*
significance test for difference between types		301.9(<.001)*		637.3(<.001)*		203.1(<.001)*		1123.3(<.001)*
2+ adversities	0.6 (0.0-13.1)	0.1(0.73)	0.1 (0.0-1.3)	3.2(0.07)	---	---	---	---

*Significant at the .05 level, two-sided test

¹Assessed in Part 2 sample due to having part 2 controls. Controls for the model include int (1-5 intervals), and also include significant variables from

²Models controls for int(1-5 intervals), countries, demographics (sex, age, time-varying education), interaction between int intervals(13-19,20-29,30+) and age, education. For parent psychopathology, controlling for number of parental disorders (dummies for 1, 2+ disorders).

[3]Models controls for int(1-5 intervals), countries, demographics (sex, age, time-varying education), interaction between int intervals(13-19,20-29,30+) and age, education. For parent psychopathology, controlling for number of parental disorders (dummies for 1, 2+ disorders).

[4]Models controls for int(1-5 intervals), countries, demographics (sex, age, time-varying education), interaction between int intervals(13-19,20-29,30+) and age, education. For parent psychopathology, controlling for types of parental disorders (6 dummies).

[5]Models controls for int(1-5 intervals), countries, demographics (sex, age, time-varying education), interaction between int intervals(13-19,20-29,30+) and age, education. Parent psychopathology not controlled for due to insignificance in previous models.

For peer review only

For peer review only

South Africa Table 6b. Multivariate model for associations between child adversity and LT suicidality during teen years[1]

	Int range 13-19							
	LT Attempts in total sample[2]		Ideators among total sample[3]		Among Ideators, LT Plans[4]		Among Ideators, LT Attempts[5]	
	OR(95% CI)	Chisquare	OR(95% CI)	Chisquare	OR(95% CI)	Chisquare	OR(95% CI)	Chisquare
Physical Abuse	3.7* (1.5-9.2)*	8.5(0.004)*	3.6* (2.2-5.9)*	26.1(<.001)*	0.3 (0.0-2.5)	1.2(0.28)	1.1 (0.3-4.7)	0.0(0.90)
Sexual Abuse	20.3* (2.0-210.2)*	6.6(0.010)*	4.6 (0.3-61.6)	1.4(0.24)	---	---	---	---
Parent Died	1.8 (0.5-6.5)	0.9(0.35)	2.2* (1.1-4.3)*	5.3(0.021)*	0.7 (0.1-6.8)	0.1(0.72)	0.5 (0.1-2.2)	0.8(0.38)
Parent Divorced	4.6* (1.7-12.1)*	9.8(0.002)*	2.5 (1.0-6.1)	3.8(0.05)	0.4 (0.1-3.1)	0.8(0.37)	4.3* (1.1-17.0)*	4.5(0.035)*
Other Parent Loss	0.0* (0.0-0.0)*	238.2(<.001)*	0.2 (0.0-1.5)	2.6(0.11)	---	---	0.0* (0.0-0.0)*	60.3(<.001)*
Family Violence	1.9 (0.5-7.2)	1.0(0.33)	2.1 (0.6-7.6)	1.5(0.23)	2.3 (0.1-46.2)	0.3(0.59)	0.9 (0.1-5.7)	0.0(0.89)
Physical Illness	2.9 (0.3-27.8)	0.9(0.34)	1.5 (0.4-5.4)	0.3(0.56)	9.9* (1.8-54.0)*	7.3(0.007)*	1.5 (0.2-11.6)	0.1(0.71)
Financial Adversity	1.9 (0.2-14.5)	0.4(0.53)	0.6 (0.2-2.3)	0.6(0.45)	1.0 (0.1-19.3)	0.0(1.00)	4.0 (0.4-42.9)	1.3(0.25)
group significance test for all types		1168.3(<.001)*		37.5(<.001)*		421.4(<.001)*		1337.0(<.001)*
significance test for difference between types		1004.7(<.001)*		12.4(0.09)		374.6(<.001)*		1283.5(<.001)*
2+ adversities	0.2 (0.0-1.4)	2.6(0.11)	0.3 (0.1-1.0)	3.9(0.048)*	0.9 (0.0-32.5)	0.0(0.93)	2.5 (0.3-18.7)	0.9(0.35)

*Significant at the .05 level, two-sided test

[1]Assessed in Part 2 sample due to having part 2 controls. Controls for the model include int (1-5 intervals), and also include significant variables from

[2]Models controls for int(1-5 intervals), countries, demographics (sex, age, time-varying education), interaction between int intervals(13-19,20-29,30+) and age, education. For parent psychopathology, controlling for number of parental disorders (dummies for 1, 2+ disorders).

[3]Models controls for int(1-5 intervals), countries, demographics (sex, age, time-varying education), interaction between int intervals(13-19,20-29,30+) and age, education. For parent psychopathology, controlling for number of parental disorders (dummies for 1, 2+ disorders).

[4]Models controls for int(1-5 intervals), countries, demographics (sex, age, time-varying education), interaction between int intervals(13-19,20-29,30+) and age, education. For parent psychopathology, controlling for types of parental disorders (6 dummies).

[5]Models controls for int(1-5 intervals), countries, demographics (sex, age, time-varying education), interaction between int intervals(13-19,20-29,30+) and age, education. Parent psychopathology not controlled for due to insignificance in previous models.

For peer review only

South Africa Table 6c. Multivariate model for associations between child adversity and LT suicidality during young adult years[1]

	Int range 20-29							
	LT Attempts in total sample[2]		Ideators among total sample[3]		Among Ideators, LT Plans[4]		Among Ideators, LT Attempts[5]	
	OR(95% CI)	Chisquare	OR(95% CI)	Chisquare	OR(95% CI)	Chisquare	OR(95% CI)	Chisquare
Physical Abuse	1.6 (0.8-3.5)	1.7(0.20)	1.1 (0.7-1.8)	0.2(0.64)	0.4 (0.1-1.2)	2.7(0.10)	0.9 (0.3-3.2)	0.0(0.88)
Sexual Abuse	5.1 (0.4-66.1)	1.6(0.20)	2.2 (0.3-17.5)	0.5(0.46)	---	---	0.0* (0.0-0.0)*	68.1(<.001)*
Parent Died	0.6 (0.2-2.4)	0.4(0.51)	1.4 (0.7-2.8)	0.7(0.40)	0.4 (0.0-2.8)	1.0(0.32)	0.6 (0.2-2.2)	0.7(0.42)
Parent Divorced	1.7 (0.7-4.5)	1.3(0.25)	1.1 (0.5-2.6)	0.1(0.74)	0.8 (0.2-4.2)	0.0(0.84)	2.9 (0.7-12.7)	2.2(0.14)
Other Parent Loss	1.2 (0.2-7.6)	0.1(0.82)	2.9* (1.2-7.4)*	5.5(0.019)*	0.1 (0.0-1.5)	3.0(0.08)	10.5 (0.7-160.1)	3.0(0.09)
Family Violence	0.4 (0.1-1.5)	2.0(0.16)	0.5 (0.2-1.8)	1.1(0.30)	0.3 (0.0-2.6)	1.4(0.24)	2.1 (0.2-25.7)	0.4(0.55)
Physical Illness	0.3 (0.0-5.1)	0.8(0.36)	1.0 (0.4-2.6)	0.0(0.96)	0.2 (0.0-4.6)	1.0(0.32)	0.1 (0.0-1.4)	3.0(0.08)
Financial Adversity	0.8 (0.2-3.6)	0.1(0.76)	0.5 (0.2-1.4)	1.9(0.17)	1.7 (0.3-10.9)	0.3(0.57)	1.3 (0.2-7.7)	0.1(0.78)
group significance test for all types		9.9(0.27)		9.6(0.30)		1038.1(<.001)*		97.5(<.001)*
significance test for difference between types		5.2(0.64)		10.2(0.18)		973.6(<.001)*		99.6(<.001)*
2+ adversities	3.1 (0.8-12.3)	2.7(0.10)	1.3 (0.7-2.6)	0.7(0.41)	9.1 (0.5-169.9)	2.3(0.13)	3.5 (0.4-28.8)	1.4(0.24)

*Significant at the .05 level, two-sided test

¹Assessed in Part 2 sample due to having part 2 controls. Controls for the model include int (1-5 intervals), and also include significant variables from

²Models controls for int(1-5 intervals), countries, demographics (sex, age, time-varying education), interaction between int intervals(13-19,20-29,30+) and age, education. For parent psychopathology, controlling for number of parental disorders (dummies for 1, 2+ disorders).

³Models controls for int(1-5 intervals), countries, demographics (sex, age, time-varying education), interaction between int intervals(13-19,20-29,30+) and age, education. For parent psychopathology, controlling for number of parental disorders (dummies for 1, 2+ disorders).

[4]Models controls for int(1-5 intervals), countries, demographics (sex, age, time-varying education), interaction between int intervals(13-19,20-29,30+) and age, education. For parent psychopathology, controlling for types of parental disorders (6 dummies).

[5]Models controls for int(1-5 intervals), countries, demographics (sex, age, time-varying education), interaction between int intervals(13-19,20-29,30+) and age, education. Parent psychopathology not controlled for due to insignificance in previous models.

For peer review only

South Africa Table 6d. Multivariate model for associations between child adversity and LT suicidality during later adult years[1]

	Int range 20-29							
	LT Attempts in total sample[2]		Ideators among total sample[3]		Among Ideators, LT Plans[4]		Among Ideators, LT Attempts[5]	
	OR(95% CI)	Chisquare	OR(95% CI)	Chisquare	OR(95% CI)	Chisquare	OR(95% CI)	Chisquare
Physical Abuse	2.2* (1.0-4.8)*	4.4(0.035)*	1.8 (0.9-3.5)	3.2(0.07)	0.4 (0.1-1.2)	2.8(0.10)	1.4 (0.4-5.3)	0.2(0.65)
Sexual Abuse	0.0* (0.0-0.0)*	81.7(<.001)*	0.0* (0.0-0.0)*	218.1(<.001)*	---	---	0.8 (0.0-16.7)	0.0(0.88)
Parent Died	1.3 (0.3-4.9)	0.1(0.70)	1.6 (0.7-3.6)	1.5(0.22)	0.2 (0.0-1.6)	2.3(0.13)	0.6 (0.2-1.9)	0.8(0.37)
Parent Divorced	4.6 (1.0-21.6)	3.9(0.049)*	2.4 (0.9-6.2)	3.5(0.06)	0.3 (0.0-2.5)	1.2(0.28)	1.9 (0.1-31.6)	0.2(0.65)
Other Parent Loss	1.3 (0.2-9.4)	0.1(0.80)	5.1* (2.1-12.1)*	14.1(<.001)*	0.2 (0.0-4.0)	1.1(0.30)	0.6 (0.1-6.9)	0.2(0.70)
Family Violence	1.0 (0.2-6.5)	0.0(0.96)	0.9 (0.2-3.4)	0.0(0.86)	0.0* (0.0-0.9)*	4.3(0.037)*	0.8 (0.0-25.2)	0.0(0.92)
Physical Illness	5.5 (0.9-32.1)	3.7(0.05)	4.3* (1.1-15.9)*	4.8(0.028)*	0.0* (0.0-0.9)*	4.1(0.042)*	1.6 (0.1-20.8)	0.1(0.73)
Financial Adversity	2.1 (0.3-15.5)	0.6(0.44)	2.0 (0.5-8.4)	1.0(0.31)	0.7 (0.1-4.9)	0.1(0.75)	0.8 (0.1-4.6)	0.1(0.78)
group significance test for all types		338.1(<.001)*		525.7(<.001)*		7.9(0.34)		5.9(0.66)
significance test for difference between types		272.0(<.001)*		477.0(<.001)*		4.8(0.57)		5.7(0.57)
2+ adversities	0.2 (0.0-1.6)	2.4(0.12)	0.2 (0.1-1.1)	3.6(0.06)	44.5* (2.5-779.1)*	7.0(0.008)*	2.1 (0.2-18.5)	0.5(0.49)

*Significant at the .05 level, two-sided test

¹Assessed in Part 2 sample due to having part 2 controls. Controls for the model include int (1-5 intervals), and also include significant variables from

²Models controls for int(1-5 intervals), countries, demographics (sex, age, time-varying education), interaction between int intervals(13-19,20-29,30+) and age, education. For parent psychopathology, controlling for number of parental disorders (dummies for 1, 2+ disorders).

[3]Models controls for int(1-5 intervals), countries, demographics (sex, age, time-varying education), interaction between int intervals(13-19,20-29,30+) and age, education. For parent psychopathology, controlling for number of parental disorders (dummies for 1, 2+ disorders).

[4]Models controls for int(1-5 intervals), countries, demographics (sex, age, time-varying education), interaction between int intervals(13-19,20-29,30+) and age, education. For parent psychopathology, controlling for types of parental disorders (6 dummies).

[5]Models controls for int(1-5 intervals), countries, demographics (sex, age, time-varying education), interaction between int intervals(13-19,20-29,30+) and age, education. Parent psychopathology not controlled for due to insignificance in previous models.

For peer review only

South Africa Table 7. Bivariate model for associations between child adversities and LT suicidality[1]

	LT Attempts in total sample[2]		Ideators among total sample[3]		Among Ideators, LT Plans[4]		Among Ideators, LT Attempts[5]	
	OR(95% CI)	Chisquare	OR(95% CI)	Chisquare	OR(95% CI)	Chisquare	OR(95% CI)	Chisquare
Physical Abuse	2.0* (1.2-3.2)*	7.3(0.007)*	1.7* (1.3-2.3)*	16.7(<.001)*	0.7 (0.3-1.4)	1.2(0.26)	1.1 (0.5-2.5)	0.1(0.81)
Sexual Abuse	7.9* (1.9-32.1)*	8.6(0.003)*	3.0 (0.7-12.2)	2.5(0.11)	---	---	---	---
Parent Died	1.1 (0.7-1.7)	0.1(0.76)	1.3 (0.9-1.9)	2.0(0.16)	0.8 (0.4-1.9)	0.3(0.62)	0.8 (0.4-1.5)	0.4(0.53)
Parent Divorced	2.8* (1.5-5.2)*	11.4(<.001)*	1.5 (1.0-2.3)	3.7(0.05)	1.2 (0.4-3.8)	0.1(0.78)	3.0* (1.1-8.0)*	4.9(0.027)*
Other Parent Loss	0.9 (0.3-2.8)	0.1(0.81)	1.6 (0.9-2.7)	2.9(0.09)	0.5 (0.1-2.7)	0.7(0.41)	2.5 (0.6-11.0)	1.5(0.22)
Family Violence	1.0 (0.4-2.2)	0.0(0.98)	1.1 (0.6-1.8)	0.0(0.83)	0.8 (0.4-2.0)	0.2(0.68)	2.2 (0.9-5.5)	2.9(0.09)
Physical Illness	1.5 (0.6-4.1)	0.7(0.39)	1.3 (0.7-2.4)	0.7(0.42)	0.9 (0.2-3.5)	0.0(0.86)	1.2 (0.4-4.0)	0.1(0.77)
Financial Adversity	1.2 (0.5-2.8)	0.1(0.73)	0.7 (0.4-1.2)	1.4(0.23)	1.9 (0.6-6.8)	1.1(0.29)	2.0 (0.7-6.3)	1.6(0.21)

*Significant at the .05 level, two-sided test

[1]Assessed in Part 2 sample due to having part 2 controls. Controls for the model include int (1-5 intervals), and also include significant variables from

[2]Models controls for int(1-5 intervals), countries, demographics (sex, age, time-varying education), interaction between int intervals(13-19,20-29,30+) and age, education. For parent psychopathology, controlling for number of parental disorders (dummies for 1, 2+ disorders).

[3]Models controls for int(1-5 intervals), countries, demographics (sex, age, time-varying education), interaction between int intervals(13-19,20-29,30+) and age, education. For parent psychopathology, controlling for number of parental disorders (dummies for 1, 2+ disorders).

For peer review only - http://bmjopen.bmj.com/site/about/guidelines.xhtml

[4]Models controls for int(1-5 intervals), countries, demographics (sex, age, time-varying education), interaction between int intervals(13-19,20-29,30+) and age, education. For parent psychopathology, controlling for types of parental disorders (6 dummies).

[5]Models controls for int(1-5 intervals), countries, demographics (sex, age, time-varying education), interaction between int intervals(13-19,20-29,30+) and age, education. Parent psychopathology not controlled for due to insignificance in previous models.

For peer review only

STROBE Statement—checklist of items that should be included in reports of observational studies

	Item No	Recommendation
**Title and abstract**	1	(*a*) Indicate the study's design with a commonly used term in the title or the abstract
		(*b*) Provide in the abstract an informative and balanced summary of what was done and what was found
**Introduction**		
Background/rationale	2	Explain the scientific background and rationale for the investigation being reported
Objectives	3	State specific objectives, including any prespecified hypotheses
**Methods**		
Study design	4	Present key elements of study design early in the paper
Setting	5	Describe the setting, locations, and relevant dates, including periods of recruitment, exposure, follow-up, and data collection
Participants	6	(*a*) *Cohort study*—Give the eligibility criteria, and the sources and methods of selection of participants. Describe methods of follow-up<br>*Case-control study*—Give the eligibility criteria, and the sources and methods of case ascertainment and control selection. Give the rationale for the choice of cases and controls<br>*Cross-sectional study*—Give the eligibility criteria, and the sources and methods of selection of participants
		(*b*) *Cohort study*—For matched studies, give matching criteria and number of exposed and unexposed<br>*Case-control study*—For matched studies, give matching criteria and the number of controls per case
Variables	7	Clearly define all outcomes, exposures, predictors, potential confounders, and effect modifiers. Give diagnostic criteria, if applicable
Data sources/ measurement	8*	For each variable of interest, give sources of data and details of methods of assessment (measurement). Describe comparability of assessment methods if there is more than one group
Bias	9	Describe any efforts to address potential sources of bias
Study size	10	Explain how the study size was arrived at
Quantitative variables	11	Explain how quantitative variables were handled in the analyses. If applicable, describe which groupings were chosen and why
Statistical methods	12	(*a*) Describe all statistical methods, including those used to control for confounding
		(*b*) Describe any methods used to examine subgroups and interactions
		(*c*) Explain how missing data were addressed
		(*d*) *Cohort study*—If applicable, explain how loss to follow-up was addressed<br>*Case-control study*—If applicable, explain how matching of cases and controls was addressed<br>*Cross-sectional study*—If applicable, describe analytical methods taking account of sampling strategy
		(*e*) Describe any sensitivity analyses

Continued on next page

For peer review only - http://bmjopen.bmj.com/site/about/guidelines.xhtml

**Results**		
Participants	13*	(a) Report numbers of individuals at each stage of study—eg numbers potentially eligible, examined for eligibility, confirmed eligible, included in the study, completing follow-up, and analysed
		(b) Give reasons for non-participation at each stage
		(c) Consider use of a flow diagram
Descriptive data	14*	(a) Give characteristics of study participants (eg demographic, clinical, social) and information on exposures and potential confounders
		(b) Indicate number of participants with missing data for each variable of interest
		(c) *Cohort study*—Summarise follow-up time (eg, average and total amount)
Outcome data	15*	*Cohort study*—Report numbers of outcome events or summary measures over time
		Case-control study—Report numbers in each exposure category, or summary measures of exposure
		Cross-sectional study—Report numbers of outcome events or summary measures
Main results	16	(*a*) Give unadjusted estimates and, if applicable, confounder-adjusted estimates and their precision (eg, 95% confidence interval). Make clear which confounders were adjusted for and why they were included
		(*b*) Report category boundaries when continuous variables were categorized
		(*c*) If relevant, consider translating estimates of relative risk into absolute risk for a meaningful time period
Other analyses	17	Report other analyses done—eg analyses of subgroups and interactions, and sensitivity analyses
**Discussion**		
Key results	18	Summarise key results with reference to study objectives
Limitations	19	Discuss limitations of the study, taking into account sources of potential bias or imprecision. Discuss both direction and magnitude of any potential bias
Interpretation	20	Give a cautious overall interpretation of results considering objectives, limitations, multiplicity of analyses, results from similar studies, and other relevant evidence
Generalisability	21	Discuss the generalisability (external validity) of the study results
**Other information**		
Funding	22	Give the source of funding and the role of the funders for the present study and, if applicable, for the original study on which the present article is based

*Give information separately for cases and controls in case-control studies and, if applicable, for exposed and unexposed groups in cohort and cross-sectional studies.

Note: An Explanation and Elaboration article discusses each checklist item and gives methodological background and published examples of transparent reporting. The STROBE checklist is best used in conjunction with this article (freely available on the Web sites of PLoS Medicine at http://www.plosmedicine.org/, Annals of Internal Medicine at http://www.annals.org/, and Epidemiology at http://www.epidem.com/). Information on the STROBE Initiative is available at www.strobe-statement.org.

For peer review only

Association between childhood adversities and long-term suicidality among South Africans: Results from the South African Stress and Health Study

Journal:	*BMJ Open*
Manuscript ID:	bmjopen-2013-004644.R1
Article Type:	Research
Date Submitted by the Author:	28-Feb-2014
Complete List of Authors:	Bruwer, Belinda; Stellenbosch University, Psychiatry<br>Govender, Ravi; Stellenbosch University, Psychiatry<br>Bishop, Melanie; Stellenbosch University, Psychiatry<br>Williams, David; Harvard University, Human Development and Health<br>Stein, Dan; University of Cape Town, Psychiatry and mental health<br>Seedat, Soraya; Stellenbosch University, Psychiatry
**Primary Subject Heading**:	Mental health
Secondary Subject Heading:	Mental health
Keywords:	MENTAL HEALTH, Child & adolescent psychiatry < PSYCHIATRY, Adult psychiatry < PSYCHIATRY, PSYCHIATRY, Suicide & self-harm < PSYCHIATRY

SCHOLARONE™
Manuscripts

For peer review only

Association between childhood adversities and suicidality

Association between childhood adversities and long-term suicidality among South Africans: Results from the South African Stress and Health Study

Belinda Bruwer*[a], Ravi Govender[a], Melanie Bishop[a], David R Williams[b] Dan J Stein[c], Soraya Seedat[a]

[a] Department of Psychiatry, Stellenbosch University, PO Box 19063, Tygerberg, 7505, Republic of South Africa.

[b] Department of Society, Human Development and Health, Harvard School of Public Health, and Department of African and African American Studies, Harvard University. Department of Society, Human Development and Health, 677 Huntington Avenue, 6th floor, Boston, MA 02115, United States of America

[c] Department of Psychiatry and Mental Health, University of Cape Town, Groote Schuur Hospital (J2), Anzio Road, Observatory, 7925, Cape Town, Republic of South Africa

*Corresponding author: Department of Psychiatry, University of Stellenbosch, PO Box 19063, Tygerberg, 7505, Republic of South Africa.

Tel nr: +27219404467. Fax nr: +27219404543, **bbruwer@sun.ac.za**

Keywords: Childhood adversities, suicidal ideation, suicidal attempts, suicide, suicidal behaviour

Word count: 5349

For peer review only

Association between childhood adversities and suicidality

ABSTRACT

Objective:

Suicide and suicidal behaviours are significant public health problems and a leading cause of death worldwide and in South Africa. We examined the association between childhood adversities and suicidal behaviour over the life course.

Methods:

A national probability sample of 4,351 South African adult participants (aged 18 years and older) in the South African Stress and Health (SASH) study was interviewed, as part of the World Mental Health Survey initiative. Respondents provided socio-demographic and diagnostic information, as well as an account of suicide-related thoughts and behaviours. Outcomes were defined as suicide attempts and suicidal ideation in the total sample, and suicide plans and attempts among ideators. Childhood adversities included physical abuse, sexual abuse, parental death, parental divorce, other parental loss, family violence, physical illness and financial adversity. The association between suicidality and childhood adversities was examined using discrete-time survival models.

Results:

More than a third of respondents with suicidal behaviour experienced at least 1 childhood adversity, with physical abuse, parental death and parental divorce the most prevalent adversities. Physical abuse, sexual abuse and parental divorce were identified as significant risk markers for lifetime suicide attempts, while physical abuse and parental divorce were significantly correlated with suicidal ideation. Two or more childhood adversities were associated with a 2-fold higher risk of lifetime suicide attempts. Sexual abuse (OR=9.3, childhood, parental divorce (OR=3.1)

For peer review only

and physical abuse (OR=2.2) had the strongest associations with lifetime suicide attempts. The effect of childhood adversities on suicidal tendencies varied over the *life course.* For example, sexual abuse was significantly associated with suicide attempts during childhood and teen years, but not during young and later adulthood.

Conclusions:

Childhood adversities, especially sexual abuse, physical abuse and parental divorce are important risk factors for the onset and persistence of suicidal behaviour, with this risk greatest in childhood and adolescence. The risk for suicidal behaviour was greatest in childhood and adolescence. Suicidal risk in childhood and adolescence was significantly associated with the following childhood adversities: sexual abuse, physical abuse and parental divorce.

Keywords: Childhood adversities, suicidal ideation, suicidal attempts

For peer review only

Association between childhood adversities and suicidality

Strengths and limitations

- These findings extend previous work done in other developing countries that have found childhood adversities to be a significant risk factor for suicidality (20; 57; 58; 59).
- Recall bias might have impacted on the accuracy of recall of childhood adversities.
- Variables such as culture, ethnicity and mental status at the time of the interview might have influenced the recall and reporting of suicidal behaviour.
- Owing to the cross-sectional design, details regarding childhood adversities and suicidal incidents were not assessed. Some of the participants might have been scared to tell the interviewers about their suicidal behaviours. Stigma associated with mental health may have also played a role in reporting suicidal tendencies. The status of the participant's mental health, the role of ethnicity, culture and generational factors may have also contributed to the under-reporting of suicidality.
- The survey was conducted in adults living in households and hostel quarters thus the findings are not generalizable to homeless and institutionalized persons who were not included in the survey.
- The CIDI instrument which was used in this study is a lay-administered instrument which does not include an assessment of several key DSM-IV diagnoses (such as bipolar disorder and psychosis), are associated with elevated rates of suicidality. As a result, some participants with suicidality may have not have been diagnosed with a disorder.

For peer review only

Association between childhood adversities and suicidality

- In view of the large confidence intervals and small sample sizes for some of these analyses caution is required in drawing conclusions.
- We did not control for other unmeasured causes of childhood adversities and suicidailty, or protective (resiliency) factors that may have contributed to the associations observed in these data.

For peer review only

Association between childhood adversities and suicidality

INTRODUCTION

Suicide and suicidal behaviour are significant public health problems. Suicide is one of the leading causes of death worldwide with almost 1 million people committing suicide each year [1]. This figure is likely to grow to approximately 1.2 million suicides in 2020 [2]. In South Africa, the annual rate of suicide is high [3, 4] mirroring international trends [5]. So, too, are rates of suicidal behaviour with an estimated prevalence of 9.1% for lifetime suicidal ideation and 2.9% for suicide attempts among South Africans according to the South African Stress and Health Survey (SASH) [6].

Despite the enormity of the problem, the aetiology of suicidal behaviour is not fully understood. There are controversies in the literature regarding prior psychiatric disorder and risk for suicide attempts. While some authors have argued that pre-existing disorder is an important risk factor (7-11], others have argued that suicide attempts are not neccessarily associated with prior psychopathology [12]. Genetic factors also play an important role in suicidal behaviour [13-16]. While there is stronger evidence pointing towards environmental or experiential factors [17, 18] such as exposure to childhood adversities (19-28]. Recent multi-level country data from the World Mental Health Surveys (WMHS) initiative has allowed for cross-national comparisons of suicidality. The WMHS investigated the association between childhood adversities and suicidal behaviour [20], the persistence of suicidality over time, and the extent to which associations between childhood trauma and suicidality changed over the life course. The WMHS found a dose-response relationship between the number of adversities and suicidal behaviour. Sexual abuse and physical abuse were the strongest risk factors for both the onset and persistence of suicidal behaviours, with the risk for suicidality greatest during childhood (age 4-12 years) and adolescence (age 13-19 years) [20].

For peer review only

Association between childhood adversities and suicidality

Numerous studies have examined the link between childhood sexual abuse and suicidality [29-41]. All of these authors have found that exposure to childhood sexual abuse increases the risk for mental disorders, including suicidality. Furthermore, the majority of studies that have focused on the link between childhood physical abuse and suicidality have found that exposure to childhood physical abuse increases the risk for suicidality [42, 43]. There also appears to be an association between the number of childhood adversities experienced and the later suicidal behaviour [21, 23, 24, 44, 45].

Exposure to early life stress is prevalent among South Africans. In one sample of South African rural youth, the prevalence of physical and sexual abuse was shown to be very high with 94.4% of males exposed to physical abuse and 39.1% of females to sexual abuse [46]. More than a quarter of adults who were interviewed endorsed exposure to childhood adversity (parental death, parental separation or parental divorce) in the SASH study [47]. Significantly more females were prone to be victims of domestic violence than men [47]. Women also reported twice as many suicidal attempts than the male participants in the SASH study [9].

Objective

We report in more detail on data from a South African dataset gathered as part of the World Mental Health Surveys, which allowed for comparison with data from the overall cross-national sample. This data are particularly interesting as South Africa is a middle income African country with high rates of violent trauma exposure. The present study aimed to examine the relationship between the type and frequency of childhood adversity exposure to suicidal behaviour over the life trajectory of South Africans, given that there are no published nationally representative data that may be useful in informing both clinical practice and policy.

For peer review only

Association between childhood adversities and suicidality

METHODS

Sample

Data for the SASH Study were collected between January 2002 and June 2004. WMH surveys were carried out in 21 countries which included Nigeria and South Africa [48]. For detailed information on study methods see Williams et al. (2004) [48]. The research protocol for the SASH study was approved by the Human Subjects Committee of the University of Michigan, by Harvard Medical School ethics committee and by a single project assurance of compliance from the Medical University of South Africa (MEDUNSA), and by the National Institute of Mental Health. It was a national probability sample of 4,351 South African adults (persons aged 18 years and older) living in households or in hostel accommodation. All racial and ethnic groups were represented, with the sample selected using a three-stage probability sample design. The response rate was 85.5%.

Sampling approach

Sampling was divided into three stages. Primary sampling units was selected during the first stage, which was based on the 2001 SA census Enumeration Areas (EAs). The second stage involved sampling of household units within clusters selected in each EA. South Africans in both urban and rural areas were sampled. Sampled residences were stratified into 10 diverse housing categories: Rural–commercial, agricultural, rural traditional subsistence areas, African townships, informal urban or peri-urban shack areas, Coloured townships, Indian townships, general metropolitan residential areas, general large metropolitan residential areas, and domestic servant accommodation in urban areas. During the third stage, one adult respondent in each sampled housing unit was selected. A total of 5089 households was selected. Field interviews were conducted with 4433 (87.1%) of designated respondents. Based on quality control, 4351

For peer review only

interviews were retained for use in the analysis. There were no differences in response rates across the four designated racial groups (white, Coloured [mixed racial origin], Indian, black). According to the 2001 Census statistics, 79.% people in South Africa are Black African, 8.9% are coloured, 9.6% are white, and 2.5% are Indian/Asian [49].

Diagnostic Interview

SASH used version 3 of the World Health Organization Composite Diagnostic Interview (WHO CIDI) [50]. Interviewers were trained within a one week period and conducted the interviews in seven different languages, namely English, Afrikaans, Zulu, Xhosa, Northern Sotho, Southern Sotho, and Tswana. Translations of the CIDI into several native South African languages were conducted in accordance with WHO requirements. Multilingual and bilingual expert panels conducted the back-translations [51, 52]. Informed consent was obtained from participants after a complete description of the study was provided. Respondents provided socio-demographic and diagnostic information, as well as an account of suicidal behaviours during the interviews. The core diagnostic assessment of mental disorders included anxiety disorders (panic disorder, agoraphobia, social phobia, generalized anxiety disorder, post-traumatic stress disorder), mood disorders (major depressive disorder, dysthymia), substance use disorders (alcohol abuse, alcohol dependence, drug abuse, drug dependence) and intermittent explosive disorder [53, 54].

Suicidal behaviour

The CIDI 3.0 module on suicidal behaviour was used to assess the age-of-first-onset, age of most recent episode, and lifetime occurrence of suicidal ideation, suicide plans and suicide attempts. Suicidal ideation, suicide plans and suicide attempts was assessed with questions such as "Have you ever seriously thought about committing suicide?", "Have you ever made a plan for committing suicide?", and "Have you ever attempted suicide?", respectively. Ideators only

For peer review only

proceeded to answer questions about plans ("Have you ever made a plan for committing suicide?") and attempts ("Have you ever attempted suicide?"). Information on the age of first occurrence of the three main outcomes was obtained. To get a better understanding of the progression from ideation to attempt, the outcomes considered in this study were: suicide attempts in the total sample; suicide ideation in the total sample; suicide plans among ideators; suicide attempts among ideators with a plan (planned attempts), and suicide attempts among ideators in the absence of a plan (unplanned or impulsive attempts).

Childhood adversities

Physical abuse, sexual abuse, parental death, parental divorce, other parental loss, family violence, physical illness and financial adversity were the various childhood adversities assessed. Biological and non-biological parents were included in measures of parental death, divorce or other parental loss. Financial adversities were assessed with questions on whether the family had insufficient funds to pay for basic necessities. Questions about repeated fondling, attempted rape or rape were asked to assess for sexual abuse. This comprised the following "The next 2 questions are about sexual assault: (i) The first is about rape. We define this as someone either having sexual intercourse with you or penetrating your body with a finger or object when you did not want them to, either by threatening you or using force, or when you were so young that you didn't know what was happening. Did this ever happen to you?", and (ii)"Other than rape, were you ever sexually assaulted or molested?". A modified version of the Conflict Tactics Scale (CTS2) was used to assess family violence and physical abuse [55]. Respondents were classified as having experienced *physical abuse* when they indicated that, when they were growing up, their father or mother (includes biological, step, or adoptive parents) slapped, hit, pushed, grabbed, shoved, or threw something at them, or that they were beaten as a child by the persons

For peer review only

who raised them. Family violence was assessed as present when respondents indicated that they (i) "were often hit, shoved, pushed, grabbed, or slapped while growing up" *or* (ii) "witnessed physical fights at home, like when your father beat up your mother?" A standard chronic conditions checklist assessed for life-threatening physical illnesses in childhood [56].

Data analysis

All data analyses were processed and analysed centrally by a team of statisticians at the Harvard School of Public Health (Boston, USA) using the SAS version 9.1.3 software package. Discrete-time survival analysis with time-varying covariates was used to study the risk factors of lifetime suicide ideation, plans and attempts. Data were weighted to adjust for the stratified multistage sample design, differential probability of selection within households as a function of household size and clustering of data, and differential non-response. Overall, percentages were weighted to adjust for differences in selection probabilities, differential non-response, oversampling of cases, and residual differences on sociodemographic variables between the sample and the population [48, 57]. A post-stratification weight was also used to make the sample distribution comparable, for age, sex, and province, with the population distribution in the 2001 South African census. Both weighted and geographic clustering of data were taken into account in the data analyses by using a jackknife repeated replications simulation method implemented in SAS macro 14. The survival coefficients were exponentiated and are reported below in the form of odds ratios.

The association between suicidality and childhood adversity was examined using discrete-time survival models with the analysis unit being person-years. Bivariate analyses (considering one adversity at a time) and multivariate analyses (considering all adversities simultaneously) were conducted. Two types of multivariate models were tested: multivariate additive models (simultaneously considering all childhood adversities) and multivariate interactive models (with

For peer review only

number and type of childhood adversities experienced by each respondent included as dummy variables). The analysis also examined interactions between the life stage (13-19 years, 20-29 years, 30+ years) of respondents and each childhood adversity, as well as the influence each adversity had on early-, middle- and later- onset suicidality. Analyses were conducted using SUDAAN version 8.1 to adjust for clustering and weighting. Odds ratios (ORs) with a 95% confidence interval (CIs) are reported. Wald X^2- tests were used to examine multivariate significance. Associations between adversities and suicide outcomes were adjusted for sex , age, educational level, marital status, interactions between demographic variables, life course and parental psychopathology. Analyses also examined the influence of respondents' lifetime mental disorders on suicidality, as well as interactions between sex and each childhood adversity. Statistical significance using two-sided tests was set at p <.05 [20].

RESULTS

Demographic details

In the sample, (n = 4351), there were slightly more female (53.7%) than male respondents. There were more black (76.2%) than coloured (10.4%), white (10%), and Indian/Asian (3.4%) respondents. Furthermore, half of the sample was married and most were unemployed (69.2%), had less than 12 years of education (62.7%) and lived in an urban area (59.7%) (see Table 1).

Prevalence of childhood adversities among the total sample

Figure 1 provides a schematic representation of the suicidality data reported in the sections which follow. In the total sample, 35.4% of participants with one adversity had a suicide attempt, compared with 23.4% with one adversity who had not made an attempt. Physical abuse (24.9%), parental divorce (14.2%) and parental death (11.6%) were most prevalent among those suicide attempters. Among those exposed to one childhood adversity, without a suicide attempt, the two

For peer review only

most prevalent adversities reported were physical abuse (12.2%) and parental death (11.3%). In the total sample 15.4% of participants exposed to two or more adversities had a suicide attempt. In contrast, 8.6% of participants exposed to two or more adversities had not made an attempt (Table 2).

Prevalence of childhood adversities among suicidal ideators in the total sample

In the sample as a whole, 35.9% of those with one adversity had suicidal ideation compared with 22 .7% of those with one adversity who had no ideation. The most prevalent adversities associated with suicidal ideation were physical abuse (21.1%), parental death (13.9%), and parental divorce (7.9%). Among those without suicidal ideation, physical abuse (11.8%) and parental death (11.3%) were the most commonly endorsed childhood adversities. Of those who endorsed two or more childhood adversities, 10.8% reported suicidal ideation and 8.6% did not (Table 2). In summary, the most prevalent childhood adversities reported among the total sample with/without suicidal ideation were firstly, physical abuse and secondly, the death of a parent.

Prevalence of suicide attempts in the total sample

In the total sample, 24.9% of those with childhood physical abuse had attempted suicide while 12.2% of respondents with no physical abuse had no attempt. Of those exposed to parental divorce, 14.2% had attempted suicide and 4.8% had made no attempt. The second most prevalent childhood adversity was parental death with 11.6% of those with parental death attempting suicide and 11.3% of those with parental death with no attempts (Table 2).

Prevalence of childhood adversities among suicidal ideators

With/without a plan

For peer review only

Association between childhood adversities and suicidality

Among suicidal ideators with a plan, 32.9% had experienced one childhood adversity. Among ideators with no plan, 41.7% had one childhood adversity. Among ideators with a plan, the following were the most prevalent childhood adversities: physical abuse (24.3%), parental death (12.2%), and parental divorce (9.7%). Among ideators without a plan, 27.9% endorsed physical abuse, 16.1% parental death, and 9.2% parental divorce (see Table 2). In both groups (ideators with and without a plan), physical abuse was the most prevalent childhood adversity, followed by parental death and parental divorce.

With or without an attempt

Among suicidal ideators who had attempted suicide, 35.4% were exposed to one childhood adversity and 15.4% were exposed to two or more childhood adversities. In the group of ideators who had made an attempt, 24.9% had experienced physical abuse, 14.2% parental divorce, and 11.6% parental death (Table 2). 40.5% of those with one adversity, and 9.6% of those exposed to two or more adversities were suicidal ideators with no attempts. In this group, the most prevalent adversities were physical abuse (24.5%), parental death (15.6%) and parental divorce (6.7%) reported (Table 2).

Among all ideators (with/without a plan, with/without an attempt), the most prevalent childhood adversity was physical abuse, followed by parental death and parental divorce. Of note, in the group of ideators with an attempted suicide parental divorce was more prevalent than parental death.

Bivariate and multivariate results: Type of childhood adversity

Bivariate and multivariate analyses were performed to examine the associations between the different childhood adversities (physical abuse, sexual abuse, parental death, parental divorce,

For peer review only

Association between childhood adversities and suicidality

other parental loss, family violence, physical illness, financial adversity) and lifetime suicidal ideation, plans and attempts.

In the total sample, bivariate and multivariate analysis revealed significant associations between (i) sexual abuse (bivariate: OR=7.9, p=0.003; multivariate: OR=7.6, p=0.003), (ii) physical abuse (OR 2, p=0.007; OR 2.0, p=0.006) and (iii) parental divorce (OR 2.8, p<.001; OR 2.7, p=0.001), and lifetime suicide attempts. Among ideators in the sample, physical abuse (OR=1.7, p<.001; OR=1.7, p<.001) was significantly associated with suicidal ideation. Multivariate analyses revealed an additional association with suicidal ideation, namely parental divorce (OR = 1.6, p=0.038). The relationship between childhood adversities and lifetime plans was not statistically significant. However, a significant association was found between parental divorce and lifetime suicidal attempts among ideators (OR=3.0, p<.001; OR=3.1, p=0.023) (Table 3).

Findings from multivariate analysis, therefore, confirm findings of bivariate analysis for all groups, except for ideators. Among ideators bivariate analysis revealed a significant relationship between physical abuse and suicidal ideation. This was confirmed in multivariate analysis where the association between parental divorce and suicidal ideation was significant for the whole sample.

Bivariate associations between the number of adversities and lifetime suicidality

The relationship between the number of childhood adversities and lifetime suicidal ideation, plans and attempts was further examined. There was a significant relationship between the number of childhood adversities and lifetime suicide attempts. Two or more childhood adversities were associated with a 2-fold higher risk of lifetime suicide attempts in the total

For peer review only

Association between childhood adversities and suicidality

sample (OR=2.1, p<.001). A significant relationship was also established between one, as well as two or more adversities with ideators in the total sample. Among ideators, no significant association was found between the number of childhood adversities and lifetime plans. A significant relationship was found between two or more adversities and lifetime attempts among ideators (OR=2.7, p=0.016), indicating a more than 2-fold higher risk of lifetime suicide attempts in this group (Table 4).

Multivariate associations between number of childhood adversities and lifetime suicidality

In the final multivariate model which included 2 or more adversities as a predictor variable, sexual abuse (OR=9.3, p<.001), childhood physical abuse (OR=2.2, p=0.003) and parental divorce (OR=3.1, p<.001) retained significant associations with lifetime suicide attempts in the total sample. Physical abuse (OR=2.1, p<.001), parental death (OR=1.7, p=0.010), parental divorce (OR=1.9, p=0.004) and other parental loss (OR = 2.1, p=0.004) were significant predictors of suicidal ideation. Physical abuse was associated with a lower odds of lifetime suicide plans among ideators (OR = 0.4, p=0.038). There were no significant associations between childhood adversities and lifetime attempts among those with suicidal ideation (Table 5).

Associations between the types of childhood adversity and lifetime suicidality over the life course

Multivariate analyses were performed to examine the association between the types of childhood adversity and lifetime suicidal ideation, plans and attempts during childhood years (age 4- 12), teenage years (age 13-19), young adulthood (age 20-29) and later adulthood (30 years and older) (Tables available from authors).

For peer review only

Association between childhood adversities and suicidality

Childhood years (4-12). Sexual abuse (OR=61.6, CI=4.5-841.0, p=0.002) in early childhood (4-12 years of age) was significantly associated with lifetime suicide attempts in the total sample (OR = 61.6, CI=4.5-841.0, p=0.002). Both sexual abuse (OR=34.8, CI= 3.1-392.6, p=0.003) and physical abuse (OR=3.7, CI=1.0-13.4, p=0.041) were associated with a higher risk for suicidal ideation among the total sample. No significant associations were found between any of the childhood adversities and lifetime plans in the group of ideators. Among those with suicidal ideation, parental death (OR=2.2, CI=1.1-4.3, p=0.021) was significantly associated with suicide attempts in childhood years.

Teen years (13-19). Sexual abuse (OR=20.3, CI=2.0-210.2, p=0.010), physical abuse (OR=3.7, CI=1.5-9.2, p=0.004), and parental divorce (OR=4.6, CI=1.7-12.1, p=0.002) were significantly associated with suicide attempts in the total sample of teenagers. Physical abuse (OR=3.6, CI=2.2-5.9, p<.001) and parental death (OR=2.2, CI=1.1-4.3, p=0.021) significantly increased the risk for suicidal ideation among the total group of teens. Physical illness (OR=9.9, CI=1.8-54.0, p=0.007) significantly increased the risk of suicidal plans in teens with suicidal ideation. Suicide attempts among teens with suicidal ideation was significantly predicted by parental divorce (OR=4.3, CI=1.1-17.0, p=0.035).

Young adulthood (20-29). None of the childhood adversities were significantly associated with lifetime suicide attempts during young adulthood in the sample overall. An explanation could be that suicide attempts spike earlier and later in life among South Africans, contributing to the lack of significance. Parental loss other than parental death was significantly associated with suicidal ideation (OR=2.9, CI=1.2-7.4, p=0.019).

For peer review only

Association between childhood adversities and suicidality

Later adulthood (≥ 30). Childhood physical abuse (OR=2.2, CI=1.0-4.8, p=0.035) was significantly predictive of suicidal attempts. The likelihood of suicidal ideation significantly increased in later adulthood if parental loss other than parental death (OR=5.1, CI=2.1-12.1, p<.001) or physical illness had been present during childhood (OR=4.3, CI=1.1-15.9, p=0.028). No significant relationship was found between any of the childhood adversities and lifetime plans in the group of ideators although a significant relationship was found between two or more adversities and lifetime plans among those who were ideators (OR=44.5, CI=2.5-779.1, p<0.008). None of the childhood adversities were significantly associated with suicide attempts among ideators in this age group.

DISCUSSION

Rates of childhood adversities and suicidal behaviours were both high among South Africans, with more than a third of respondents in the total sample who attempted suicide experiencing one childhood adversity, and 15.4% experiencing two or more adversities. Overall, physical abuse, sexual abuse, parental divorce and physical illness were far more prevalent in those with a suicide attempt than in those without. The most prevalent childhood adversities endorsed overall were physical abuse followed by parental death. Physical abuse, parental divorce and death of a parent were also the most prevalent adversities experienced in those with a suicide attempt as well as in those with suicidal ideation. These findings are somewhat dissimilar to other country samples; for example in the 21 countries that participated in the WMHS, physical abuse (29.3%), family violence (24.8%) and neglect (19.3%) were the most prevalent childhood adversities among those with a lifetime suicide attempt, while physical abuse (20.6%), family violence (17.6%) and death of a parent (14.2%) were most often reported among participants with lifetime suicidal ideation

For peer review only

Association between childhood adversities and suicidality

[20]. Cross-nationally, it would appear that physical abuse is the commonest childhood adversity associated with lifetime suicide attempts and ideation [20].

The estimate lifetime prevalence of 2.9% for attempted suicide among South Africans is close to the rates of 4.6% and 4.1% reported for general and Black populations respectively in USA. In addition the 9.1% estimated prevalence of suicide ideation is comparable with previous estimates from studies in South African clinical samples. Joe et al. (2008b) reported for the first time on the rates of suicide ideation, plan and attempts among the different ethnic groups, in data from the SASH study [6]. Overall, the results suggest that people in SA engage in suicidal thought and behaviours at levels nearly comparable with those of Western nations.

When examining suicidal behaviour risk in the context of childhood adversity, sexual abuse, physical abuse and parental divorce emerged as significant risk factors for lifetime suicide attempts in the total sample. Furthermore, physical abuse and parental divorce were significant risk factors for suicidal ideation in the total sample, while parental divorce emerged as a significant risk factor among ideators with lifetime suicide. These findings are largely consistent with the data from the overall cross-national WMHS, which found that physical and sexual abuse significantly increased the likelihood of suicidal ideation and attempts, while neglect was a risk factor for suicidal behaviour in multivariate additive analyses [20].

Of the adversities implicated, sexual and physical abuse were more significant risk factors than other adversities, highlighting the fact that intrusive and aggressive experiences in childhood may have more devastating and longer lasting effects [58]. This may be due to the extreme powerlessness and loss of control that such abuse causes, or to physically aggressive assaults resulting in the devaluation of one's body and consequent susceptibility to self harm [28]. In a

For peer review only

country with high rates of sexual and physical abuse [46] this is particularly concerning. The impact of parental divorce on suicidality supports previous findings that parental divorce, if accompanied by other adversities such as childhood abuse, increases the risk of suicidal behaviour [59].

We also found that exposure to *two or more childhood adversities* significantly increased the risk of suicide attempts among ideators. This confirms earlier work showing exposure to multiple childhood adversities increases the risk of suicidal behaviour [21, 23, 24, 60, 61]. Bruffaerts et al (2010) found a sub-additive effect with regards to the onset of suicidal behaviour when considering multiple adversities [20]. Thus, the impact of multiple adversities was not equal to the sum of the odds ratios of individual adversities. In the overall WMHS analysis exposure to multiple childhood adversities had a significant effect on the persistence of suicide when considering every additional childhood adversity exposed to, however in the current study it was not possible to stratify the number of adversities beyond two or more adversities (i.e. into more than 2 categories) given the relatively small number of cases in the sample overall with non-fatal suicidal behaviour. Physical abuse, parental death, parental loss other than through death, and parental divorce emerged as independent risk factors for suicidal ideation in the total sample. Moreover, the effects of childhood adversities on suicidal tendencies tended to differ over the *life course*. Consistent with nationally representative data in WMHS, childhood adversities were associated with the highest risk of suicide attempts in childhood, with a decrease in risk in adolescence and young adulthood, followed by an increase in risk again during later adulthood [20].

In *childhood*, sexual abuse was significantly associated with lifetime suicide attempts in the total sample, while sexual and physical abuse were significantly associated with suicidal ideation.

For peer review only - http://bmjopen.bmj.com/site/about/guidelines.xhtml

Association between childhood adversities and suicidality

Among suicidal ideators, parental death was significantly associated with lifetime suicide attempts. Exposure to childhood sexual abuse, physical abuse or parental divorce significantly increased suicide attempts during *teenage years*, while physical abuse and parental death were associated with suicidal ideation in teens. Among teen suicidal ideators, physical illness was significantly associated with suicidal plans, while parental divorce was associated with suicide attempts. These findings emphasize the need to focus suicide prevention strategies at youth in particular. In *young adulthood,* parental loss other than the death of a parent was significantly associated with suicidal ideation in the total sample. Interestingly, childhood physical abuse was identified as a significant risk factor for suicidal attempts in *later adulthood*, while childhood physical illness and parental loss other than the death of a parent significantly increased the risk for ideation.

Similar to findings from SASH, childhood sexual abuse emerged as a particularly robust risk factor for suicide attempts in younger participants in the WMH cross-national analysis, with a 10.9 times higher odds of suicide attempts in children, a 6.1 times higher likelihood in adolescents and a 2.9-fold risk in young adults who were exposed [20]. This is in keeping with Enns hypothesis that sexual abuse results in suicidal behaviour at a younger age [21]. Consistent with other studies, childhood physical and sexual abuse, in particular, emerged as risk factors for the emergence and persistence of suicidal behaviour, especially in adolescence. Loss of a parent, physical ill-health and family violence has also been found to be associated with persistence of suicidality [20, 28, 58]. These findings extend previous work done in other developing countries that have found childhood adversities to be a significant risk factor for suicidality [20, 62-64].

Limitations

For peer review only

Association between childhood adversities and suicidality

The following limitations need to be highlighted. First, recall bias might have impacted on the accuracy of recall of childhood adversities. This said, participants were asked questions about childhood adversities in sequence which may have facilitated more accurate recall [65]. Systematic reviews have also found that recall of past experiences can be accurate and can provide valuable data [66, 67]. Thus, there is evidence to support the validity of accurate recall of childhood adversities [67]. Further, studies have shown that responses to questions on childhood adversities, similar to those asked in the SASH study, generally remain stable over time [68, 69]. We recommend that future studies examine ethnicity in relation to adversity and suicidal outcomes. Second, in view of the cross-sectional design, more detailed, temporal information on childhood adversities and suicidal incidents was not obtained. Third, variables such as culture, ethnicity and mental status at the time of the interview may have influenced the recall and reporting of suicidal behaviour. It is possible that response bias may have been particularly skewed to disenfranchised South Africans (e.g. poor, young, urban an black respondents), who may have been too afraid to divulge information on suicidality. Stigma associated with mental health problems may have also played a role in the reporting suicidal tendencies. Thus, participants' mental health status, ethnicity, culture and generational factors may have also contributed to the under-reporting of suicidality. It is possible that individuals reporting childhood adversities may have also been more likely to report suicidal behaviour, while those not reporting childhood adversities may have underreported suicidality. Stigma and mental health status (e.g. depressed persons may be more inclined to report suicidality and more likely to remember negative childhood experiences) may also be contributory factors. In addition, some participants may have been afraid to report suicidal behaviours. The role of ethnicity, culture and generational factors may have also contributed to the under-reporting of suicidality. Overall, it is much more

For peer review only

likely that adversities and suicidality were under-reported rather than over-reported [9, 20, 67, 70]. Fourth, we do not assess for self-mutilating behavior. The importance of discriminating suicidal behaviour from non-suicidal self-mutilation cannot be underestimated. Fifth, the survey was conducted in adults living in households and hostel quarters thus the findings are not generalizable to homeless and institutionalized persons who were not included in the survey. Sixth, the CIDI instrument which was used in this study is a lay-administered instrument which does not include an assessment of several key DSM-IV diagnoses (such as bipolar disorder and psychosis), are associated with elevated rates of suicidality. As a result, some participants with suicidality may have not have been diagnosed with a disorder. Furthermore, in view of the large confidence intervals and small sample sizes for some of these analyses caution is required in drawing conclusions. In addition, we did not control for other unmeasured causes of childhood adversities and suicidality, or protective (resiliency) factors that may have contributed to the associations observed in these data. Both other risk and resiliency factors may have contributed to both the prevalence of non-fatal suicidal behaviours and to the associations with different forms of childhood adversity and warrant further investigation. Lastly, it is important to point out that these data were collected approximately 10 years ago. Notwithstanding these limitations, this study represents the first investigation among South Africans of a wide range of childhood adversities and their impact on the onset and persistence of suicidality over the life course.

Conclusions

Childhood adversities especially sexual abuse, physical abuse and parental divorce are associated with the onset and persistence of suicidal behaviour with the risk greatest in children and adolescents. Public health efforts aimed at prevention of early childhood sexual and physical

For peer review only

abuse, in particular, may have a significant impact on reducing suicidality over the life course and improving mental health outcomes.

For peer review only

Association between childhood adversities and suicidality

Acknowledgements

The South African Stress and Health Study was conducted in conjunction with the World Health Organization World Mental Health (WMH) Survey initiative. We thank the WMH staff for assistance with instrumentation, fieldwork and data analysis. The US National Institute of Mental Health, the John D. and Catherine T. MacArthur Foundation, the Pfizer Foundation, US Public Health Service, the Fogarty International Center, Pan American Health Organization, Eli Lilly and Company, Ortho-McNeil Pharmaceutical, GlaxoSmithKline and Bristol-Myers Squibb supported the activities of this initiative. The SASH Study was funded by grant R01-MH059575 from the National Institute of Mental Health and the National Institute of Drug Abuse.

Declarations of interest and funding

This study was funded by the NIH. Additional funding was received from the South African Department of Health and the University of Michigan. D.J.S. and S.S. are also supported by the Medical Research Council of South Africa. D.J.S. has received research grants for investigator-initiated trials and / or consultancy honoraria from AstraZeneca, Eli Lilly, GlaxoSmithKline, Lundbeck, Orion, Pfizer, Pharmacia, Roche, Servier, Solvay, Sumitomo and Wyeth. S.S. has received research grants for investigator-initiated trials and/or travel sponsorship from AstraZeneca, Eli Lilly, GlaxoSmithKline, Lundbeck, Janssen, Dr Reddy's and Servier. S.S. is supported by the South African Research Chairs Initiative of the Department of Science and Technology and the National Research Foundation. B.B has received congress sponsorship from Janssen-Cilag.

Contributorship Statement

Belinda Bruwer: Data interpretation, drafting manuscript, final approval of manuscript submitted for publication, ensuring that questions related to the accuracy of the work are appropriately

For peer review only

resolved.

Ravi Govender: Data interpretation, drafting manuscript, final approval of manuscript submitted for publication, ensuring that questions related to the accuracy of the work are appropriately resolved

Melanie Bishop: Data interpretation, revising the manuscript, final approval of manuscript submitted for publication, ensuring that questions related to the accuracy of the work are appropriately resolved

David Williams: Substantial contributions to the conception or design of the work, data acquisition, data analysis and interpretation, critically revising of the manuscript, final approval of the version to be published; and accountability for all aspects of the work

Dan Stein: Substantial contributions to the conception or design of the work, data acquisition, data analysis and interpretation, critically revising of the manuscript, final approval of the version to be published; and accountability for all aspects of the work

Soraya Seedat: Substantial contributions to the conception or design of the work, data acquisition, data analysis and interpretation, critically revising of the manuscript, final approval of the version to be published; and accountability for all aspects of the work

Data Sharing Statement

Data for the SASH Study were collected between January 2002 and June 2004. WMH surveys were carried out in 21 countries which included Nigeria and South Africa (Williams et al., 2004). For detailed information on study methods see Williams et al., 2004

All data analyses were processed and analysed centrally by a team of statisticians at the Harvard School of Public Health (Boston, USA) using the SAS version 9.1.3 software package

For peer review only

Association between childhood adversities and suicidality

REFERENCES

1. World Health Organization. Suicide Prevention (SUPRE). Geneva, Switzerland. 2007. http://www.who.int/mental_helath/prevention/suicide/suicideprevention/en/)

2. Murray, C.L., Lopez, A.D. The global burden of disease: a comprehensive assessment of mortality and disability from disease, injuries and risk factors in 1990 and projected to 2020. Cambridge, MA: Harvard University Press, 1996.

3. Burrows, S., Laflamme, L. Pattern analysis of suicide mortality surveillance data in urban South Africa. Suicide and Life- Threatening Behaviour 2008;**38**:209-220.

4. Meel, B.I. Epidemiology of suicide by hanging in Transkei. South Africa. Am J Forensic Med Pathol. 2006;**27**:75-78

5. Flisher, A.J., Liang, H., Laubscher, R. Suicide trends in South Africa, 1968-90. Scand J Public Health 2004;**32**:411-418.

6. Joe, S., Stein, DJ., Seedat, S., Herman, A., Williams, DR. non-fatal suicidal behavior among South Africans: Results from the South Africa Stress and Health Study. Social Psychiatry Epidemiology 2008;**43**(6):454–461.doi:10.1007/s00127-008-0348-7.

7. Beautrais, A.L., Joyce, P/R/. & Mulder, R.T. (1996). Risk factors for serious suicide attempts among youth aged 13 through 24 years. J Am Acad Child Adolesc Psychiatry 1996;**35**(9):1174-1182.

8. Harrison, EC, Barraclough, B. (1997). Suicide as an outcome for mental disorders: A meta-analysis. Br J Psychiatry 1997;**170**:205-228

For peer review only

9. Joe, S., Stein, D.J., Seedat, S., et al. Prevalence and correlates of non-fatal suicidal behaviour among South Africans. Br J Psychiatry 2008;**192**:310-311.
10. Nock, M.K., Borges, G., Bromet, E.J., et al. Suicide and Suicidal Behaviour. Epidemiologic Reviews 2008;**30**:133-154.
11. Nock, M.K., Borges, G., Bromet, E.J., et al. (2008b). Cross-national prevalence and risk factors for suicidal ideation, plans and attempts. *British Journal of Psychiatry*, 192, 98-105.
12. Nock, M.K., Hwang, I., Sampson, N.A., et al. Cross-national analysis of the associations among mental disorders and suicidal behaviour: Findings from the WHO World Mental Health Surveys. PLos Medicine 2009;**6**(8).e1000123.
13. Bondy, B., Buettner, A., Zill, P. Genetics of suicide. Molecular Psychiatry 2006;**11**:336-351.
14. Kohli, M.A., Salyakina, D., Pfennig, A., et al. Association of genetic variants in the neurotrophic receptor encoding gene NTRK2 and a lifetime history of suicide attempts in depressed patients. Arch Gen Psychiatry 2010;**67**:348-59.
15. Roy, A., Hu, X-Z., Janal, M.N., & Goldman, D. Interaction between childhood trauma and serotonin transporter gene variation and suicide. Neuropsychopharmacology 2007;**32**:2046–2052
16. Risch, N., Herrell, R., Lehner, T., et al. Interaction between the serotonin transporter gene (5-HTTLPR), stressful life events, and the risk of depression: A meta-analysis. JAMA 2009;**301**:2462–2471.
17. Borges, G., Benjet, C., Medina-Mora, M.E., et al. Traumatic events and suicide related outcomes among Mexico City adolescents. J Child Psychol Psychiatry 2008;**6**:654-666.Weissman MM, Bland

For peer review only

RC, Canino GJ, Greenwald S, Hwu HG, Joyce PR, et al. (1999) Prevalence of suicide ideation and suicide attempts in nine countries. *Psychology Med, 29*: 9–17.

18. Brodsky, BS & Stanley, B. Adverse childhood experiences and suicidal behaviour. Psychiatry Clinical Northern America 2008;**31**:223-235

19. Bruffaerts, R., Demyttenaere, K., Borges, G., et al. Childhood adversities as risk factors for onset and persistence of suicidal beahviour. Br J Psychiatry 2010;**197**:20-27.

20. Enns, M.W., Cox, B.J., Afifi, T.O., et al. Childhood adversities and risk for suicidal ideation and attempts: a longitudinal population-based study. Psychological Medicine 2006;**36**:1769-1778.

21. Johnson, J.G., Cohen, P., Gould, M.S., et al. Childhood adversities, interpersonal difficulties, and risk for suicide attempts during late adolescence and early adulthood. Arch Gen Psychiatry 2002;**59**:741-749.

22. Dube, S.R., Anda, R.F., Felitti, V.J., et al. Childhood abuse, household dysfunction, and the risk of attempted suicide throughout the life span: Findings from the Adverse Childhood Experiences Study. JAMA 2001;**286**:3089-3096.

23. Afifi, T.O., Enns, M.W., Cox, B.J., et al. Population attributable fractions of psychiatric disorders and suicide ideation and attempts associated with adverse childhood experiences. Am J Public Health 2008;**98**:946-952.

24. Burke, A.K., Galfalvy, H., Everett, B., et al. Effect of exposure to suicidal behavior on suicide attempt in a high-risk sample of offspring of depressed parents. J Am Acad Child Adolesc Psychiatry 2010;**49**:114-121.

For peer review only

25. Labonte, B., Suderman, M., Maussion, G.,et al. Genome-wide epigenetic regulation by early-life trauma. Arch Gen Psychiatry 2012;**69**(7):722-731.Doi:10.1001/archgenpsychiatry.2011.2287

26. Lipschitz, D.S., Winegar, R.K., Nicolaou, A.L., et al. (1999). Perceived abuse and neglect as risk factors for suicidal behaviour in adolescent inpatients. *The Journal of Nervous and Mental Disease*, 187, 32-39.

27. Ystgaard, M., Hestetun, I., Loeb, M., et. al Is there a specific relationship between childhood sexual and physical abuse and repeated suicidal behaviour? Child Abuse Neg 2004;**28**:863-875

28. Boudewyn, A., & Liem, J. Childhood sexual abuse as a presecutor to depression and self-destructive behavior in adulthood. J Trauma Stress 1995;**8**:445-459.

29. Brown, J., Cohen, P., Johnson, J.G., & Smailes, E.M. Childhood abuse and neglect: Specificity of effects on adolescent and young adult depression and suicidality. J Am Acad Child Adolesc Psychiatry 1999;**38**:1490-1496.

30. Bryant, S.L., & Range, L.M. Suicidality in college women who were sexually and physically abused and physically punished by parents. Violence Vict 1995;**10**:195-201.

31. Davidson, J.R.T., Hughes, D.C., George, L.K., & Blazer, D.G. The association of sexual assault and attempted suicide within the community. Arch Gen Psychiatry 1996;**53**:550-555

32. Fergusson, D.M., & Mullen, P.E. Childhood Sexual abuse – An evidence based perspective. Sage, CA: Thousand Oaks, 1999.

33. Finkelhor, D. Early and long-term effects of child sexual abuse: An update. Professional Psychology: Research & Practice 1990;**21**(5):325-330.

For peer review only

34. Finkelhor, D., & Hashima, P.Y. (2001). The victimization of children and youth: A comprehensive overview. In S.O. White (Ed.) Handbook of youth and justice. The Plenum series in crime and justice. Dordrecht: Plenum, 2001:49-78.

35. Holmes, W.C., & Slap, G.B. Sexual abuse of boys: Definition, prevalence, correlates, sequelae, and management. JAMA: JAMA 1998;**280**(21):1855-1862

36. Kendall-Tackett, K.A., Williams, L.M., & Finkelhor, D. Impact of sexual abuse on children: A review and synthesis of recent empirical studies. Psychol Bull 1993;**113**(1):164-180.

37. Martin, G. Reported family dynamics, sexual abuse, and suicidal behaviors in community adolescents. Arch Suicide Res 1996;**2**:183-195.

38. Peters, D.K., & Range, L.M. Childhood sexual abuse and current suicidality in college women and men. Child Abuse Negl 1995;**19**:335-341.

39. Putman, F.W. Ten-year research update review: Child sexual abuse. J Am Acad Child Adolesc Psychiatry 2003;**42**(3):269-278

40. Stepakoff, S. Effects of sexual victimization on suicidal ideation and behaviour in US college women. Suicide and Life-Threatening Behavior 1998;**28**:107-126.

41. Malinosky-Rummel, R., & Hansen, D.J. Long-term consequences of childhood physical abuse. Psychol Bull 1993;**144**:68-79

42. Silverman, A.B., Reinherz, H., & Giaconia, R.M. The long-term sequelae of child and adolescent abuse: A longitudinal community study. Child Abuse Negl 1996;**20**:709-723

For peer review only

43. Chapman, D.P., Whitfield, C.L., Felitti, V.J., et al. Adverse childhood experiences and the risk of depression in adulthood. J Affect Disord 2004;**82**:217-225

44. Dube, S.R., Felitti, V.J., Dong, M., et al. Childhood abuse, neglect, and household dysfunction and the risk of illicit drug use: The adverse childhood experiences study. Pediatricc 2003;**111**:564-572.

45. Jewkes, R.K., Dunkle, K., Nduna, M., et al. Associations between childhood adversity and depression, substance abuse and HIV and HSV2 incident infections in rural South African youth. Child Abuse Negl 2010;**34**:833-841.

46. Seedat, S., Stein, D.J., Jackson, P.B., et al. Life stress and mental disorders in the South African Stress and Health study. South African Medical Journal 2009a;**99:**375-382.

47. Williams, D.R., Herman, A., Kessler, R.C., et al. The South Africa Stress and Health Study: Rationale and Design. Metab Brain Dis 2004;**19**(1/2):135-147.

48. Statistics South Africa. Census 2001: Census in Brief. Pretoria: Statistics South Africa. 2001. Available from http://www.statssa.gov.za/census01/html/CInBrief/CIB2001.pdf (Accessed January 2014)

49. Kessler, R.C., Üstün, T.B. The World Mental Health (WMH) Survey Initiative Version of the World Health Organization (WHO) Composite International Diagnostic Interview (CIDI). Int J Methods Psychiatr Res 2004;**13**:61-98.

50. Seedat, S., Stein, D.J., Herman, A., et al. Twelve-month treatment of Psychiatric disorders in South Africa Stress and Health Study (World Mental Health Survey Initiative). Psychiatric Epidemiology 2008;**38**:211-220.

51. Seedat, S., Williams, D.R., Herman, A., et al. Mental health service use among South Africans for mood, anxiety and substance use disorders. South African Medical Journal 2009b;**99**:346-352.

For peer review only

Association between childhood adversities and suicidality

52. World Health Organization. World Health Organization Manual of the international statistical classification of diseases, injuries and causes of death, ninth revision. Geneva, Switzerland, 1992.

53. American Psychiatric Association. Diagnostic and statistical manual of mental disorders (DSM-IV), 4th Edition. Washington: American Psychiatric Association Press, 1994.

54. Straus MA. Measuring Intrafamily Conflict and Violence: The Conflict Tactics (CT) Scales. Journal of Marriage and Family 1979;**41(1**):75

55. Kessler, R.C., McLaughlin, K.A., Green, J.G. Childhood adversities and adult psychopathology in the WHO World Mental Health Surveys. Br J Psychiatry 2010;**197**:378-385.

56. Stein, D.J., Chiu, W.T., Hwang, I., et al. Cross-national analysis of the associations between traumatic events and suicidal behavior: Findings from the WHO World Mental Health Surveys. PloS ONE 2010;**5**(5):e10574.

57. Joiner Jr, T.E., Sachs-Ericsson, N.J., Wingate, L.R. Childhood physical and sexual abuse and lifetime number of suicide attempts: A persistent and theoretically important relationship. Behav Res Ther 2007;**45**:539-547.

58. Afifi, T.O., Boman, J., Fleisher, W., et al. The relationship between child abuse, parental divorce, and lifetime mental disorders and suicidality in a nationally representative adult sample. Child Abuse Negl 2009;**33**:139–147.

59. Bebbington, P.E., Cooper, C.C., Minot, S., et al. Suicide attempts, gender, and sexual abuse: data from the 2000 British Psychiatric Morbidity Survey. Am J Psychiatry 2009;**166**:1135-1140.

60. Molner, B, Buka, S, & Kessler, R. Child sexual abuse and subsequent psychopathology: results from the National Comorbidity Survey. American Journal Public Health 2001;**91**:753-760.

For peer review only

61. Borges, G., Angst, J., Nock, M.K., et al. Risk factors for the incidence and persistence of suicide related outcomes: a 10 year follow up study using the National Comorbidity Surveys. J Affect Disord 2008;**105**:25-33

62. Xing, X-Y., Tao, F-B., Wan, Y-H., et al. Family factors associated with suicide attempts among Chinese adolescent students: A national cross-sectional survey. J Adolesc Health 2010;**46**:592-599.

63. Gureje, O., Kola, L., Uwakwe, R., et al. The profile and risks of suicidal behaviours in the Nigerian Survey of Mental Health and Well Being. Psychol Med 2007;**37**:821-830.

64. Knauper, BC., CF, Schwarz, N., Bruce, ML., et al. Improving the accuracy of major depression age of onset reports in the US National Comorbidity Survey. Int J Methods Psychiatr Res 1999;**8**(1):39-48

65. Brewin, CR., Andrews, B., Botlib, IH. Psychopathology and early experience: a reappraisal of retrospective reports. Psychol Bull 1993;**113**:82-98

66. Hardt, J., Rutter, M. Validity of adult retrospective reports of adverse childhood experiences: a review of the evidence. J Child Psychol Psychiatry 2004;**45**:260-273.

67. Dube, SR., Williamson, DF., Thompson, T., et al. Assessing the reliability of retrospective reports of adverse childhood experiences amond adult HMO members attending a primary care clinic. Child Abuse Negl 2004;**28**(7):729-737.

68. Yancura, LA., Aldwin, CM. (2009). Stability and change in retrospective reports of childhood experiences over a 5-year period: Findings from the David Longitudinal Study. Psychol Aging 2009;**24**(3):715-721

For peer review only

69. Wilsnack, S.C., Wonderlich, S.A., Kristjanson, A.F., et al. (2002). Self reports of forgetting and remembering childhood sexual abuse in a nationally representative sample of US women. Child Abuse Negl 2002;**26**:139-147.

For peer review only

Association between childhood adversities and suicidality

Table 1: Descriptive Characteristics (N= 4351)

		N
Mean Age (yrs) (SE)	37.0 (0.26)	
**Age categories (yrs)**		
18 – 29	39.1%	1701
30 – 39	22.1%	962
40 - 49	18.1%	788
≥ 50	20.7%	901
**Sex**		
Male	46.3%	2015
Female	53.7%	2336
**Race**		
Black	76.2%	3315
Coloured	10.4%	453
White	10.0%	435
Indian/Asian	3.4%	148
**Married**	50.1%	2180
**Location**		
Rural	38.4%	1671
Urban	61.6%	2680
**Education**		
None	6.8 %	296
Grade 1-7	19.1%	831
Grade 8-11	35.4%	1540
Matric	23.5%	1022
Matric +	15.3%	666
**Employed**	31.0%	1349
**Income Category** (Rands), (mean SD)		
0	13.7%	596
1 - 2500	29.5%	1284
2501 – 5000	15.4%	670
5001 – 10 000	19.6%	853
≥ 10001	21.8%	949
**Province**		
Eastern Cape	13.1%	570
Free State	6.2%	270
Guateng	23.0%	1001
Kwazulu Natal	19.5%	848
Limpopo	10.5%	457
Mpumalanga	6.6%	287
Northern Cape	1.9%	83
North West	8.3%	361
Western Cape	11.1%	483

For peer review only

Association between childhood adversities and suicidality

Table 2: Prevalence of childhood adversities and suicidal behaviour in South Africa

[%[b] (S.E.)]

	Total Sample		Total Sample		Suicidal Ideators		Suicidal Ideators	
	With Attempt	No attempt	With Ideation	No ideation	With Plan	No plan	With Attempt	No attempt
Physical Abuse	24.9 (4.6)	12.2 (0.8)	21.1 (2.5)	11.8 (0.7)	24.3 (4.6)	27.9 (4.0)	24.9 (4.6)	24.5 (3.6)
Sexual Abuse	2.1 (1.2)	0.1 (0.0)	0.7 (0.4)	0.1 (0.0)	1.6 (0.9)	0.0 (0.0)	2.1 (1.2)	0.0 (0.0)
Parent Died	11.6 (2.4)	11.3 (0.6)	13.9 (2.3)	11.3 (0.6)	12.2 (2.4)	16.1 (4.2)	11.6 (2.4)	15.6 (3.8)
Parent Divorced	14.2 (3.8)	4.8 (0.4)	7.9 (1.6)	4.7 (0.4)	9.7 (2.6)	9.2 (3.7)	14.2 (3.8)	6.7 (2.9)
Other Parent Loss	2.1 (1.2)	2.2 (0.4)	3.9 (1.2)	2.1 (0.4)	1.1 (0.6)	3.0 (1.4)	2.1 (1.2)	2.7 (1.3)
Family Violence	4.3 (1.5)	3.0 (0.3)	4.1 (0.9)	2.9 (0.3)	4.7 (1.5)	6.3 (1.8)	4.3 (1.5)	4.5 (1.4)
Physical Illness	5.0 (2.3)	2.5 (0.3)	4.0 (1.2)	2.4 (0.3)	4.4 (1.8)	4.7 (1.8)	5.0 (2.3)	4.3 (1.6)
Financial Adversity	6.1 (2.4)	5.6 (0.5)	4.1 (0.9)	5.8 (0.5)	6.0 (2.1)	3.3 (1.5)	6.1 (2.4)	2.9 (1.0)
1	35.4 (4.2)	23.4 (1.0)	35.9 (2.8)	22.7 (0.9)	32.9 (4.0)	41.7 (5.2)	35.4 (4.2)	40.5 (4.5)
2+	15.4 (3.4)	8.6 (0.5)	10.8 (1.7)	8.6 (0.5)	14.1 (3.2)	13.2 (3.3)	15.4 (3.4)	9.6 (2.3)
a	(140)	(107309)	(394)	(112243)	(171)	(1976)	(140)	(2212)

[a] Number of cases with the outcome variable; N represents the number of person years.
[b] % represents the percentage of people with the adversity among the cases with the outcome variable indicated in the column header. For example: the first cell is the % of those with physical abuse among those with attempts.

For peer review only

Association between childhood adversities and suicidality

Table 3: Multivariate and Bivariate models for associations between childhood adversities and lifetime suicidality[1]

	LT Attempts in total sample[b]				Ideators among total sample[c]				Suicidal Ideators with LT plans [d]				Suicidal Ideators with LT attempts [e]			
	Multivariate		Bivariate		Multivariate		Bivariate		Multivariate		Bivariate		Multivariate		Bivariate	
	OR(95% CI)	Chi-square	OR(95% CI)	Chi-square	OR(95% CI)	Chi-square	OR(95% CI)	Chi-square	OR(95% CI)	Chi-square	OR(95% CI)	Chi-square	OR(95% CI)	Chi-square	OR(95% CI)	Chi-square
Physical Abuse	2.0* (1.2-3.3)*	7.4(0.006)*	2.0* (1.2-3.2)*	7.3(0.007)*	1.7* (1.3-2.3)*	15.2(<.001)*	1.7* (1.3-2.3)*	16.7(<.001)*	0.6 (0.3-1.4)	1.3(0.25)	0.7 (0.3-1.4)	1.2(0.26)	1.0 (0.5-2.3)	0.0(0.93)	1.1 (0.5-2.5)	0.1(0.81)
Sexual Abuse	7.6* (2.0-29.9)*	8.9(0.003)*	7.9* (1.9-32.1)*	8.6(0.003)*	2.6 (0.6-10.6)	1.8(0.18)	3.0 (0.7-12.2)	2.5(0.11)	---	---	---	---	---	---	---	---
Parent Died	1.1 (0.6-1.8)	0.1(0.78)	1.1 (0.7-1.7)	0.1(0.76)	1.4 (0.9-2.1)	2.7(0.10)	1.3 (0.9-1.9)	2.0(0.16)	0.7 (0.3-1.7)	0.6(0.45)	0.8 (0.4-1.9)	0.3(0.62)	0.8 (0.5-1.5)	0.4(0.52)	0.8 (0.4-1.5)	0.4(0.53)
Parent Divorced	2.7* (1.5-5.0)*	10.8(0.001)*	2.8* (1.5-5.2)*	11.4(<.001)*	1.6* (1.0-2.4)*	4.3(0.038)*	1.5 (1.0-2.3)	3.7(0.05)	0.9 (0.3-3.3)	0.0(0.88)	1.2 (0.4-3.8)	0.1(0.78)	3.1* (1.2-8.6)*	5.2(0.023)*	3.0* (1.1-8.0)*	4.9(0.027)*
Other Parent Loss	1.0 (0.3-3.3)	0.0(0.95)	0.9 (0.3-2.8)	0.1(0.81)	1.7 (1.0-3.0)	3.6(0.06)	1.6 (0.9-2.7)	2.9(0.09)	0.4 (0.1-2.6)	0.9(0.34)	0.5 (0.1-2.7)	0.7(0.41)	2.0 (0.2-17.3)	0.4(0.51)	2.5 (0.6-11.0)	1.5(0.22)
Family Violence	0.7 (0.3-1.7)	0.6(0.42)	1.0 (0.4-2.2)	0.0(0.98)	0.8 (0.5-1.4)	0.5(0.47)	1.1 (0.6-1.8)	0.0(0.83)	1.0 (0.4-2.4)	0.0(0.97)	0.8 (0.4-2.0)	0.2(0.68)	2.4 (0.9-6.3)	3.5(0.06)	2.2 (0.9-5.5)	2.9(0.09)
Physical Illness	1.1 (0.4-3.5)	0.1(0.81)	1.5 (0.6-4.1)	0.7(0.39)	1.2 (0.6-2.3)	0.2(0.63)	1.3 (0.7-2.4)	0.7(0.42)	0.8 (0.2-3.1)	0.1(0.71)	0.9 (0.2-3.5)	0.0(0.86)	1.2 (0.3-3.9)	0.1(0.80)	1.2 (0.4-4.0)	0.1(0.77)
Financial Adversity	1.0 (0.4-2.7)	0.0(0.94)	1.2 (0.5-2.8)	0.1(0.73)	0.6 (0.4-1.1)	3.0(0.08)	0.7 (0.4-1.2)	1.4(0.23)	2.4 (0.7-8.4)	1.9(0.17)	1.9 (0.6-6.8)	1.1(0.29)	2.1 (0.7-6.0)	2.1(0.15)	2.0 (0.7-6.3)	1.6(0.21)

*Significant at the .05 level, two-sided test
LT: lifetime

[1] Assessed in Part 2 sample due to having part 2 controls. Controls for the model include int (1-5 intervals), and also include significant variables from demographic and parent psychopathology models, details in the following footnotes.

[b] Models controls for int(1-5 intervals), demographics (sex, age, time-varying education), interaction between intervals(13-19,20-29,30+) and age, education. For parent psychopathology, controlling for number of parental disorders (dummies for 1, 2+ disorders).

[c] Models controls for int(1-5 intervals), demographics (sex, age, time-varying education), interaction between int intervals(13-19,20-29,30+) and age, education. For parent psychopathology, controlling for number of parental disorders (dummies for 1, 2+ disorders).

[d] Models controls for int(1-5 intervals), demographics (sex, age, time-varying education), interaction between int intervals(13-19,20-29,30+) and age, education. For parent psychopathology, controlling for types of parental disorders (6 dummies).

[e] Models controls for int(1-5 intervals), demographics (sex, age, time-varying education), interaction between int intervals(13-19,20-29,30+) and age, education. Parent psychopathology not controlled for due to insignificance in previous models.

Table 4: Associations between number of childhood adversities and lifetime suicidality[1]

	LT Attempts in total sample[b]		Ideators among total sample[c]		Ideators with LT plans[d]		Ideators with LT attempts[e]	
Number of child adversities	OR(95% CI)	Chi-square	OR(95% CI)	Chi-square	OR(95% CI)	Chi-square	OR(95% CI)	Chi-square
1	1.9* (1.3-2.8)*		1.8* (1.5-2.3)*		0.5 (0.3-1.0)		0.9 (0.5-1.7)	
2+	2.1* (1.2-3.8)*	14.3(<.001)*	1.4* (1.0-2.0)*	28.3(<.001)*	1.1 (0.3-3.3)	4.5(0.10)	2.7* (1.3-5.9)*	8.3(0.016)*

*Significant at the .05 level, two-sided test
LT: lifetime

[1] Assessed in Part 2 sample due to having part 2 controls. Controls for the model include int (1-5 intervals), and also include significant variables from demographic and parent psychopathology models, details in the following footnotes.

[b]Models controls for int(1-5 intervals), demographics (sex, age, time-varying education), interaction between int intervals(13-19,20-29,30+) and age, education. For parent psychopathology, controlling for number of parental disorders (dummies for 1, 2+ disorders).

[c]Models controls for int(1-5 intervals), demographics (sex, age, time-varying education), interaction between int intervals(13-19,20-29,30+) and age, education. For parent psychopathology, controlling for number of parental disorders (dummies for 1, 2+ disorders).

[d]Models controls for int(1-5 intervals), demographics (sex, age, time-varying education), interaction between int intervals(13-19,20-29,30+) and age, education. For parent psychopathology, controlling for types of parental disorders (6 dummies).

[e]Models controls for int(1-5 intervals), demographics (sex, age, time-varying education), interaction between int intervals(13-19,20-29,30+) and age, education. Parent psychopathology not controlled for due to insignificance in previous models.

For peer review only

Association between childhood adversities and suicidality

Table 5: Final multivariate model for associations between childhood adversities and lifetime suicidality[1]

	LT Attempts in total sample[b]		Ideators among total sample[c]		Ideators with LT plans [d]		Ideators with LT attempts [e]	
	OR(95% CI)	Chi-square	OR(95% CI)	Chi-square	OR(95% CI)	Chi-square	OR(95% CI)	Chi-square
Physical Abuse	2.2* (1.3-3.8)*	8.9(0.003)*	2.1* (1.6-2.8)*	25.4(<.001)*	0.4* (0.2-1.0)*	4.3(0.038)*	0.8 (0.3-2.1)	0.3(0.60)
Sexual Abuse	9.3* (2.5-35.2)*	11.2(<.001)*	3.7 (0.9-15.9)	3.3(0.07)	---	---	---	---
Parent Died	1.2 (0.7-2.3)	0.4(0.51)	1.7* (1.1-2.6)*	6.6(0.010)*	0.4 (0.1-1.3)	2.2(0.14)	0.6 (0.3-1.1)	2.8(0.10)
Parent Divorced	3.1* (1.7-5.6)*	14.5(<.001)*	1.9* (1.2-3.0)*	8.1(0.004)*	0.7 (0.2-2.3)	0.4(0.51)	2.4 (0.9-6.4)	3.0(0.08)
Other Parent Loss	1.1 (0.3-4.3)	0.0(0.87)	2.1* (1.3-3.6)*	8.3(0.004)*	0.3 (0.0-2.0)	1.8(0.18)	1.3 (0.1-13.3)	0.1(0.79)
Family Violence	0.9 (0.3-2.3)	0.1(0.76)	1.1 (0.6-2.3)	0.2(0.69)	0.4 (0.1-1.8)	1.6(0.20)	1.2 (0.4-4.1)	0.1(0.76)
Physical Illness	1.4 (0.4-5.3)	0.2(0.63)	1.6 (0.7-3.3)	1.4(0.24)	0.6 (0.1-2.5)	0.5(0.46)	0.9 (0.2-3.3)	0.0(0.85)
Financial Adversity	1.3 (0.4-3.7)	0.2(0.65)	0.9 (0.4-1.7)	0.1(0.71)	1.6 (0.4-6.0)	0.6(0.44)	1.4 (0.5-4.3)	0.4(0.52)
group significance test for all types		29.4(<.001)*		43.0(<.001)*		833.9(<.001)*		11.5(0.18)
significance test for difference between types		13.1(0.07)		9.2(0.24)		805.7(<.001)*		11.8(0.11)
2+ adversities	0.7 (0.2-1.8)	0.7(0.41)	0.5* (0.3-0.9)*	4.9(0.028)*	4.7 (0.8-29.2)	2.9(0.09)	2.9 (0.8-10.6)	2.7(0.10)

*Significant at the .05 level, two-sided test
LT: lifetime

[1] Assessed in Part 2 sample due to having part 2 controls. Controls for the model include int (1-5 intervals), and also include significant variables from demographic and parent psychopathology models, details in the following footnotes.

[b]Models controls for int(1-5 intervals), demographics (sex, age, time-varying education), interaction between int intervals(13-19,20-29,30+) and age, education. For parent psychopathology, controlling for number of parental disorders (dummies for 1, 2+ disorders).

[c]Models controls for int(1-5 intervals), demographics (sex, age, time-varying education), interaction between int intervals(13-19,20-29,30+) and age, education. For parent psychopathology, controlling for number of parental disorders (dummies for 1, 2+ disorders).

[d]Models controls for int(1-5 intervals), demographics (sex, age, time-varying education), interaction between int intervals(13-19,20-29,30+) and age, education. For parent psychopathology, controlling for types of parental disorders (6 dummies).

[e]Models controls for int(1-5 intervals), demographics (sex, age, time-varying education), interaction between int intervals(13-19,20-29,30+) and age, education. Parent psychopathology not controlled for due to insignificance in previous models.

For peer review only - http://bmjopen.bmj.com/site/about/guidelines.xhtml

For peer review

South Africa Table 6a. Multivariate model for associations between child adversity and LT suicidality[1]

For peer review only - http://bmjopen.bmj.com/site/about/guidelines.xhtml

Association between childhood adversities and suicidality

	Life stage	Int range 4-12							
		LT Attempts in total sample[2]		Ideators among total sample[3]		Among Ideators, LT Plans[4]		Among Ideators, LT Attempts[5]	
		OR(95% CI)	Chisquare	OR(95% CI)	Chisquare	OR(95% CI)	Chisquare	OR(95% CI)	Chisquare
Physical Abuse	Childhood	0.6 (0.1-4.3)	0.3(0.60)	**3.7* (1.0-13.4)***	**4.2(0.041)***	---	---	**0.0* (0.0-0.0)***	**1177.3(<.001)***
	Teen years	**3.7* (1.5-9.2)***	**8.5(0.004)***	**3.6* (2.2-5.9)***	**26.1(<.001)***	0.3 (0.0-2.5)	1.2(0.28)	1.1 (0.3-4.7)	0.0(0.90)
	Young adult	1.6 (0.8-3.5)	1.7(0.20)	1.1 (0.7-1.8)	0.2(0.64)	0.4 (0.1-1.2)	2.7(0.10)	0.9 (0.3-3.2)	0.0(0.88)
	Later adult	**2.2* (1.0-4.8)***	**4.4(0.035)***	1.8 (0.9-3.5)	3.2(0.07)	0.4 (0.1-1.2)	2.8(0.10)	1.4 (0.4-5.3)	0.2(0.65)
Sexual Abuse	Childhood	**61.6* (4.5-841.0)***	**9.9(0.002)***	**34.8* (3.1-392.6)***	**8.6(0.003)***	---	---	---	---
	Teen years	**20.3* (2.0-210.2)***	**6.6(0.010)***	4.6 (0.3-61.6)	1.4(0.24)	---	---	---	---
	Young adult	5.1 (0.4-66.1)	1.6(0.20)	2.2 (0.3-17.5)	0.5(0.46)	---	---	**0.0* (0.0-0.0)***	**68.1(<.001)***
	Later adult	**0.0* (0.0-0.0)***	**81.7(<.001)***	**0.0* (0.0-0.0)***	**218.1(<.001)***	---	---	0.8 (0.0-16.7)	0.0(0.88)
Parent Died	Childhood	2.6 (0.1-52.0)	0.4(0.53)	1.5 (0.1-16.9)	0.1(0.76)	8.6 (0.3-234.7)	1.7(0.19)	**22.7* (1.5-338.3)***	**5.3(0.021)***
	Teen years	1.8 (0.5-6.5)	0.9(0.35)	**2.2* (1.1-4.3)***	**5.3(0.021)***	0.7 (0.1-6.8)	0.1(0.72)	0.5 (0.1-2.2)	0.8(0.38)
	Young adult	0.6 (0.2-2.4)	0.4(0.51)	1.4 (0.7-2.8)	0.7(0.40)	0.4 (0.0-2.8)	1.0(0.32)	0.6 (0.2-2.2)	0.7(0.42)
	Later adult	1.3 (0.3-4.9)	0.1(0.70)	1.6 (0.7-3.6)	1.5(0.22)	0.2 (0.0-1.6)	2.3(0.13)	0.6 (0.2-1.9)	0.8(0.37)
Parent Divorced	Childhood	3.0 (0.2-38.0)	0.7(0.39)	2.9 (0.3-24.8)	0.9(0.33)	4.6 (0.1-215.6)	0.6(0.43)	**0.0* (0.0-0.0)***	**135.3(<.001)***
	Teen years	**4.6* (1.7-12.1)***	**9.8(0.002)***	2.5 (1.0-6.1)	3.8(0.05)	0.4 (0.1-3.1)	0.8(0.37)	**4.3* (1.1-17.0)***	**4.5(0.035)***
	Young adult	1.7 (0.7-4.5)	1.3(0.25)	1.1 (0.5-2.6)	0.1(0.74)	0.8 (0.2-4.2)	0.0(0.84)	2.9 (0.7-12.7)	2.2(0.14)
	Later adult	**4.6* (1.0-21.6)***	**3.9(0.049)***	2.4 (0.9-6.2)	3.5(0.06)	0.3 (0.0-2.5)	1.2(0.28)	1.9 (0.1-31.6)	0.2(0.65)

For peer review only - http://bmjopen.bmj.com/site/about/guidelines.xhtml

Association between childhood adversities and suicidality

Other Parent Loss	Childhood	**0.0* (0.0-0.0)***	**53.4(<.001)***	**0.0* (0.0-0.0)***	**77.1(<.001)***	---	---	---	---
	Teen years	**0.0* (0.0-0.0)***	**238.2(<.001)***	0.2 (0.0-1.5)	2.6(0.11)	---	---	**0.0* (0.0-0.0)***	**60.3(<.001)***
	Young adult	1.2 (0.2-7.6)	0.1(0.82)	**2.9* (1.2-7.4)***	**5.5(0.019)***	0.1 (0.0-1.5)	3.0(0.08)	10.5 (0.7-160.1)	3.0(0.09)
	Later adult	1.3 (0.2-9.4)	0.1(0.80)	**5.1* (2.1-12.1)***	**14.1(<.001)***	0.2 (0.0-4.0)	1.1(0.30)	0.6 (0.1-6.9)	0.2(0.70)
Family Violence	Childhood	**0.0* (0.0-0.1)***	**12.9(<.001)***	1.9 (0.3-13.1)	0.5(0.48)	---	---	---	---
	Teen years	1.9 (0.5-7.2)	1.0(0.33)	2.1 (0.6-7.6)	1.5(0.23)	2.3 (0.1-46.2)	0.3(0.59)	0.9 (0.1-5.7)	
	Young adult	0.4 (0.1-1.5)	2.0(0.16)	0.5 (0.2-1.8)	1.1(0.30)	0.3 (0.0-2.6)	1.4(0.24)	2.1 (0.2-25.7)	0.4(0.55)
	Later adult	1.0 (0.2-6.5)	0.0(0.96)	0.9 (0.2-3.4)	0.0(0.86)	**0.0* (0.0-0.9)***	**4.3(0.037)***	0.8 (0.0-25.2)	0.0(0.92)
Physical Illness	Childhood	**0.0* (0.0-0.0)***	**44.3(<.001)***	1.4 (0.2-13.2)	0.1(0.75)	---	---	---	---
	Teen years	2.9 (0.3-27.8)	0.9(0.34)	1.5 (0.4-5.4)	0.3(0.56)	**9.9* (1.8-54.0)***	**7.3(0.007)***	1.5 (0.2-11.6)	0.1(0.71)
	Young adult	0.3 (0.0-5.1)	0.8(0.36)	1.0 (0.4-2.6)	0.0(0.96)	0.2 (0.0-4.6)	1.0(0.32)	0.1 (0.0-1.4)	3.0(0.08)
	Later adult	5.5 (0.9-32.1)	3.7(0.05)	**4.3* (1.1-15.9)***	**4.8(0.028)***	**0.0* (0.0-0.9)***	**4.1(0.042)***	1.6 (0.1-20.8)	0.1(0.73)
Financial Adversity	Childhood	**0.0* (0.0-0.0)***	**64.2(<.001)***	2.0 (0.2-22.3)	0.3(0.57)	**0.0* (0.0-0.0)***	**26.8(<.001)***	**0.0* (0.0-0.2)***	**10.0(0.002)***
	Teen years	1.9 (0.2-14.5)	0.4(0.53)	0.6 (0.2-2.3)	0.6(0.45)	1.0 (0.1-19.3)	0.0(1.00)	4.0 (0.4-42.9)	
	Young adult	0.8 (0.2-3.6)	0.1(0.76)	0.5 (0.2-1.4)	1.9(0.17)	1.7 (0.3-10.9)	0.3(0.57)	1.3 (0.2-7.7)	0.1(0.78)
	Later adult	2.1 (0.3-15.5)	0.6(0.44)	2.0 (0.5-8.4)	1.0(0.31)	0.7 (0.1-4.9)	0.1(0.75)	0.8 (0.1-4.6)	0.1(0.78)
group significance test for all types	Childhood		**347.6(<.001)***		**822.4(<.001)***		**204.6(<.001)***		**1425.4(<.001)***
	Teen years		**1168.3(<.001)***		**37.5(<.001)***		**421.4(<.001)***		**1337.0(<.001)***

For peer review only - http://bmjopen.bmj.com/site/about/guidelines.xhtml

Association between childhood adversities and suicidality

	Young adult		9.9(0.27)		9.6(0.30)		**1038.1(<.001)***		**97.5(<.001)***
	Later adult		**338.1(<.001)***		**525.7(<.001)***		7.9(0.34)		5.9(0.66)
significance test for difference between types	Childhood		**301.9(<.001)***		**637.3(<.001)***		**203.1(<.001)***		**1123.3(<.001)***
	Teen years		**1004.7(<.001)***		12.4(0.09)		**374.6(<.001)***		**1283.5(<.001)***
	Young adult		5.2(0.64)		10.2(0.18)		**973.6(<.001)***		**99.6(<.001)***
	Later adult		**272.0(<.001)***		**477.0(<.001)***		4.8(0.57)		5.7(0.57)
2+ adversities	Childhood	0.6 (0.0-13.1)	0.1(0.73)	0.1 (0.0-1.3)	3.2(0.07)	---	---	---	---
	Teen years	0.2 (0.0-1.4)	2.6(0.11)	**0.3* (0.1-1.0)***	**3.9(0.048)***	0.9 (0.0-32.5)	0.0(0.93)	2.5 (0.3-18.7)	
	Young adult	3.1 (0.8-12.3)	2.7(0.10)	1.3 (0.7-2.6)	0.7(0.41)	9.1 (0.5-169.9)	2.3(0.13)	3.5 (0.4-28.8)	1.4(0.24)
	Later adult	0.2 (0.0-1.6)	2.4(0.12)	0.2 (0.1-1.1)	3.6(0.06)	**44.5* (2.5-779.1)***	**7.0(0.008)***	2.1 (0.2-18.5)	0.5(0.49)

*Significant at the .05 level, two-sided test

[1]Assessed in Part 2 sample due to having part 2 controls. Controls for the model include int (1-5 intervals), and also include significant variables from demographic and parent psychopathology, details in following footnotes

[2]Models controls for int(1-5 intervals), countries, demographics (sex, age, time-varying education), interaction between int intervals(13-19,20-29,30+) and age, education. For parent psychopathology, controlling for number of parental disorders (dummies for 1, 2+ disorders).

[3]Models controls for int(1-5 intervals), countries, demographics (sex, age, time-varying education), interaction between int intervals(13-19,20-29,30+) and age, education. For parent psychopathology, controlling for number of parental disorders (dummies for 1, 2+ disorders).

[4]Models controls for int(1-5 intervals), countries, demographics (sex, age, time-varying education), interaction between int intervals(13-19,20-29,30+) and age, education. For parent psychopathology, controlling for types of parental disorders (6 dummies).

[5]Models controls for int(1-5 intervals), countries, demographics (sex, age, time-varying education), interaction between int intervals(13-19,20-29,30+) and age, education. Parent psychopathology not controlled for due to insignificance in previous models.

For peer review only - http://bmjopen.bmj.com/site/about/guidelines.xhtml

Association between childhood adversities and suicidality

For peer review only

For peer review only - http://bmjopen.bmj.com/site/about/guidelines.xhtml

Association between childhood adversities and long-term suicidality among South Africans: Results from the South African Stress and Health Study

For peer review only

Association between childhood adversities and suicidality

ABSTRACT

Objective:

Suicide and suicidal behaviours are significant public health problems and a leading cause of death worldwide and in South Africa. We examined the association between childhood adversities and suicidal behaviour over the life course.

Methods:

A national probability sample of 4,351 South African adult participants (aged 18 years and older) in the South African Stress and Health (SASH) study was interviewed, as part of the World Mental Health Survey initiative. Respondents provided socio-demographic and diagnostic information, as well as an account of suicide-related thoughts and behaviours. Outcomes were defined as suicide attempts and suicidal ideation in the total sample, and suicide plans and attempts among ideators. Childhood adversities included physical abuse, sexual abuse, parental death, parental divorce, other parental loss, family violence, physical illness and financial adversity. The association between suicidality and childhood adversities was examined using discrete-time survival models.

Results:

More than a third of respondents with suicidal behaviour experienced at least 1 childhood adversity, with physical abuse, parental death and parental divorce the most prevalent adversities. Physical abuse, sexual abuse and parental divorce were identified as significant risk markers for lifetime suicide attempts, while physical abuse and parental divorce were significantly correlated with suicidal ideation. Two or more childhood adversities were associated with a 2-fold higher risk of lifetime suicide attempts. Sexual abuse (OR=9.3, childhood, parental divorce (OR=3.1)

For peer review only

and physical abuse (OR=2.2) had the strongest associations with lifetime suicide attempts. The effect of childhood adversities on suicidal tendencies varied over the *life course.* For example, sexual abuse was significantly associated with suicide attempts during childhood and teen years, but not during young and later adulthood.

Conclusions:

Childhood adversities, especially sexual abuse, physical abuse and parental divorce are important risk factors for the onset and persistence of suicidal behaviour, with this risk greatest in childhood and adolescence. The risk for suicidal behaviour was greatest in childhood and adolescence. Suicidal risk in childhood and adolescence was significantly associated with the following childhood adversities: sexual abuse, physical abuse and parental divorce.

Keywords: Childhood adversities, suicidal ideation, suicidal attempts

For peer review only

Association between childhood adversities and suicidality

INTRODUCTION

Suicide and suicidal behaviour are significant public health problems. Suicide is one of the leading causes of death worldwide with almost 1 million people committing suicide each year [1]. This figure is likely to grow to approximately 1.2 million suicides in 2020 [2]. In South Africa, the annual rate of suicide is high [3, 4] mirroring international trends [5]. So, too, are rates of suicidal behaviour with an estimated prevalence of 9.1% for lifetime suicidal ideation and 2.9% for suicide attempts among South Africans according to the South African Stress and Health Survey (SASH) [6].

Despite the enormity of the problem, the aetiology of suicidal behaviour is not fully understood. There are controversies in the literature regarding prior psychiatric disorder and risk for suicide attempts. While some authors have argued that pre-existing disorder is an important risk factor (7-11], others have argued that suicide attempts are not neccessarily associated with prior psychopathology [12]. Genetic factors also play an important role in suicidal behaviour [13-16]. While there is stronger evidence pointing towards environmental or experiential factors [17, 18] such as exposure to childhood adversities (19-28]. Recent multi-level country data from the World Mental Health Surveys (WMHS) initiative has allowed for cross-national comparisons of suicidality. The WMHS investigated the association between childhood adversities and suicidal behaviour [20], the persistence of suicidality over time, and the extent to which associations between childhood trauma and suicidality changed over the life course. The WMHS found a dose-response relationship between the number of adversities and suicidal behaviour. Sexual abuse and physical abuse were the strongest risk factors for both the onset and persistence of suicidal behaviours, with the risk for suicidality greatest during childhood (age 4-12 years) and adolescence (age 13-19 years) [20].

For peer review only

Association between childhood adversities and suicidality

Numerous studies have examined the link between childhood sexual abuse and suicidality [29-41]. All of these authors have found that exposure to childhood sexual abuse increases the risk for mental disorders, including suicidality. Furthermore, the majority of studies that have focused on the link between childhood physical abuse and suicidality have found that exposure to childhood physical abuse increases the risk for suicidality [42, 43]. There also appears to be an association between the number of childhood adversities experienced and the later suicidal behaviour [21, 23, 24, 44, 45].

Exposure to early life stress is prevalent among South Africans. In one sample of South African rural youth, the prevalence of physical and sexual abuse was shown to be very high with 94.4% of males exposed to physical abuse and 39.1% of females to sexual abuse [46]. More than a quarter of adults who were interviewed endorsed exposure to childhood adversity (parental death, parental separation or parental divorce) in the SASH study [47]. Significantly more females were prone to be victims of domestic violence than men [47]. Women also reported twice as many suicidal attempts than the male participants in the SASH study [9].

Objective

We report in more detail on data from a South African dataset gathered as part of the World Mental Health Surveys, which allowed for comparison with data from the overall cross-national sample. This data are particularly interesting as South Africa is a middle income African country with high rates of violent trauma exposure. The present study aimed to examine the relationship between the type and frequency of childhood adversity exposure to suicidal behaviour over the life trajectory of South Africans, given that there are no published nationally representative data that may be useful in informing both clinical practice and policy.

For peer review only

Association between childhood adversities and suicidality

METHODS

Sample

Data for the SASH Study were collected between January 2002 and June 2004. WMH surveys were carried out in 21 countries which included Nigeria and South Africa [48]. For detailed information on study methods see Williams et al. (2004) [48]. The research protocol for the SASH study was approved by the Human Subjects Committee of the University of Michigan, by Harvard Medical School ethics committee and by a single project assurance of compliance from the Medical University of South Africa (MEDUNSA), and by the National Institute of Mental Health. It was a national probability sample of 4,351 South African adults (persons aged 18 years and older) living in households or in hostel accommodation. All racial and ethnic groups were represented, with the sample selected using a three-stage probability sample design. The response rate was 85.5%.

Sampling approach

Sampling was divided into three stages. Primary sampling units was selected during the first stage, which was based on the 2001 SA census Enumeration Areas (EAs). The second stage involved sampling of household units within clusters selected in each EA. South Africans in both urban and rural areas were sampled. Sampled residences were stratified into 10 diverse housing categories: Rural–commercial, agricultural, rural traditional subsistence areas, African townships, informal urban or peri-urban shack areas, Coloured townships, Indian townships, general metropolitan residential areas, general large metropolitan residential areas, and domestic servant accommodation in urban areas. During the third stage, one adult respondent in each sampled housing unit was selected. A total of 5089 households was selected. Field interviews were conducted with 4433 (87.1%) of designated respondents. Based on quality control, 4351

For peer review only - http://bmjopen.bmj.com/site/about/guidelines.xhtml

interviews were retained for use in the analysis. There were no differences in response rates across the four designated racial groups. (white, Coloured [mixed racial origin], Indian, black). According to the 2001 Census statistics, 79.% people in South Africa are Black African, 8.9% are coloured, 9.6% are white, and 2.5% are Indian/Asian [49].

Diagnostic Interview

SASH used version 3 of the World Health Organization Composite Diagnostic Interview (WHO CIDI) [50]. Interviewers were trained within a one week period and conducted the interviews in seven different languages, namely English, Afrikaans, Zulu, Xhosa, Northern Sotho, Southern Sotho, and Tswana. Translations of the CIDI into several native South African languages were conducted in accordance with WHO requirements. Multilingual and bilingual expert panels conducted the back-translations [51, 52]. Informed consent was obtained from participants after a complete description of the study was provided. Respondents provided socio-demographic and diagnostic information, as well as an account of suicidal behaviours during the interviews. The core diagnostic assessment of mental disorders included anxiety disorders (panic disorder, agoraphobia, social phobia, generalized anxiety disorder, post-traumatic stress disorder), mood disorders (major depressive disorder, dysthymia), substance use disorders (alcohol abuse, alcohol dependence, drug abuse, drug dependence) and intermittent explosive disorder [53, 54]. ~~Overall, percentages were weighted to adjust for differences in selection probabilities, differential non-response, oversampling of cases, and residual differences on sociodemographic variables between the sample and the population (Williams et al, 2004; Stein et al, 2010).~~

Suicidal behaviour

The CIDI 3.0 module on suicidal behaviour was used to assess the age-of-first-onset, age of most recent episode, and lifetime occurrence of suicidal ideation, suicide plans and suicide attempts.

For peer review only

Association between childhood adversities and suicidality

Suicidal ideation, suicide plans and suicide attempts was assessed with questions such as "Have you ever seriously thought about committing suicide?", "Have you ever made a plan for committing suicide?", and "Have you ever attempted suicide?", respectively. Ideators only proceeded to answer questions about plans ("Have you ever made a plan for committing suicide?") and attempts ("Have you ever attempted suicide?"). Information on the age of first occurrence of the three main outcomes was obtained. To get a better understanding of the progression from ideation to attempt, tThe outcomes considered in this study were: suicide attempts in the total sample; suicide ideation in the total sample; suicide plans among ideators; suicide attempts among ideators with a plan (planned attempts), and suicide attempts among ideators in the absence of a plan (unplanned or impulsive attempts).

Childhood adversities

Physical abuse, sexual abuse, parental death, parental divorce, other parental loss, family violence, physical illness and financial adversity were the various childhood adversities assessed. Biological and non-biological parents were included in measures of parental death, divorce or other parental loss. Financial adversities were assessed with questions on whether the family had insufficient funds to pay for basic necessities. Questions about repeated fondling, attempted rape or rape were asked to assess for sexual abuse. This comprised the following "The next 2 questions are about sexual assault: (i) The first is about rape. We define this as someone either having sexual intercourse with you or penetrating your body with a finger or object when you did not want them to, either by threatening you or using force, or when you were so young that you didn't know what was happening. Did this ever happen to you?", and (ii)"Other than rape, were you ever sexually assaulted or molested?". A modified version of the Conflict Tactics Scale (CTS2) was used to assess ffamily violence and physical abuse [55]. Respondents were classified

as having experienced *physical abuse* when they indicated that, when they were growing up, their father or mother (includes biological, step, or adoptive parents) slapped, hit, pushed, grabbed, shoved, or threw something at them, or that they were beaten as a child by the persons who raised them. Family violence was assessed as present when respondents indicated that they (i) "were often hit, shoved, pushed, grabbed, or slapped while growing up" *or* (ii) "witnessed physical fights at home, like when your father beat up your mother?" A standard chronic conditions checklist assessed for life-threatening physical illnesses in childhood [56].

Data analysis

All data analyses were processed and analysed centrally by a team of statisticians at the Harvard School of Public Health (Boston, USA) using the SAS version 9.1.3 software package. Discrete-time survival analysis with time-varying covariates was used to study the risk factors of lifetime suicide ideation, plans and attempts. Data were weighted to adjust for the stratified multistage sample design, differential probability of selection within households as a function of household size and clustering of data, and differential non-response. Overall, percentages were weighted to adjust for differences in selection probabilities, differential non-response, oversampling of cases, and residual differences on sociodemographic variables between the sample and the population [48, 57]. A post-stratification weight was also used to make the sample distribution comparable, for age, sex, ~~gender,~~ and province, with the population distribution in the 2001 South African census. Both weighted and geographic clustering of data were taken into account in the data analyses by using a jackknife repeated replications simulation method implemented in SAS macro 14. The survival coefficients were exponentiated and are reported below in the form of odds ratios.

For peer review only

Association between childhood adversities and suicidality

The association between suicidality and childhood adversity was examined using discrete-time survival models with the analysis unit being person-years. Bivariate analyses (considering one adversity at a time) and multivariate analyses (considering all adversities simultaneously) were conducted. Two types of multivariate models were tested: multivariate additive models (simultaneously considering all childhood adversities) and multivariate interactive models (with number and type of childhood adversities experienced by each respondent included as dummy variables). The analysis also examined interactions between the life stage (13-19 years, 20-29 years, 30+ years) of respondents and each childhood adversity, as well as the influence each adversity had on early-, middle- and later- onset suicidality. Analyses were conducted using SUDAAN version 8.1 to adjust for clustering and weighting. Odds ratios (ORs) with a 95% confidence interval (CIs) are reported. Wald X^2- tests were used to examine multivariate significance. Associations between adversities and suicide outcomes were adjusted for sex ~~gender~~, age, educational level, marital status, interactions between demographic variables, life course and parental psychopathology. Analyses also examined the influence of respondents' lifetime mental disorders on suicidality, as well as interactions between sex ~~gender~~ and each childhood adversity. Statistical significance using two-sided tests was set at p <.05 [20].

RESULTS

Demographic details

In the sample, (n = 4351), there were slightly more female (53.7%) than male respondents. There were more black (76.2%) than coloured (10.4%), white (10%), and Indian/Asian (3.4%) respondents. Furthermore, half of the sample was married and most were unemployed (69.2%), had less than 12 years of education (62.7%) and lived in an urban area (59.7%) (see Table 1).

For peer review only

Association between childhood adversities and suicidality

Prevalence of childhood adversities among the total sample

Figure 1 provides a schematic representation of the suicidality data reported in the sections which follow. In the total sample, 35.4% of participants with one adversity had a suicide attempt, compared with 23.4% with one adversity who had not made an attempt. Physical abuse (24.9%), parental divorce (14.2%) and parental death (11.6%) were most prevalent among those suicide attempters. Among those exposed to one childhood adversity, without a suicide attempt, the two most prevalent adversities reported were physical abuse (12.2%) and parental death (11.3%). In the total sample 15.4% of participants exposed to two or more adversities had a suicide attempt. In contrast, 8.6% of participants exposed to two or more adversities had not made an attempt (Table 2).

Prevalence of childhood adversities among suicidal ideators in the total sample

In the sample as a whole, 35.9% of those with one adversity had suicidal ideation compared with 22 .7% of those with one adversity who had no ideation. The most prevalent adversities associated with suicidal ideation were physical abuse (21.1%), parental death (13.9%), and parental divorce (7.9%). Among those without suicidal ideation, physical abuse (11.8%) and parental death (11.3%) were the most commonly endorsed childhood adversities. Of those who endorsed two or more childhood adversities, 10.8% reported suicidal ideation and 8.6% did not (Table 2). In summary, the most prevalent childhood adversities reported among the total sample with/without suicidal ideation were firstly, physical abuse and secondly, the death of a parent.

Prevalence of suicide attempts in the total sample

In the total sample, 24.9% of those with childhood physical abuse had attempted suicide while 12.2% of respondents with no physical abuse had no attempt. Of those exposed to parental divorce, 14.2% had attempted suicide and 4.8% had made no attempt. The second most prevalent

For peer review only

Association between childhood adversities and suicidality

childhood adversity was parental death with 11.6% of those with parental death attempting suicide and 11.3% of those with parental death with no attempts (Table 2).

Prevalence of childhood adversities among suicidal ideators

With/without a plan

Among suicidal ideators with a plan, 32.9% had experienced one childhood adversity. Among ideators with no plan, 41.7% had one childhood adversity. Among ideators with a plan, the following were the most prevalent childhood adversities: physical abuse (24.3%), parental death (12.2%), and parental divorce (9.7%). Among ideators without a plan, 27.9% endorsed physical abuse, 16.1% parental death, and 9.2% parental divorce (see Table 2). In both groups (ideators with and without a plan), physical abuse was the most prevalent childhood adversity, followed by parental death and parental divorce.

With or without an attempt

Among suicidal ideators who had attempted suicide, 35.4% were exposed to one childhood adversity and 15.4% were exposed to two or more childhood adversities. In the group of ideators who had made an attempt, 24.9% had experienced physical abuse, 14.2% parental divorce, and 11.6% parental death (Table 2). 40.5% of those with one adversity, and 9.6% of those exposed to two or more adversities were suicidal ideators with no attempts. In this group, the most prevalent adversities were physical abuse (24.5%), parental death (15.6%) and parental divorce (6.7%) reported (Table 2).

Among all ideators (with/without a plan, with/without an attempt), the most prevalent childhood adversity was physical abuse, followed by parental death and parental divorce. Of note, in the group of ideators with an attempted suicide parental divorce was more prevalent than parental death.

For peer review only

Association between childhood adversities and suicidality

Bivariate and multivariate results: Type of childhood adversity

Bivariate and multivariate analyses were performed to examine the associations between the different childhood adversities (physical abuse, sexual abuse, parental death, parental divorce, other parental loss, family violence, physical illness, financial adversity) and lifetime suicidal ideation, plans and attempts.

In the total sample, bivariate and multivariate analysis revealed significant associations between (i) sexual abuse (bivariate: OR=7.9, p=0.003; multivariate: OR=7.6, p=0.003), (ii) physical abuse (OR 2, p=0.007; OR 2.0, p=0.006) and (iii) parental divorce (OR 2.8, p<.001; OR 2.7, p=0.001), and lifetime suicide attempts. Among ideators in the sample, physical abuse (OR=1.7, p<.001; OR=1.7, p<.001) was significantly associated with suicidal ideation. Multivariate analyses revealed an additional association with suicidal ideation, namely parental divorce (OR = 1.6, p=0.038). The relationship between childhood adversities and lifetime plans was not statistically significant. However, a significant association was found between parental divorce and lifetime suicidal attempts among ideators (OR=3.0, p<.001; OR=3.1, p=0.023) (Table 3Table available from authors).

Multivariate analysis also revealed a significant association between (i) sexual abuse (OR=7.6, p=0.003), (ii) physical abuse (OR 2.0, p=0.006) and (iii) parental divorce (OR 2.7, p=0.001) and lifetime suicide attempts, in the total sample. Physical abuse (OR=1.7, p<.001) and parental divorce (OR = 1.6, p=0.038) were both significantly associated with suicidal ideation in the overall sample. Among ideators, no significant associations were found between any of the childhood adversities and lifetime plans. However, the relationship between parental divorce and lifetime suicidal attempts among ideators was significant (OR=3.1, p=0.023) (Table 3).

For peer review only

Association between childhood adversities and suicidality

Findings from multivariate analysis, therefore, confirm findings of bivariate analysis for all groups, except for ideators. Among ideators bivariate analysis revealed a significant relationship between physical abuse and suicidal ideation. This was confirmed in multivariate analysis where the association between parental divorce and suicidal ideation was significant for the whole sample.

Bivariate associations between the number of adversities and lifetime suicidality

The relationship between the number of childhood adversities and lifetime suicidal ideation, plans and attempts was further examined. There was a significant relationship between the number of childhood adversities and lifetime suicide attempts. Two or more childhood adversities were associated with a 2-fold higher risk of lifetime suicide attempts in the total sample (OR=2.1, p<.001). A significant relationship was also established between one, as well as two or more adversities with ideators in the total sample. Among ideators, no significant association was found between the number of childhood adversities and lifetime plans. A significant relationship was found between two or more adversities and lifetime attempts among ideators (OR=2.7, p=0.016), indicating a more than 2-fold higher risk of lifetime suicide attempts in this group (Table 4).

Multivariate associations between number of childhood adversities and lifetime suicidality

In the final multivariate model which included 2 or more adversities as a predictor variable, sexual abuse (OR=9.3, p<.001), childhood physical abuse (OR=2.2, p=0.003) and parental divorce (OR=3.1, p<.001) retained significant associations with lifetime suicide attempts in the total sample. Physical abuse (OR=2.1, p<.001), parental death (OR=1.7, p=0.010), parental divorce (OR=1.9, p=0.004) and other parental loss (OR = 2.1, p=0.004) were significant predictors of suicidal ideation. Physical abuse was associated with a lower odds of lifetime

For peer review only

Association between childhood adversities and suicidality

suicide plans among ideators (OR = 0.4, p=0.038). ~~Physical abuse (OR=0.4, p=0.038) was significantly associated with lifetime plans among ideators.~~ There were no significant associations between childhood adversities and lifetime attempts among those with suicidal ideation (Table 5).

Associations between the types of childhood adversity and lifetime suicidality over the life course

Multivariate analyses were performed to examine the association between the types of childhood adversity and lifetime suicidal ideation, plans and attempts during childhood years (age 4- 12), teenage years (age 13-19), young adulthood (age 20-29) and later adulthood (30 years and older) (Tables available from authors).

Childhood years (4-12). Sexual abuse (OR=61.6, CI=4.5-841.0, p=0.002) in early childhood (4-12 years of age) was significantly associated with lifetime suicide attempts in the total sample (OR = 61.6, CI=4.5-841.0, p=0.002). Both sexual abuse (OR=34.8, CI= 3.1-392.6, p=0.003) and physical abuse (OR=3.7, CI=1.0-13.4, p=0.041) were associated with a higher risk for suicidal ideation among the total sample. No significant associations were found between any of the childhood adversities and lifetime plans in the group of ideators. Among those with suicidal ideation, parental death (OR=2.2, CI=1.1-4.3,~~2.7,~~ p=0.021) was significantly associated with suicide attempts in childhood years.

Teen years (13-19). Sexual abuse (OR=20.3, CI=2.0-210.2, p=0.010), physical abuse (OR=3.7, CI=1.5-9.2, p=0.004), and parental divorce (OR=4.6, CI=1.7-12.1, p=0.002) were significantly associated with suicide attempts in the total sample of teenagers. Physical abuse (OR=3.6, CI=2.2-5.9, p<.001) and parental death (OR=2.2, CI=1.1-4.3, p=0.021) significantly increased

For peer review only

Association between childhood adversities and suicidality

the risk for suicidal ideation among the total group of teens. Physical illness (OR=9.9, CI=1.8-54.0, p=0.007) significantly increased the risk of suicidal plans in teens with suicidal ideation. Suicide attempts among teens with suicidal ideation was significantly predicted by parental divorce (OR=4.3, CI=1.1-17.0, p=0.035).

Young adulthood (20-29). None of the childhood adversities were significantly associated with lifetime suicide attempts during young adulthood in the sample overall. An explanation could be that suicide attempts spike earlier and later in life among South Africans, contributing to the lack of significance. Parental loss other than parental death was significantly associated with suicidal ideation (OR=2.9, CI=1.2-7.4, p=0.019).

Later adulthood (≥ 30). Childhood physical abuse (OR=2.2, CI=1.0-4.8, p=0.035) was significantly predictive of suicidal attempts. The likelihood of suicidal ideation significantly increased in later adulthood if parental loss other than parental death (OR=5.1, CI=2.1-12.1, p<.001) or physical illness had been present during childhood (OR=4.3, CI=1.1-15.9, p=0.028). No significant relationship was found between any of the childhood adversities and lifetime plans in the group of ideators although a significant relationship was found between two or more adversities and lifetime plans among those who were ideators (OR=44.5, CI=2.5-779.1, p<0.008). None of the childhood adversities were significantly associated with suicide attempts among ideators in this age group.

DISCUSSION

Rates of childhood adversities and suicidal behaviours were both high among South Africans, with more than a third of respondents in the total sample who attempted suicide experiencing one childhood adversity, and 15.4% experiencing two or more adversities. Overall, physical abuse,

For peer review only

Association between childhood adversities and suicidality

sexual abuse, parental divorce and physical illness were far more prevalent in those with a suicide attempt than in those without. The most prevalent childhood adversities endorsed overall were physical abuse followed by parental death. Physical abuse, parental divorce and death of a parent were also the most prevalent adversities experienced in those with a suicide attempt as well as in those with suicidal ideation. These findings are somewhat dissimilar to other country samples; for example in the 21 countries that participated in the WMHS, physical abuse (29.3%), family violence (24.8%) and neglect (19.3%) were the most prevalent childhood adversities among those with a lifetime suicide attempt, while physical abuse (20.6%), family violence (17.6%) and death of a parent (14.2%) were most often reported among participants with lifetime suicidal ideation [20]. Cross-nationally, it would appear that physical abuse is the commonest childhood adversity associated with lifetime suicide attempts and ideation [20].

The estimate lifetime prevalence of 2.9% for attempted suicide among South Africans is close to the rates of 4.6% and 4.1% reported for general and Black populations respectively in USA. In addition the 9.1% estimated prevalence of suicide ideation is comparable with previous estimates from studies in South African clinical samples. Joe et al. (2008b) reported for the first time on the rates of suicide ideation, plan and attempts among the different ethnic groups, in data from the SASH study [6]. Overall, the results suggest that people in SA engage in suicidal thought and behaviours at levels nearly comparable with those of Western nations.

When examining suicidal behaviour risk in the context of childhood adversity, sexual abuse, physical abuse and parental divorce emerged as significant risk factors for lifetime suicide attempts in the total sample. Furthermore, physical abuse and parental divorce were significant risk factors for suicidal ideation in the total sample, while parental divorce emerged as a significant risk factor among ideators with lifetime suicide. These findings are largely consistent

For peer review only

Association between childhood adversities and suicidality

with the data from the overall cross-national WMHS, which found that physical and sexual abuse significantly increased the likelihood of suicidal ideation and attempts, while neglect was a risk factor for suicidal behaviour in multivariate additive analyses [20].

Of the adversities implicated, sexual and physical abuse were more significant risk factors than other adversities, highlighting the fact that intrusive and aggressive experiences in childhood may have more devastating and longer lasting effects [58]. This may be due to the extreme powerlessness and loss of control that such abuse causes, or to physically aggressive assaults resulting in the devaluation of one's body and consequent susceptibility to self harm [28]. In a country with high rates of sexual and physical abuse [46] this is particularly concerning. The impact of parental divorce on suicidality supports previous findings that parental divorce, if accompanied by other adversities such as childhood abuse, increases the risk of suicidal behaviour [59].

We also found that exposure to *two or more childhood adversities* significantly increased the risk of suicide attempts among ideators. This confirms earlier work showing exposure to multiple childhood adversities increases the risk of suicidal behaviour [21, 23, 24, 60, 61]. Bruffaerts et al (2010) found a sub-additive effect with regards to the onset of suicidal behaviour when considering multiple adversities [20]. Thus, the impact of multiple adversities was not equal to the sum of the odds ratios of individual adversities. In the overall WMHS analysis exposure to multiple childhood adversities had a significant effect on the persistence of suicide when considering every additional childhood adversity exposed to, however in the current study it was not possible to stratify the number of adversities beyond two or more adversities (i.e. into more than 2 categories) given the relatively small number of cases in the sample overall with non-fatal suicidal behaviour. Physical abuse, parental death, parental loss other than through death, and

For peer review only

Association between childhood adversities and suicidality

parental divorce emerged as independent risk factors for suicidal ideation in the total sample. Moreover, the effects of childhood adversities on suicidal tendencies tended to differ over the *life course*. Consistent with nationally representative data in WMHS, childhood adversities were associated with the highest risk of suicide attempts in childhood, with a decrease in risk in adolescence and young adulthood, followed by an increase in risk again during later adulthood [20].

In *childhood*, sexual abuse was significantly associated with lifetime suicide attempts in the total sample, while sexual and physical abuse were significantly associated with suicidal ideation. Among suicidal ideators, parental death was significantly associated with lifetime suicide attempts. Exposure to childhood sexual abuse, physical abuse or parental divorce significantly increased suicide attempts during *teenage years*, while physical abuse and parental death were associated with suicidal ideation in teens. Among teen suicidal ideators, physical illness was significantly associated with suicidal plans, while parental divorce was associated with suicide attempts. These findings emphasize the need to focus suicide prevention strategies at youth in particular. In *young adulthood,* parental loss other than the death of a parent was significantly associated with suicidal ideation in the total sample. Interestingly, childhood physical abuse was identified as a significant risk factor for suicidal attempts in *later adulthood*, while childhood physical illness and parental loss other than the death of a parent significantly increased the risk for ideation.

Similar to findings from SASH, childhood sexual abuse emerged as a particularly robust risk factor for suicide attempts in younger participants in the WMH cross-national analysis, with a 10.9 times higher odds of suicide attempts in children, a 6.1 times higher likelihood in adolescents and a 2.9-fold risk in young adults who were exposed [20]. This is in keeping with

For peer review only

Association between childhood adversities and suicidality

Enns hypothesis that sexual abuse results in suicidal behaviour at a younger age [21]. Consistent with other studies, childhood physical and sexual abuse, in particular, emerged as risk factors for the emergence and persistence of suicidal behaviour, especially in adolescence. Loss of a parent, physical ill-health and family violence has also been found to be associated with persistence of suicidality [20, 28, 58]. These findings extend previous work done in other developing countries that have found childhood adversities to be a significant risk factor for suicidality [20, 62-64].

Limitations

The following limitations need to be highlighted. First, recall bias might have impacted on the accuracy of recall of childhood adversities. This said, participants were asked questions about childhood adversities in sequence which may have facilitated more accurate recall [65]. Systematic reviews have also found that recall of past experiences can be accurate and can provide valuable data [66, 67]. Thus, there is evidence to support the validity of accurate recall of childhood adversities [67]. Further, studies have shown that responses to questions on childhood adversities, similar to those asked in the SASH study, generally remain stable over time [68, 69]. We recommend that future studies examine ethnicity in relation to adversity and suicidal~~e~~ outcomes. ~~variables.~~Second ~~Third~~, in view of ~~owing to~~ the cross-sectional design, more detailed, temporal information on~~s~~ ~~regarding~~ childhood adversities and suicidal incidents was~~ere~~ not obtained~~assessed~~. Third, variables such as culture, ethnicity and mental status at the time of the interview may have influenced the recall and reporting of suicidal behaviour. -It is possible that response bias may have been particularly skewed to disenfranchised South Africans (e.g. poor, young, urban an black respondents), who may have been too afraid to divulge information on suicidality. Stigma associated with mental health problems may have also played a role in the

For peer review only

reporting suicidal tendencies. Thus, participants' mental health status, ethnicity, culture and generational factors may have also contributed to the under-reporting of suicidality. It is possible that individuals reporting childhood adversities may have also been more likely to report suicidal behaviour, while those not reporting childhood adversities may have underreported suicidality. ~~However, it is much more likely that adversities and suicidality were under-reported rather than over-reported (Wilsnack et al, 2002; Hardt & Rutter, 2004; Joe et al, 2008; Bruffaerts et al, 2010). Some of the participants might have been scared to tell the interviewers about their suicidal behaviours.~~ Stigma and mental health status (e.g. depressed persons may be more inclined to report suicidality and more likely to remember negative childhood experiences) ~~associated with mental health~~ may ~~have~~ also be contributory factors~~played a role in reporting suicidal tendencies~~. In addition, some participants may have been afraid to report suicidal behaviours.~~. The status of the participant's mental health,~~ T~~t~~he role of ethnicity, culture and generational factors may have also contributed to the under-reporting of suicidality. Overall, it is much more likely that adversities and suicidality were under-reported rather than over-reported [9, 20, 67, 70]. Fourth, we do not assess for self-mutilating behavior. The importance of discriminating suicidal behaviour from non-suicidal self-mutilation cannot be underestimated. Fifth, the survey was conducted in adults living in households and hostel quarters thus the findings are not generalizable to homeless and institutionalized persons who were not included in the survey. Sixth, the CIDI instrument which was used in this study is a lay-administered instrument which does not include an assessment of several key DSM-IV diagnoses (such as bipolar disorder and psychosis), are associated with elevated rates of suicidality. As a result, some participants with suicidality may have not have been diagnosed with a disorder. Furthermore, in view of the large confidence intervals and small sample sizes for some of these analyses caution is required in

For peer review only

Association between childhood adversities and suicidality

drawing conclusions. In addition, we did not control for other unmeasured causes of childhood adversities and suicida~~i~~lity, or protective (resiliency) factors that may have contributed to the associations observed in these data. Both other risk and resiliency factors may have contributed to both the prevalence of non-fatal suicidal behaviours and to the associations with different forms of childhood adversity and warrant further investigation. Lastly, it is important to point out that these data were collected approximately 10 years ago. Notwithstanding these limitations, this study represents the first investigation among South Africans of a wide range of childhood adversities and their impact on the onset and persistence of suicidality over the life course.

Conclusions

Childhood adversities especially sexual abuse, physical abuse and parental divorce are ~~important risk factors for~~ associated with the onset and persistence of suicidal behaviour with the risk greatest in children and adolescents. Public health efforts aimed at prevention of early childhood sexual and physical abuse, in particular, may have a significant impact on reducing suicidality over the life course and improving mental health outcomes.

For peer review only

Association between childhood adversities and suicidality

REFERENCES

1. World Health Organization. Suicide Prevention (SUPRE). Geneva, Switzerland. 2007. http://www.who.int/mental_helath/prevention/suicide/suicideprevention/en/)

2. Murray, C.L., Lopez, A.D. The global burden of disease: a comprehensive assessment of mortality and disability from disease, injuries and risk factors in 1990 and projected to 2020. Cambridge, MA: Harvard University Press, 1996.

3. Burrows, S., Laflamme, L. Pattern analysis of suicide mortality surveillance data in urban South Africa. Suicide and Life- Threatening Behaviour 2008;**38**:209-220.

4. Meel, B.I. Epidemiology of suicide by hanging in Transkei. South Africa. Am J Forensic Med Pathol. 2006;**27**:75-78

5. Flisher, A.J., Liang, H., Laubscher, R. Suicide trends in South Africa, 1968-90. Scand J Public Health 2004;**32**:411-418.

6. Joe, S., Stein, DJ., Seedat, S., Herman, A., Williams, DR. non-fatal suicidal behavior among South Africans: Results from the South Africa Stress and Health Study. Social Psychiatry Epidemiology 2008;**43**(6):454–461.doi:10.1007/s00127-008-0348-7.

7. Beautrais, A.L., Joyce, P/R/. & Mulder, R.T. (1996). Risk factors for serious suicide attempts among youth aged 13 through 24 years. J Am Acad Child Adolesc Psychiatry 1996;**35**(9):1174-1182.

8. Harrison, EC, Barraclough, B. (1997). Suicide as an outcome for mental disorders: A meta-analysis. Br J Psychiatry 1997;**170**:205-228

For peer review only

Association between childhood adversities and suicidality

9. Joe, S., Stein, D.J., Seedat, S., et al. Prevalence and correlates of non-fatal suicidal behaviour among South Africans. Br J Psychiatry 2008;**192**:310-311.
10. Nock, M.K., Borges, G., Bromet, E.J., et al. Suicide and Suicidal Behaviour. Epidemiologic Reviews 2008;**30**:133-154.
11. Nock, M.K., Borges, G., Bromet, E.J., et al. (2008b). Cross-national prevalence and risk factors for suicidal ideation, plans and attempts. *British Journal of Psychiatry*, 192, 98-105.
12. Nock, M.K., Hwang, I., Sampson, N.A., et al. Cross-national analysis of the associations among mental disorders and suicidal behaviour: Findings from the WHO World Mental Health Surveys. PLos Medicine 2009;**6**(8).e1000123.
13. Bondy, B., Buettner, A., Zill, P. Genetics of suicide. Molecular Psychiatry 2006;**11**:336-351.
14. Kohli, M.A., Salyakina, D., Pfennig, A., et al. Association of genetic variants in the neurotrophic receptor encoding gene NTRK2 and a lifetime history of suicide attempts in depressed patients. Arch Gen Psychiatry 2010;**67**:348-59.
15. Roy, A., Hu, X-Z., Janal, M.N., & Goldman, D. Interaction between childhood trauma and serotonin transporter gene variation and suicide. Neuropsychopharmacology 2007;**32**:2046–2052
16. Risch, N., Herrell, R., Lehner, T., et al. Interaction between the serotonin transporter gene (5-HTTLPR), stressful life events, and the risk of depression: A meta-analysis. JAMA 2009;**301**:2462–2471.
17. Borges, G., Benjet, C., Medina-Mora, M.E., et al. Traumatic events and suicide related outcomes among Mexico City adolescents. J Child Psychol Psychiatry 2008;**6**:654-666.Weissman MM, Bland

For peer review only

RC, Canino GJ, Greenwald S, Hwu HG, Joyce PR, et al. (1999) Prevalence of suicide ideation and suicide attempts in nine countries. *Psychology Med, 29*: 9–17.

18. Brodsky, BS & Stanley, B. Adverse childhood experiences and suicidal behaviour. Psychiatry Clinical Northern America 2008;**31**:223-235

19. Bruffaerts, R., Demyttenaere, K., Borges, G., et al. Childhood adversities as risk factors for onset and persistence of suicidal beahviour. Br J Psychiatry 2010;**197**:20-27.

20. Enns, M.W., Cox, B.J., Afifi, T.O., et al. Childhood adversities and risk for suicidal ideation and attempts: a longitudinal population-based study. Psychological Medicine 2006;**36**:1769-1778.

21. Johnson, J.G., Cohen, P., Gould, M.S., et al. Childhood adversities, interpersonal difficulties, and risk for suicide attempts during late adolescence and early adulthood. Arch Gen Psychiatry 2002;**59**:741-749.

22. Dube, S.R., Anda, R.F., Felitti, V.J., et al. Childhood abuse, household dysfunction, and the risk of attempted suicide throughout the life span: Findings from the Adverse Childhood Experiences Study. JAMA 2001;**286**:3089-3096.

23. Afifi, T.O., Enns, M.W., Cox, B.J., et al. Population attributable fractions of psychiatric disorders and suicide ideation and attempts associated with adverse childhood experiences. Am J Public Health 2008;**98**:946-952.

24. Burke, A.K., Galfalvy, H., Everett, B., et al. Effect of exposure to suicidal behavior on suicide attempt in a high-risk sample of offspring of depressed parents. J Am Acad Child Adolesc Psychiatry 2010;**49**:114-121.

For peer review only

Association between childhood adversities and suicidality

25. Labonte, B., Suderman, M., Maussion, G., Navaro, L., Yerko, V., Mahar, I., & Turecki, G. Genome-wide epigenetic regulation by early-life trauma. Arch Gen Psychiatry 2012;**69**(7):722-731.Doi:10.1001/archgenpsychiatry.2011.2287

26. Lipschitz, D.S., Winegar, R.K., Nicolaou, A.L., et al. (1999). Perceived abuse and neglect as risk factors for suicidal behaviour in adolescent inpatients. *The Journal of Nervous and Mental Disease*, 187, 32-39.

27. Ystgaard, M., Hestetun, I., Loeb, M., & Mehlum, L. Is there a specific relationship between childhood sexual and physical abuse and repeated suicidal behaviour? Child Abuse Neg 2004;**28**:863-875

28. Boudewyn, A., & Liem, J. Childhood sexual abuse as a presecutor to depression and self-destructive behavior in adulthood. J Trauma Stress 1995;**8**:445-459.

29. Brown, J., Cohen, P., Johnson, J.G., & Smailes, E.M. Childhood abuse and neglect: Specificity of effects on adolescent and young adult depression and suicidality. J Am Acad Child Adolesc Psychiatry 1999;**38**:1490-1496.

30. Bryant, S.L., & Range, L.M. Suicidality in college women who were sexually and physically abused and physically punished by parents. Violence Vict 1995;**10**:195-201.

31. Davidson, J.R.T., Hughes, D.C., George, L.K., & Blazer, D.G. The association of sexual assault and attempted suicide within the community. Arch Gen Psychiatry 1996;**53**:550-555

32. Fergusson, D.M., & Mullen, P.E. Childhood Sexual abuse – An evidence based perspective. Sage, CA: Thousand Oaks, 1999.

For peer review only

33. Finkelhor, D. Early and long-term effects of child sexual abuse: An update. Professional Psychology: Research & Practice 1990;**21**(5):325-330.

34. Finkelhor, D., & Hashima, P.Y. (2001). The victimization of children and youth: A comprehensive overview. In S.O. White (Ed.) Handbook of youth and justice. The Plenum series in crime and justice. Dordrecht: Plenum, 2001:49-78.

35. Holmes, W.C., & Slap, G.B. Sexual abuse of boys: Definition, prevalence, correlates, sequelae, and management. JAMA: JAMA 1998;**280**(21):1855-1862

36. Kendall-Tackett, K.A., Williams, L.M., & Finkelhor, D. Impact of sexual abuse on children: A review and synthesis of recent empirical studies. Psychol Bull 1993;**113**(1):164-180.

37. Martin, G. Reported family dynamics, sexual abuse, and suicidal behaviors in community adolescents. Arch Suicide Res 1996;**2**:183-195.

38. Peters, D.K., & Range, L.M. Childhood sexual abuse and current suicidality in college women and men. Child Abuse Negl 1995;**19**:335-341.

39. Putman, F.W. Ten-year research update review: Child sexual abuse. J Am Acad Child Adolesc Psychiatry 2003;**42**(3):269-278

40. Stepakoff, S. Effects of sexual victimization on suicidal ideation and behaviour in US college women. Suicide and Life-Threatening Behavior 1998;**28**:107-126.

41. Malinosky-Rummel, R., & Hansen, D.J. Long-term consequences of childhood physical abuse. Psychol Bull 1993;**144**:68-79

For peer review only

Association between childhood adversities and suicidality

42. Silverman, A.B., Reinherz, H., & Giaconia, R.M. The long-term sequelae of child and adolescent abuse: A longitudinal community study. Child Abuse Negl 1996;**20**:709-723

43. Chapman, D.P., Whitfield, C.L., Felitti, V.J., Dube, S.R., Edwards, V.J., & Anda, R.F. Adverse childhood experiences and the risk of depression in adulthood. J Affect Disord 2004;**82**:217-225

44. Dube, S.R., Felitti, V.J., Dong, M., Chapman, D.P., Giles, W.H., & Anda, R.F. Childhood abuse, neglect, and household dysfunction and the risk of illicit drug use: The adverse childhood experiences study. Pediatricc 2003;**111**:564-572.

45. Jewkes, R.K., Dunkle, K., Nduna, M., et al. Associations between childhood adversity and depression, substance abuse and HIV and HSV2 incident infections in rural South African youth. Child Abuse Negl 2010;**34**:833-841.

46. Seedat, S., Stein, D.J., Jackson, P.B., Heeringa, S.G., Williams, D.R., Myer, L. Life stress and mental disorders in the South African Stress and Health study. South African Medical Journal 2009a;**99:**375-382.

47. Williams, D.R., Herman, A., Kessler, R.C., et al. The South Africa Stress and Health Study: Rationale and Design. Metab Brain Dis 2004;**19**(1/2):135-147.

48. Statistics South Africa. Census 2001: Census in Brief. Pretoria: Statistics South Africa. 2001. Available from http://www.statssa.gov.za/census01/html/CInBrief/CIB2001.pdf (Accessed January 2014)

49. Kessler, R.C., Üstün, T.B. The World Mental Health (WMH) Survey Initiative Version of the World Health Organization (WHO) Composite International Diagnostic Interview (CIDI). Int J Methods Psychiatr Res 2004;**13**:61-98.

For peer review only

50. Seedat, S., Stein, D.J., Herman, A., et al. Twelve-month treatment of Psychiatric disorders in South Africa Stress and Health Study (World Mental Health Survey Initiative). Psychiatric Epidemiology 2008;**38**:211-220.

51. Seedat, S., Williams, D.R., Herman, A., et al. Mental health service use among South Africans for mood, anxiety and substance use disorders. South African Medical Journal 2009b;**99**:346-352.

52. World Health Organization. World Health Organization Manual of the international statistical classification of diseases, injuries and causes of death, ninth revision. Geneva, Switzerland, 1992.

53. American Psychiatric Association. Diagnostic and statistical manual of mental disorders (DSM-IV), 4th Edition. Washington: American Psychiatric Association Press, 1994.

54. Straus MA. Measuring Intrafamily Conflict and Violence: The Conflict Tactics (CT) Scales. Journal of Marriage and Family 1979;**41(1)**:75

55. Kessler, R.C., McLaughlin, K.A., Green, J.G. Childhood adversities and adult psychopathology in the WHO World Mental Health Surveys. Br J Psychiatry 2010;**197**:378-385.

56. Stein, D.J., Chiu, W.T., Hwang, I., et al. Cross-national analysis of the associations between traumatic events and suicidal behavior: Findings from the WHO World Mental Health Surveys. PloS ONE 2010;**5**(5):e10574.

57. Joiner Jr, T.E., Sachs-Ericsson, N.J., Wingate, L.R. Childhood physical and sexual abuse and lifetime number of suicide attempts: A persistent and theoretically important relationship. Behav Res Ther 2007;**45**:539-547.

For peer review only

58. Afifi, T.O., Boman, J., Fleisher, W., et al. The relationship between child abuse, parental divorce, and lifetime mental disorders and suicidality in a nationally representative adult sample. Child Abuse Negl 2009;**33**:139–147.

59. Bebbington, P.E., Cooper, C.C., Minot, S., et al. Suicide attempts, gender, and sexual abuse: data from the 2000 British Psychiatric Morbidity Survey. Am J Psychiatry 2009;**166**:1135-1140.

60. Molner, B, Buka, S, & Kessler, R. Child sexual abuse and subsequent psychopathology: results from the National Comorbidity Survey. American Journal Public Health 2001;**91**:753-760.

61. Borges, G., Angst, J., Nock, M.K., et al. Risk factors for the incidence and persistence of suicide related outcomes: a 10 year follow up study using the National Comorbidity Surveys. J Affect Disord 2008;**105**:25-33

62. Xing, X-Y., Tao, F-B., Wan, Y-H., et al. Family factors associated with suicide attempts among Chinese adolescent students: A national cross-sectional survey. J Adolesc Health 2010;**46**:592-599.

63. Gureje, O., Kola, L., Uwakwe, R., et al. The profile and risks of suicidal behaviours in the Nigerian Survey of Mental Health and Well Being. Psychol Med 2007;**37**:821-830.

64. Knauper, BC., CF, Schwarz, N., Bruce, ML., Keesler, RC. Improving the accuracy of major depression age of onset reports in the US National Comorbidity Survey. Int J Methods Psychiatr Res 1999;**8**(1):39-48

65. Brewin, CR., Andrews, B., Botlib, IH. Psychopathology and early experience: a reappraisal of retrospective reports. Psychol Bull 1993;**113**:82-98

66. Hardt, J., Rutter, M. Validity of adult retrospective reports of adverse childhood experiences: a review of the evidence. J Child Psychol Psychiatry 2004;**45**:260-273.

For peer review only

67. Dube, SR., Williamson, DF., Thompson, T., Felitti, VJ, Anda, RF. Assessing the reliability of retrospective reports of adverse childhood experiences amond adult HMO members attending a primary care clinic. Child Abuse Negl 2004;**28**(7):729-737.

68. Yancura, LA., Aldwin, CM. (2009). Stability and change in retrospective reports of childhood experiences over a 5-year period: Findings from the David Longitudinal Study. Psychol Aging 2009;**24**(3):715-721

69. Wilsnack, S.C., Wonderlich, S.A., Kristjanson, A.F., et al. (2002). Self reports of forgetting and remembering childhood sexual abuse in a nationally representative sample of US women. Child Abuse Negl 2002;**26**:139-147.

For peer review only

Association between childhood adversities and suicidality

Table 1: Descriptive Characteristics (N= 4351)

		N
Mean Age (yrs) (SE)	37.0 (0.26)	
**Age categories (yrs)**		
18 – 29	39.1%	1701
30 – 39	22.1%	962
40 - 49	18.1%	788
≥ 50	20.7%	901
**Sex ~~Gender~~**		
Male	46.3%	2015
Female	53.7%	2336
**Race**		
Black	76.2%	3315
Coloured	10.4%	453
White	10.0%	435
Indian/Asian	3.4%	148
**Married**	50.1%	2180
**Location**		
Rural	38.4%	1671
Urban	61.6%	2680
**Education**		
None	6.8 %	296
Grade 1-7	19.1%	831
Grade 8-11	35.4%	1540
Matric	23.5%	1022
Matric +	15.3%	666
**Employed**	31.0%	1349
**Income Category** (Rands), (mean SD)		
0	13.7%	596
1 - 2500	29.5%	1284
2501 – 5000	15.4%	670
5001 – 10 000	19.6%	853
≥ 10001	21.8%	949
**Province**		
Eastern Cape	13.1%	570
Free State	6.2%	270
Guateng	23.0%	1001
Kwazulu Natal	19.5%	848
Limpopo	10.5%	457
Mpumalanga	6.6%	287
Northern Cape	1.9%	83
North West	8.3%	361
Western Cape	11.1%	483

For peer review only

Association between childhood adversities and suicidality

Table 2: Prevalence of childhood adversities and suicidal behaviour in South Africa

[%[b] (S.E.)]

	Total Sample		Total Sample		Suicidal Ideators		Suicidal Ideators	
	With Attempt	No attempt	With Ideation	No ideation	With Plan	No plan	With Attempt	No attempt
Physical Abuse	24.9 (4.6)	12.2 (0.8)	21.1 (2.5)	11.8 (0.7)	24.3 (4.6)	27.9 (4.0)	24.9 (4.6)	24.5 (3.6)
Sexual Abuse	2.1 (1.2)	0.1 (0.0)	0.7 (0.4)	0.1 (0.0)	1.6 (0.9)	0.0 (0.0)	2.1 (1.2)	0.0 (0.0)
Parent Died	11.6 (2.4)	11.3 (0.6)	13.9 (2.3)	11.3 (0.6)	12.2 (2.4)	16.1 (4.2)	11.6 (2.4)	15.6 (3.8)
Parent Divorced	14.2 (3.8)	4.8 (0.4)	7.9 (1.6)	4.7 (0.4)	9.7 (2.6)	9.2 (3.7)	14.2 (3.8)	6.7 (2.9)
Other Parent Loss	2.1 (1.2)	2.2 (0.4)	3.9 (1.2)	2.1 (0.4)	1.1 (0.6)	3.0 (1.4)	2.1 (1.2)	2.7 (1.3)
Family Violence	4.3 (1.5)	3.0 (0.3)	4.1 (0.9)	2.9 (0.3)	4.7 (1.5)	6.3 (1.8)	4.3 (1.5)	4.5 (1.4)
Physical Illness	5.0 (2.3)	2.5 (0.3)	4.0 (1.2)	2.4 (0.3)	4.4 (1.8)	4.7 (1.8)	5.0 (2.3)	4.3 (1.6)
Financial Adversity	6.1 (2.4)	5.6 (0.5)	4.1 (0.9)	5.8 (0.5)	6.0 (2.1)	3.3 (1.5)	6.1 (2.4)	2.9 (1.0)
1	35.4 (4.2)	23.4 (1.0)	35.9 (2.8)	22.7 (0.9)	32.9 (4.0)	41.7 (5.2)	35.4 (4.2)	40.5 (4.5)
2+	15.4 (3.4)	8.6 (0.5)	10.8 (1.7)	8.6 (0.5)	14.1 (3.2)	13.2 (3.3)	15.4 (3.4)	9.6 (2.3)
a	(140)	(107309)	(394)	(112243)	(171)	(1976)	(140)	(2212)

[a] Number of cases with the outcome variable; N represents the number of person years.
[b] % represents the percentage of people with the adversity among the cases with the outcome variable indicated in the column header. For example: the first cell is the % of those with physical abuse among those with attempts.

For peer review only

Association between childhood adversities and suicidality

Table 3: Multivariate and Bivariate models for associations between childhood adversities and lifetime suicidality[1]

	LT Attempts in total sample[b]				Ideators among total sample[c]				Suicidal Ideators with LT plans [d]				Suicidal Ideators with LT attempts [e]			
	Multivariate		Bivariate		Multivariate		Bivariate		Multivariate		Bivariate		Multivariate		Bivariate	
	OR(95% CI)	Chi-square	OR(95% CI)	Chi-square	OR(95% CI)	Chi-square	OR(95% CI)	Chi-square	OR(95% CI)	Chi-square	OR(95% CI)	Chi-square	OR(95% CI)	Chi-square	OR(95% CI)	Chi-square
Physical Abuse	2.0* (1.2-3.3)*	7.4(0.006)*	2.0* (1.2-3.2)*	7.3(0.007)*	1.7* (1.3-2.3)*	15.2(<.001)*	1.7* (1.3-2.3)*	16.7(<.001)*	0.6 (0.3-1.4)	1.3(0.25)	0.7 (0.3-1.4)	1.2(0.26)	1.0 (0.5-2.3)	0.0(0.93)	1.1 (0.5-2.5)	0.1(0.81)
Sexual Abuse	7.6* (2.0-29.9)*	8.9(0.003)*	7.9* (1.9-32.1)*	8.6(0.003)*	2.6 (0.6-10.6)	1.8(0.18)	3.0 (0.7-12.2)	2.5(0.11)	---	---	---	---	---	---	---	---
Parent Died	1.1 (0.6-1.8)	0.1(0.78)	1.1 (0.7-1.7)	0.1(0.76)	1.4 (0.9-2.1)	2.7(0.10)	1.3 (0.9-1.9)	2.0(0.16)	0.7 (0.3-1.7)	0.6(0.45)	0.8 (0.4-1.9)	0.3(0.62)	0.8 (0.5-1.5)	0.4(0.52)	0.8 (0.4-1.5)	0.4(0.53)
Parent Divorced	2.7* (1.5-5.0)*	10.8(0.001)*	2.8* (1.5-5.2)*	11.4(<.001)*	1.6* (1.0-2.4)*	4.3(0.038)*	1.5 (1.0-2.3)	3.7(0.05)	0.9 (0.3-3.3)	0.0(0.88)	1.2 (0.4-3.8)	0.1(0.78)	3.1* (1.2-8.6)*	5.2(0.023)*	3.0* (1.1-8.0)*	4.9(0.027)*
Other Parent Loss	1.0 (0.3-3.3)	0.0(0.95)	0.9 (0.3-2.8)	0.1(0.81)	1.7 (1.0-3.0)	3.6(0.06)	1.6 (0.9-2.7)	2.9(0.09)	0.4 (0.1-2.6)	0.9(0.34)	0.5 (0.1-2.7)	0.7(0.41)	2.0 (0.2-17.3)	0.4(0.51)	2.5 (0.6-11.0)	1.5(0.22)
Family Violence	0.7 (0.3-1.7)	0.6(0.42)	1.0 (0.4-2.2)	0.0(0.98)	0.8 (0.5-1.4)	0.5(0.47)	1.1 (0.6-1.8)	0.0(0.83)	1.0 (0.4-2.4)	0.0(0.97)	0.8 (0.4-2.0)	0.2(0.68)	2.4 (0.9-6.3)	3.5(0.06)	2.2 (0.9-5.5)	2.9(0.09)
Physical Illness	1.1 (0.4-3.5)	0.1(0.81)	1.5 (0.6-4.1)	0.7(0.39)	1.2 (0.6-2.3)	0.2(0.63)	1.3 (0.7-2.4)	0.7(0.42)	0.8 (0.2-3.1)	0.1(0.71)	0.9 (0.2-3.5)	0.0(0.86)	1.2 (0.3-3.9)	0.1(0.80)	1.2 (0.4-4.0)	0.1(0.77)
Financial Adversity	1.0 (0.4-2.7)	0.0(0.94)	1.2 (0.5-2.8)	0.1(0.73)	0.6 (0.4-1.1)	3.0(0.08)	0.7 (0.4-1.2)	1.4(0.23)	2.4 (0.7-8.4)	1.9(0.17)	1.9 (0.6-6.8)	1.1(0.29)	2.1 (0.7-6.0)	2.1(0.15)	2.0 (0.7-6.3)	1.6(0.21)

*Significant at the .05 level, two-sided test
LT: lifetime

[1] Assessed in Part 2 sample due to having part 2 controls. Controls for the model include int (1-5 intervals), and also include significant variables from demographic and parent psychopathology models, details in the following footnotes.

[b]Models controls for int(1-5 intervals), demographics (sex, age, time-varying education), interaction between intervals(13-19,20-29,30+) and age, education. For parent psychopathology, controlling for number of parental disorders (dummies for 1, 2+ disorders).

[c]Models controls for int(1-5 intervals), demographics (sex, age, time-varying education), interaction between int intervals(13-19,20-29,30+) and age, education. For parent psychopathology, controlling for number of parental disorders (dummies for 1, 2+ disorders).

[d]Models controls for int(1-5 intervals), demographics (sex, age, time-varying education), interaction between int intervals(13-19,20-29,30+) and age, education. For parent psychopathology, controlling for types of parental disorders (6 dummies).

[e]Models controls for int(1-5 intervals), demographics (sex, age, time-varying education), interaction between int intervals(13-19,20-29,30+) and age, education. Parent psychopathology not controlled for due to insignificance in previous models.

For peer review only - http://bmjopen.bmj.com/site/about/guidelines.xhtml

Table 4: Associations between number of childhood adversities and lifetime suicidality[1]

	LT Attempts in total sample[b]		Ideators among total sample[c]		Ideators with LT plans [d]		Ideators with LT attempts [e]	
**Number of child adversities**	**OR(95% CI)**	**Chi-square**	**OR(95% CI)**	**Chi-square**	**OR(95% CI)**	**Chi-square**	**OR(95% CI)**	**Chi-square**
1	1.9* (1.3-2.8)*		1.8* (1.5-2.3)*		0.5 (0.3-1.0)		0.9 (0.5-1.7)	
2+	2.1* (1.2-3.8)*	14.3(<.001)*	1.4* (1.0-2.0)*	28.3(<.001)*	1.1 (0.3-3.3)	4.5(0.10)	2.7* (1.3-5.9)*	8.3(0.016)*

*Significant at the .05 level, two-sided test
LT: lifetime

[1] Assessed in Part 2 sample due to having part 2 controls. Controls for the model include int (1-5 intervals), and also include significant variables from demographic and parent psychopathology models, details in the following footnotes.
[b]Models controls for int(1-5 intervals), demographics (sex, age, time-varying education), interaction between int intervals(13-19,20-29,30+) and age, education. For parent psychopathology, controlling for number of parental disorders (dummies for 1, 2+ disorders).
[c]Models controls for int(1-5 intervals), demographics (sex, age, time-varying education), interaction between int intervals(13-19,20-29,30+) and age, education. For parent psychopathology, controlling for number of parental disorders (dummies for 1, 2+ disorders).
[d]Models controls for int(1-5 intervals), demographics (sex, age, time-varying education), interaction between int intervals(13-19,20-29,30+) and age, education. For parent psychopathology, controlling for types of parental disorders (6 dummies).
[e]Models controls for int(1-5 intervals), demographics (sex, age, time-varying education), interaction between int intervals(13-19,20-29,30+) and age, education. Parent psychopathology not controlled for due to insignificance in previous models.

Association between childhood adversities and suicidality

Table 5: Final multivariate model for associations between childhood adversities and lifetime suicidality[1]

	LT Attempts in total sample[b]		Ideators among total sample[c]		Ideators with LT plans[d]		Ideators with LT attempts[e]	
	OR(95% CI)	Chi-square	OR(95% CI)	Chi-square	OR(95% CI)	Chi-square	OR(95% CI)	Chi-square
Physical Abuse	2.2* (1.3-3.8)*	8.9(0.003)*	2.1* (1.6-2.8)*	25.4(<.001)*	0.4* (0.2-1.0)*	4.3(0.038)*	0.8 (0.3-2.1)	0.3(0.60)
Sexual Abuse	9.3* (2.5-35.2)*	11.2(<.001)*	3.7 (0.9-15.9)	3.3(0.07)	---	---	---	---
Parent Died	1.2 (0.7-2.3)	0.4(0.51)	1.7* (1.1-2.6)*	6.6(0.010)*	0.4 (0.1-1.3)	2.2(0.14)	0.6 (0.3-1.1)	2.8(0.10)
Parent Divorced	3.1* (1.7-5.6)*	14.5(<.001)*	1.9* (1.2-3.0)*	8.1(0.004)*	0.7 (0.2-2.3)	0.4(0.51)	2.4 (0.9-6.4)	3.0(0.08)
Other Parent Loss	1.1 (0.3-4.3)	0.0(0.87)	2.1* (1.3-3.6)*	8.3(0.004)*	0.3 (0.0-2.0)	1.8(0.18)	1.3 (0.1-13.3)	0.1(0.79)
Family Violence	0.9 (0.3-2.3)	0.1(0.76)	1.1 (0.6-2.3)	0.2(0.69)	0.4 (0.1-1.8)	1.6(0.20)	1.2 (0.4-4.1)	0.1(0.76)
Physical Illness	1.4 (0.4-5.3)	0.2(0.63)	1.6 (0.7-3.3)	1.4(0.24)	0.6 (0.1-2.5)	0.5(0.46)	0.9 (0.2-3.3)	0.0(0.85)
Financial Adversity	1.3 (0.4-3.7)	0.2(0.65)	0.9 (0.4-1.7)	0.1(0.71)	1.6 (0.4-6.0)	0.6(0.44)	1.4 (0.5-4.3)	0.4(0.52)
group significance test for all types		29.4(<.001)*		43.0(<.001)*		833.9(<.001)*		11.5(0.18)
significance test for difference between types		13.1(0.07)		9.2(0.24)		805.7(<.001)*		11.8(0.11)
2+ adversities	0.7 (0.2-1.8)	0.7(0.41)	0.5* (0.3-0.9)*	4.9(0.028)*	4.7 (0.8-29.2)	2.9(0.09)	2.9 (0.8-10.6)	2.7(0.10)

*Significant at the .05 level, two-sided test
LT: lifetime

[1] Assessed in Part 2 sample due to having part 2 controls. Controls for the model include int (1-5 intervals), and also include significant variables from demographic and parent psychopathology models, details in the following footnotes.

[b]Models controls for int(1-5 intervals), demographics (sex, age, time-varying education), interaction between int intervals(13-19,20-29,30+) and age, education. For parent psychopathology, controlling for number of parental disorders (dummies for 1, 2+ disorders).

[c]Models controls for int(1-5 intervals), demographics (sex, age, time-varying education), interaction between int intervals(13-19,20-29,30+) and age, education. For parent psychopathology, controlling for number of parental disorders (dummies for 1, 2+ disorders).

[d]Models controls for int(1-5 intervals), demographics (sex, age, time-varying education), interaction between int intervals(13-19,20-29,30+) and age, education. For parent psychopathology, controlling for types of parental disorders (6 dummies).

[e]Models controls for int(1-5 intervals), demographics (sex, age, time-varying education), interaction between int intervals(13-19,20-29,30+) and age, education. Parent psychopathology not controlled for due to insignificance in previous models.

For peer review only - http://bmjopen.bmj.com/site/about/guidelines.xhtml

Figure 1: Schematic representation

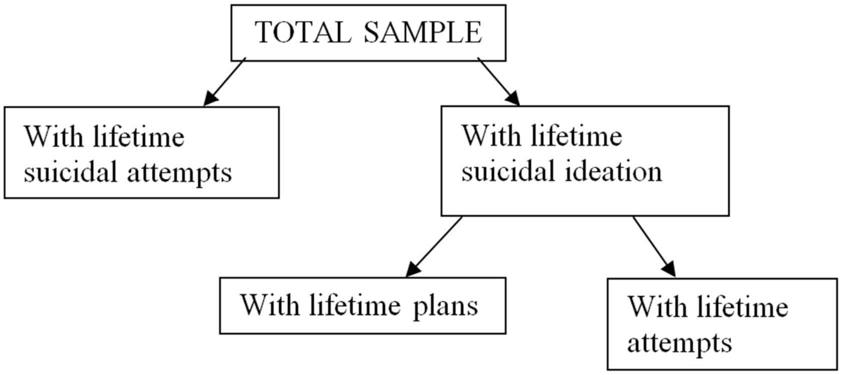

Schematic representation
301x142mm (96 x 96 DPI)

For peer review only

STROBE Statement—checklist of items that should be included in reports of observational studies

	Item No	Recommendation
**Title and abstract**	1	(*a*) Indicate the study's design with a commonly used term in the title or the abstract
		(*b*) Provide in the abstract an informative and balanced summary of what was done and what was found
**Introduction**		
Background/rationale	2	Explain the scientific background and rationale for the investigation being reported
Objectives	3	State specific objectives, including any prespecified hypotheses
**Methods**		
Study design	4	Present key elements of study design early in the paper
Setting	5	Describe the setting, locations, and relevant dates, including periods of recruitment, exposure, follow-up, and data collection
Participants	6	(*a*) *Cohort study*—Give the eligibility criteria, and the sources and methods of selection of participants. Describe methods of follow-up<br>*Case-control study*—Give the eligibility criteria, and the sources and methods of case ascertainment and control selection. Give the rationale for the choice of cases and controls<br>*Cross-sectional study*—Give the eligibility criteria, and the sources and methods of selection of participants
		(*b*) *Cohort study*—For matched studies, give matching criteria and number of exposed and unexposed<br>*Case-control study*—For matched studies, give matching criteria and the number of controls per case
Variables	7	Clearly define all outcomes, exposures, predictors, potential confounders, and effect modifiers. Give diagnostic criteria, if applicable
Data sources/ measurement	8*	For each variable of interest, give sources of data and details of methods of assessment (measurement). Describe comparability of assessment methods if there is more than one group
Bias	9	Describe any efforts to address potential sources of bias
Study size	10	Explain how the study size was arrived at
Quantitative variables	11	Explain how quantitative variables were handled in the analyses. If applicable, describe which groupings were chosen and why
Statistical methods	12	(*a*) Describe all statistical methods, including those used to control for confounding
		(*b*) Describe any methods used to examine subgroups and interactions
		(*c*) Explain how missing data were addressed
		(*d*) *Cohort study*—If applicable, explain how loss to follow-up was addressed<br>*Case-control study*—If applicable, explain how matching of cases and controls was addressed<br>*Cross-sectional study*—If applicable, describe analytical methods taking account of sampling strategy
		(*e*) Describe any sensitivity analyses

Continued on next page

For peer review only

Results		
Participants	13*	(a) Report numbers of individuals at each stage of study—eg numbers potentially eligible, examined for eligibility, confirmed eligible, included in the study, completing follow-up, and analysed
		(b) Give reasons for non-participation at each stage
		(c) Consider use of a flow diagram
Descriptive data	14*	(a) Give characteristics of study participants (eg demographic, clinical, social) and information on exposures and potential confounders
		(b) Indicate number of participants with missing data for each variable of interest
		(c) *Cohort study*—Summarise follow-up time (eg, average and total amount)
Outcome data	15*	*Cohort study*—Report numbers of outcome events or summary measures over time
		Case-control study—Report numbers in each exposure category, or summary measures of exposure
		Cross-sectional study—Report numbers of outcome events or summary measures
Main results	16	(*a*) Give unadjusted estimates and, if applicable, confounder-adjusted estimates and their precision (eg, 95% confidence interval). Make clear which confounders were adjusted for and why they were included
		(*b*) Report category boundaries when continuous variables were categorized
		(*c*) If relevant, consider translating estimates of relative risk into absolute risk for a meaningful time period
Other analyses	17	Report other analyses done—eg analyses of subgroups and interactions, and sensitivity analyses
**Discussion**		
Key results	18	Summarise key results with reference to study objectives
Limitations	19	Discuss limitations of the study, taking into account sources of potential bias or imprecision. Discuss both direction and magnitude of any potential bias
Interpretation	20	Give a cautious overall interpretation of results considering objectives, limitations, multiplicity of analyses, results from similar studies, and other relevant evidence
Generalisability	21	Discuss the generalisability (external validity) of the study results
**Other information**		
Funding	22	Give the source of funding and the role of the funders for the present study and, if applicable, for the original study on which the present article is based

*Give information separately for cases and controls in case-control studies and, if applicable, for exposed and unexposed groups in cohort and cross-sectional studies.

Note: An Explanation and Elaboration article discusses each checklist item and gives methodological background and published examples of transparent reporting. The STROBE checklist is best used in conjunction with this article (freely available on the Web sites of PLoS Medicine at http://www.plosmedicine.org/, Annals of Internal Medicine at http://www.annals.org/, and Epidemiology at http://www.epidem.com/). Information on the STROBE Initiative is available at www.strobe-statement.org.

For peer review only

BMJ Open

Association between childhood adversities and long-term suicidality among South Africans from the results of the South African Stress and Health Study: a cross-sectional study

Journal:	*BMJ Open*
Manuscript ID:	bmjopen-2013-004644.R2
Article Type:	Research
Date Submitted by the Author:	28-Apr-2014

Complete List of Authors:	Bruwer, Belinda; Stellenbosch University, Psychiatry<br>Govender, Ravi; Stellenbosch University, Psychiatry<br>Bishop, Melanie; Stellenbosch University, Psychiatry<br>Williams, David; Harvard University, Human Development and Health<br>Stein, Dan; University of Cape Town, Psychiatry and mental health<br>Seedat, Soraya; Stellenbosch University, Psychiatry

**Primary Subject Heading**:	Mental health
Secondary Subject Heading:	Mental health
Keywords:	MENTAL HEALTH, Child & adolescent psychiatry < PSYCHIATRY, Adult psychiatry < PSYCHIATRY, PSYCHIATRY, Suicide & self-harm < PSYCHIATRY

SCHOLARONE™
Manuscripts

Association between childhood adversities and long-term suicidality among South Africans from the results of the South African Stress and Health Study: a cross-sectional study

Belinda Bruwer*[a], Ravi Govender[a], Melanie Bishop[a], David R Williams[b] Dan J Stein[c], Soraya Seedat[a]

[a] Department of Psychiatry, Stellenbosch University, PO Box 19063, Tygerberg, 7505, Republic of South Africa.

[b] Department of Society, Human Development and Health, Harvard School of Public Health, and Department of African and African American Studies, Harvard University. Department of Society, Human Development and Health, 677 Huntington Avenue, 6th floor, Boston, MA 02115, United States of America

[c] Department of Psychiatry and Mental Health, University of Cape Town, Groote Schuur Hospital (J2), Anzio Road, Observatory, 7925, Cape Town, Republic of South Africa

*Corresponding author: Department of Psychiatry, University of Stellenbosch, PO Box 19063, Tygerberg, 7505, Republic of South Africa.

Tel nr: +27219404467. Fax nr: +27219404543, bbruwer@sun.ac.za

Keywords: Childhood adversities, suicidal ideation, suicidal attempts, suicide, suicidal behaviour

Word count: 5349

For peer review only

Association between childhood adversities and suicidality

ABSTRACT

Objective:

Suicide and suicidal behaviours are significant public health problems and a leading cause of death worldwide and in South Africa. We examined the association between childhood adversities and suicidal behaviour over the life course.

Methods:

A national probability sample of 4,351 South African adult participants (aged 18 years and older) in the South African Stress and Health (SASH) study was interviewed, as part of the World Mental Health Survey initiative. Respondents provided socio-demographic and diagnostic information, as well as an account of suicide-related thoughts and behaviours. Outcomes were defined as suicide attempts and suicidal ideation in the total sample, and suicide plans and attempts among ideators. Childhood adversities included physical abuse, sexual abuse, parental death, parental divorce, other parental loss, family violence, physical illness and financial adversity. The association between suicidality and childhood adversities was examined using discrete-time survival models.

Results:

More than a third of respondents with suicidal behaviour experienced at least 1 childhood adversity, with physical abuse, parental death and parental divorce the most prevalent adversities. Physical abuse, sexual abuse and parental divorce were identified as significant risk markers for lifetime suicide attempts, while physical abuse and parental divorce were significantly correlated with suicidal ideation. Two or more childhood adversities were associated with a 2-fold higher risk of lifetime suicide attempts. Sexual abuse (OR=9.3, childhood, parental divorce (OR=3.1)

For peer review only

and physical abuse (OR=2.2) had the strongest associations with lifetime suicide attempts. The effect of childhood adversities on suicidal tendencies varied over the *life course.* For example, sexual abuse was significantly associated with suicide attempts during childhood and teen years, but not during young and later adulthood.

Conclusions:

Childhood adversities, especially sexual abuse, physical abuse and parental divorce are important risk factors for the onset and persistence of suicidal behaviour, with this risk greatest in childhood and adolescence. The risk for suicidal behaviour was greatest in childhood and adolescence. Suicidal risk in childhood and adolescence was significantly associated with the following childhood adversities: sexual abuse, physical abuse and parental divorce.

Keywords: Childhood adversities, suicidal ideation, suicidal attempts

For peer review only

Association between childhood adversities and suicidality

Article Summary

Strengths and limitations

- Recall bias might have impacted on the accuracy of recall of childhood adversities.
- In view of the cross-sectional design, more detailed, temporal information on childhood adversities and suicidal incidents was not obtained.
- Variables such as culture, ethnicity and mental status at the time of the interview may have influenced the recall and reporting of suicidal behaviour. Stigma associated with mental health problems may have also played a role in the reporting suicidal tendencies.
- We do not assess for self-mutilating behavior. The importance of discriminating suicidal behaviour from non-suicidal self-mutilation cannot be underestimated. The CIDI instrument which was used in this study is a lay-administered instrument which does not include an assessment of several key DSM-IV diagnoses (such as bipolar disorder and psychosis), are associated with elevated rates of suicidality. As a result, some participants with suicidality may have not have been diagnosed with a disorder.
- This study represents the first investigation among South Africans of a wide range of childhood adversities and their impact on the onset and persistence of suicidality over the life course.

For peer review only

Association between childhood adversities and suicidality

INTRODUCTION

Suicide and suicidal behaviour are significant public health problems. Suicide is one of the leading causes of death worldwide with almost 1 million people committing suicide each year [1]. This figure is likely to grow to approximately 1.2 million suicides in 2020 [2]. In South Africa, the annual rate of suicide is high [3, 4] mirroring international trends [5]. So, too, are rates of suicidal behaviour with an estimated prevalence of 9.1% for lifetime suicidal ideation and 2.9% for suicide attempts among South Africans according to the South African Stress and Health Survey (SASH) [6].

Despite the enormity of the problem, the aetiology of suicidal behaviour is not fully understood. There are controversies in the literature regarding prior psychiatric disorder and risk for suicide attempts. While some authors have argued that pre-existing disorder is an important risk factor (7-11], others have argued that suicide attempts are not neccessarily associated with prior psychopathology [12]. Genetic factors also play an important role in suicidal behaviour [13-16]. While there is stronger evidence pointing towards environmental or experiential factors [17, 18] such as exposure to childhood adversities [19-28]. Recent multi-level country data from the World Mental Health Surveys (WMHS) initiative has allowed for cross-national comparisons of suicidality. The WMHS investigated the association between childhood adversities and suicidal behaviour [20], the persistence of suicidality over time, and the extent to which associations between childhood trauma and suicidality changed over the life course. The WMHS found a dose-response relationship between the number of adversities and suicidal behaviour. Sexual abuse and physical abuse were the strongest risk factors for both the onset and persistence of suicidal behaviours, with the risk for suicidality greatest during childhood (age 4-12 years) and adolescence (age 13-19 years) [20].

For peer review only

Association between childhood adversities and suicidality

Numerous studies have examined the link between childhood sexual abuse and suicidality [29-41]. All of these authors have found that exposure to childhood sexual abuse increases the risk for mental disorders, including suicidality. Furthermore, the majority of studies that have focused on the link between childhood physical abuse and suicidality have found that exposure to childhood physical abuse increases the risk for suicidality [42, 43]. There also appears to be an association between the number of childhood adversities experienced and the later suicidal behaviour [21, 23, 24, 44, 45].

Exposure to early life stress is prevalent among South Africans. In one sample of South African rural youth, the prevalence of physical and sexual abuse was shown to be very high with 94.4% of males exposed to physical abuse and 39.1% of females to sexual abuse [46]. More than a quarter of adults who were interviewed endorsed exposure to childhood adversity (parental death, parental separation or parental divorce) in the SASH study [47]. Significantly more females were prone to be victims of domestic violence than men [47]. Women also reported twice as many suicidal attempts than the male participants in the SASH study [9].

Objective

We report in more detail on data from a South African dataset gathered as part of the World Mental Health Surveys, which allowed for comparison with data from the overall cross-national sample. This data are particularly interesting as South Africa is a middle income African country with high rates of violent trauma exposure. The present study aimed to examine the relationship between the type and frequency of childhood adversity exposure to suicidal behaviour over the life trajectory of South Africans, given that there are no published nationally representative data that may be useful in informing both clinical practice and policy.

For peer review only

Association between childhood adversities and suicidality

METHODS

Sample

Data for the SASH Study were collected between January 2002 and June 2004. WMH surveys were carried out in 21 countries which included Nigeria and South Africa [48]. For detailed information on study methods see Williams et al. (2004) [48]. The research protocol for the SASH study was approved by the Human Subjects Committee of the University of Michigan, by Harvard Medical School ethics committee and by a single project assurance of compliance from the Medical University of South Africa (MEDUNSA), and by the National Institute of Mental Health. It was a national probability sample of 4,351 South African adults (persons aged 18 years and older) living in households or in hostel accommodation. All racial and ethnic groups were represented, with the sample selected using a three-stage probability sample design. The response rate was 85.5%.

Sampling approach

Sampling was divided into three stages. Primary sampling units was selected during the first stage, which was based on the 2001 SA census Enumeration Areas (EAs). The second stage involved sampling of household units within clusters selected in each EA. South Africans in both urban and rural areas were sampled. Sampled residences were stratified into 10 diverse housing categories: Rural–commercial, agricultural, rural traditional subsistence areas, African townships, informal urban or peri-urban shack areas, Coloured townships, Indian townships, general metropolitan residential areas, general large metropolitan residential areas, and domestic servant accommodation in urban areas. During the third stage, one adult respondent in each sampled housing unit was selected. A total of 5089 households was selected. Field interviews were conducted with 4433 (87.1%) of designated respondents. Based on quality control, 4351

For peer review only

interviews were retained for use in the analysis. There were no differences in response rates across the four designated racial groups (white, Coloured [mixed racial origin], Indian, black). According to the 2001 Census statistics, 79.% people in South Africa are Black African, 8.9% are coloured, 9.6% are white, and 2.5% are Indian/Asian [49].

Diagnostic Interview

SASH used version 3 of the World Health Organization Composite Diagnostic Interview (WHO CIDI) [50]. Interviewers were trained within a one week period and conducted the interviews in seven different languages, namely English, Afrikaans, Zulu, Xhosa, Northern Sotho, Southern Sotho, and Tswana. Translations of the CIDI into several native South African languages were conducted in accordance with WHO requirements. Multilingual and bilingual expert panels conducted the back-translations [51, 52]. Informed consent was obtained from participants after a complete description of the study was provided. Respondents provided socio-demographic and diagnostic information, as well as an account of suicidal behaviours during the interviews. The core diagnostic assessment of mental disorders included anxiety disorders (panic disorder, agoraphobia, social phobia, generalized anxiety disorder, post-traumatic stress disorder), mood disorders (major depressive disorder, dysthymia), substance use disorders (alcohol abuse, alcohol dependence, drug abuse, drug dependence) and intermittent explosive disorder [53, 54].

Suicidal behaviour

The CIDI 3.0 module on suicidal behaviour was used to assess the age-of-first-onset, age of most recent episode, and lifetime occurrence of suicidal ideation, suicide plans and suicide attempts. Suicidal ideation, suicide plans and suicide attempts was assessed with questions such as “Have you ever seriously thought about committing suicide?”, “Have you ever made a plan for committing suicide?”, and “Have you ever attempted suicide?”, respectively. Ideators only

For peer review only

proceeded to answer questions about plans ("Have you ever made a plan for committing suicide?") and attempts ("Have you ever attempted suicide?"). Information on the age of first occurrence of the three main outcomes was obtained. To get a better understanding of the progression from ideation to attempt, the outcomes considered in this study were: suicide attempts in the total sample; suicide ideation in the total sample; suicide plans among ideators; suicide attempts among ideators with a plan (planned attempts), and suicide attempts among ideators in the absence of a plan (unplanned or impulsive attempts).

Childhood adversities

Physical abuse, sexual abuse, parental death, parental divorce, other parental loss, family violence, physical illness and financial adversity were the various childhood adversities assessed. Biological and non-biological parents were included in measures of parental death, divorce or other parental loss. Financial adversities were assessed with questions on whether the family had insufficient funds to pay for basic necessities. Questions about repeated fondling, attempted rape or rape were asked to assess for sexual abuse. This comprised the following "The next 2 questions are about sexual assault: (i) The first is about rape. We define this as someone either having sexual intercourse with you or penetrating your body with a finger or object when you did not want them to, either by threatening you or using force, or when you were so young that you didn't know what was happening. Did this ever happen to you?", and (ii)"Other than rape, were you ever sexually assaulted or molested?". A modified version of the Conflict Tactics Scale (CTS2) was used to assess family violence and physical abuse [55]. Respondents were classified as having experienced *physical abuse* when they indicated that, when they were growing up, their father or mother (includes biological, step, or adoptive parents) slapped, hit, pushed, grabbed, shoved, or threw something at them, or that they were beaten as a child by the persons

For peer review only

who raised them. Family violence was assessed as present when respondents indicated that they (i) "were often hit, shoved, pushed, grabbed, or slapped while growing up" *or* (ii) "witnessed physical fights at home, like when your father beat up your mother?" A standard chronic conditions checklist assessed for life-threatening physical illnesses in childhood [56].

Data analysis

All data analyses were processed and analysed centrally by a team of statisticians at the Harvard School of Public Health (Boston, USA) using the SAS version 9.1.3 software package. Discrete-time survival analysis with time-varying covariates was used to study the risk factors of lifetime suicide ideation, plans and attempts. Data were weighted to adjust for the stratified multistage sample design, differential probability of selection within households as a function of household size and clustering of data, and differential non-response. Overall, percentages were weighted to adjust for differences in selection probabilities, differential non-response, oversampling of cases, and residual differences on sociodemographic variables between the sample and the population [48, 57]. A post-stratification weight was also used to make the sample distribution comparable, for age, sex, and province, with the population distribution in the 2001 South African census. Both weighted and geographic clustering of data were taken into account in the data analyses by using a jackknife repeated replications simulation method implemented in SAS macro 14. The survival coefficients were exponentiated and are reported below in the form of odds ratios.

The association between suicidality and childhood adversity was examined using discrete-time survival models with the analysis unit being person-years. Bivariate analyses (considering one adversity at a time) and multivariate analyses (considering all adversities simultaneously) were conducted. Two types of multivariate models were tested: multivariate additive models (simultaneously considering all childhood adversities) and multivariate interactive models (with

For peer review only

Association between childhood adversities and suicidality

number and type of childhood adversities experienced by each respondent included as dummy variables). The analysis also examined interactions between the life stage (13-19 years, 20-29 years, 30+ years) of respondents and each childhood adversity, as well as the influence each adversity had on early-, middle- and later- onset suicidality. Analyses were conducted using SUDAAN version 8.1 to adjust for clustering and weighting. Odds ratios (ORs) with a 95% confidence interval (CIs) are reported. Wald X^2- tests were used to examine multivariate significance. Associations between adversities and suicide outcomes were adjusted for sex, age, educational level, marital status, interactions between demographic variables, life course, lifetime mental disorders and parental psychopathology. Analyses also examined the influence of respondents' lifetime mental disorders on suicidality, as well as interactions between sex and each childhood adversity. Statistical significance using two-sided tests was set at $p < .05$ [20]. Based on an N of 4000 (alpha of 0.05, 2 sided significance), the study was adequately powered (.99), to detect an OR of 2.0 of a continuously distributed normalized predictor and a 10% prevalence of suicidal behaviour.

RESULTS

Demographic details

In the sample, (n = 4351), there were slightly more female (53.7%) than male respondents. There were more black (76.2%) than coloured (10.4%), white (10%), and Indian/Asian (3.4%) respondents. Furthermore, half of the sample was married and most were unemployed (69.2%), had less than 12 years of education (62.7%) and lived in an urban area (59.7%) (see Table 1).

Prevalence of childhood adversities among the total sample

Figure 1 provides a schematic representation of the suicidality data reported in the sections which follow. In the total sample, 35.4% of participants with one adversity had a suicide attempt,

For peer review only

compared with 23.4% with one adversity who had not made an attempt. Physical abuse (24.9%), parental divorce (14.2%) and parental death (11.6%) were most prevalent among those suicide attempters. Among those exposed to one childhood adversity, without a suicide attempt, the two most prevalent adversities reported were physical abuse (12.2%) and parental death (11.3%). In the total sample 15.4% of participants exposed to two or more adversities had a suicide attempt. In contrast, 8.6% of participants exposed to two or more adversities had not made an attempt (Table 2).

Prevalence of childhood adversities among suicidal ideators in the total sample

In the sample as a whole, 35.9% of those with one adversity had suicidal ideation compared with 22 .7% of those with one adversity who had no ideation. The most prevalent adversities associated with suicidal ideation were physical abuse (21.1%), parental death (13.9%), and parental divorce (7.9%). Among those without suicidal ideation, physical abuse (11.8%) and parental death (11.3%) were the most commonly endorsed childhood adversities. Of those who endorsed two or more childhood adversities, 10.8% reported suicidal ideation and 8.6% did not (Table 2). In summary, the most prevalent childhood adversities reported among the total sample with/without suicidal ideation were firstly, physical abuse and secondly, the death of a parent.

Prevalence of suicide attempts in the total sample

In the total sample, 24.9% of those with childhood physical abuse had attempted suicide while 12.2% of respondents with no physical abuse had no attempt. Of those exposed to parental divorce, 14.2% had attempted suicide and 4.8% had made no attempt. The second most prevalent childhood adversity was parental death with 11.6% of those with parental death attempting suicide and 11.3% of those with parental death with no attempts (Table 2).

For peer review only

Association between childhood adversities and suicidality

Prevalence of childhood adversities among suicidal ideators

With/without a plan

Among suicidal ideators with a plan, 32.9% had experienced one childhood adversity. Among ideators with no plan, 41.7% had one childhood adversity. Among ideators with a plan, the following were the most prevalent childhood adversities: physical abuse (24.3%), parental death (12.2%), and parental divorce (9.7%). Among ideators without a plan, 27.9% endorsed physical abuse, 16.1% parental death, and 9.2% parental divorce (see Table 2). In both groups (ideators with and without a plan), physical abuse was the most prevalent childhood adversity, followed by parental death and parental divorce.

With or without an attempt

Among suicidal ideators who had attempted suicide, 35.4% were exposed to one childhood adversity and 15.4% were exposed to two or more childhood adversities. In the group of ideators who had made an attempt, 24.9% had experienced physical abuse, 14.2% parental divorce, and 11.6% parental death (Table 2). 40.5% of those with one adversity, and 9.6% of those exposed to two or more adversities were suicidal ideators with no attempts. In this group, the most prevalent adversities were physical abuse (24.5%), parental death (15.6%) and parental divorce (6.7%) reported (Table 2).

Among all ideators (with/without a plan, with/without an attempt), the most prevalent childhood adversity was physical abuse, followed by parental death and parental divorce. Of note, in the group of ideators with an attempted suicide parental divorce was more prevalent than parental death.

Bivariate and multivariate results: Type of childhood adversity

For peer review only

Association between childhood adversities and suicidality

Bivariate and multivariate analyses were performed to examine the associations between the different childhood adversities (physical abuse, sexual abuse, parental death, parental divorce, other parental loss, family violence, physical illness, financial adversity) and lifetime suicidal ideation, plans and attempts.

In the total sample, bivariate and multivariate analysis revealed significant associations between (i) sexual abuse (bivariate: OR=7.9, p=0.003; multivariate: OR=7.6, p=0.003), (ii) physical abuse (OR 2, p=0.007; OR 2.0, p=0.006) and (iii) parental divorce (OR 2.8, p<.001; OR 2.7, p=0.001), and lifetime suicide attempts. Among ideators in the sample, physical abuse (OR=1.7, p<.001; OR=1.7, p<.001) was significantly associated with suicidal ideation. Multivariate analyses revealed an additional association with suicidal ideation, namely parental divorce (OR = 1.6, p=0.038). The relationship between childhood adversities and lifetime plans was not statistically significant. However, a significant association was found between parental divorce and lifetime suicidal attempts among ideators (OR=3.0, p<.001; OR=3.1, p=0.023) (Table 3).

Findings from multivariate analysis, therefore, confirm findings of bivariate analysis for all groups, except for ideators. Among ideators bivariate analysis revealed a significant relationship between physical abuse and suicidal ideation. This was confirmed in multivariate analysis where the association between parental divorce and suicidal ideation was significant for the whole sample.

Bivariate associations between the number of adversities and lifetime suicidality

The relationship between the number of childhood adversities and lifetime suicidal ideation, plans and attempts was further examined. There was a significant relationship between the number of childhood adversities and lifetime suicide attempts. Two or more childhood

For peer review only - http://bmjopen.bmj.com/site/about/guidelines.xhtml

Association between childhood adversities and suicidality

adversities were associated with a 2-fold higher risk of lifetime suicide attempts in the total sample (OR=2.1, p<.001). A significant relationship was also established between one, as well as two or more adversities with ideators in the total sample. Among ideators, no significant association was found between the number of childhood adversities and lifetime plans. A significant relationship was found between two or more adversities and lifetime attempts among ideators (OR=2.7, p=0.016), indicating a more than 2-fold higher risk of lifetime suicide attempts in this group (Table 4).

Multivariate associations between number of childhood adversities and lifetime suicidality

In the final multivariate model which included 2 or more adversities as a predictor variable, sexual abuse (OR=9.3, p<.001), childhood physical abuse (OR=2.2, p=0.003) and parental divorce (OR=3.1, p<.001) retained significant associations with lifetime suicide attempts in the total sample. Physical abuse (OR=2.1, p<.001), parental death (OR=1.7, p=0.010), parental divorce (OR=1.9, p=0.004) and other parental loss (OR = 2.1, p=0.004) were significant predictors of suicidal ideation (Table 5a) The same findings emerged after controlling for mental disorders, with the exception that sexual abuse was also significantly associated with suicidal ideation (Table 5b).Physical abuse was associated with a lower odds of lifetime suicide plans among ideators (OR = 0.4, p=0.038) (Table 5a). There were no significant associations between childhood adversities and lifetime attempts among those with suicidal ideation. The findings remain unchanged after controlling for mental disorders (Table 5b).

Associations between the types of childhood adversity and lifetime suicidality over the life course

Multivariate analyses were performed to examine the association between the types of childhood adversity and lifetime suicidal ideation, plans and attempts during childhood years (age 4- 12),

For peer review only - http://bmjopen.bmj.com/site/about/guidelines.xhtml

teenage years (age 13-19), young adulthood (age 20-29) and later adulthood (30 years and older) (see Web table 1).

Childhood years (4-12). Sexual abuse (OR=61.6, CI=4.5-841.0, p=0.002) in early childhood (4-12 years of age) was significantly associated with lifetime suicide attempts in the total sample (OR = 61.6, CI=4.5-841.0, p=0.002). Both sexual abuse (OR=34.8, CI= 3.1-392.6, p=0.003) and physical abuse (OR=3.7, CI=1.0-13.4, p=0.041) were associated with a higher risk for suicidal ideation among the total sample. No significant associations were found between any of the childhood adversities and lifetime plans in the group of ideators. Among those with suicidal ideation, parental death (OR=2.2, CI=1.1-4.3, p=0.021) was significantly associated with suicide attempts in childhood years.

Teen years (13-19). Sexual abuse (OR=20.3, CI=2.0-210.2, p=0.010), physical abuse (OR=3.7, CI=1.5-9.2, p=0.004), and parental divorce (OR=4.6, CI=1.7-12.1, p=0.002) were significantly associated with suicide attempts in the total sample of teenagers. Physical abuse (OR=3.6, CI=2.2-5.9, p<.001) and parental death (OR=2.2, CI=1.1-4.3, p=0.021) significantly increased the risk for suicidal ideation among the total group of teens. Physical illness (OR=9.9, CI=1.8-54.0, p=0.007) significantly increased the risk of suicidal plans in teens with suicidal ideation. Suicide attempts among teens with suicidal ideation was significantly predicted by parental divorce (OR=4.3, CI=1.1-17.0, p=0.035).

Young adulthood (20-29). None of the childhood adversities were significantly associated with lifetime suicide attempts during young adulthood in the sample overall. An explanation could be that suicide attempts spike earlier and later in life among South Africans, contributing to the lack

For peer review only

Association between childhood adversities and suicidality

of significance. Parental loss other than parental death was significantly associated with suicidal ideation (OR=2.9, CI=1.2-7.4, p=0.019).

Later adulthood (≥ 30). Childhood physical abuse (OR=2.2, CI=1.0-4.8, p=0.035) was significantly predictive of suicidal attempts. The likelihood of suicidal ideation significantly increased in later adulthood if parental loss other than parental death (OR=5.1, CI=2.1-12.1, p<.001) or physical illness had been present during childhood (OR=4.3, CI=1.1-15.9, p=0.028). No significant relationship was found between any of the childhood adversities and lifetime plans in the group of ideators although a significant relationship was found between two or more adversities and lifetime plans among those who were ideators (OR=44.5, CI=2.5-779.1, p<0.008). None of the childhood adversities were significantly associated with suicide attempts among ideators in this age group.

DISCUSSION

Rates of childhood adversities and suicidal behaviours were both high among South Africans, with more than a third of respondents in the total sample who attempted suicide experiencing one childhood adversity, and 15.4% experiencing two or more adversities. Overall, physical abuse, sexual abuse, parental divorce and physical illness were far more prevalent in those with a suicide attempt than in those without. The most prevalent childhood adversities endorsed overall were physical abuse followed by parental death. Physical abuse, parental divorce and death of a parent were also the most prevalent adversities experienced in those with a suicide attempt as well as in those with suicidal ideation. These findings are somewhat dissimilar to other country samples; for example in the 21 countries that participated in the WMHS, physical abuse (29.3%), family violence (24.8%) and neglect (19.3%) were the most prevalent childhood adversities among those with a lifetime suicide attempt, while physical abuse (20.6%), family violence (17.6%) and death

For peer review only

of a parent (14.2%) were most often reported among participants with lifetime suicidal ideation [20]. Cross-nationally, it would appear that physical abuse is the commonest childhood adversity associated with lifetime suicide attempts and ideation [20].

The estimate lifetime prevalence of 2.9% for attempted suicide among South Africans is close to the rates of 4.6% and 4.1% reported for general and Black populations respectively in USA. In addition the 9.1% estimated prevalence of suicide ideation is comparable with previous estimates from studies in South African clinical samples. Joe et al. (2008b) reported for the first time on the rates of suicide ideation, plan and attempts among the different ethnic groups, in data from the SASH study [6]. Overall, the results suggest that people in SA engage in suicidal thought and behaviours at levels nearly comparable with those of Western nations.

When examining suicidal behaviour risk in the context of childhood adversity, sexual abuse, physical abuse and parental divorce emerged as significant risk factors for lifetime suicide attempts in the total sample. Furthermore, physical abuse and parental divorce were significant risk factors for suicidal ideation in the total sample. After adjusting for mental illness, sexual abuse was also a significant risk factor for suicidal ideation. Parental divorce emerged as a significant risk factor among ideators with lifetime suicide. These findings are largely consistent with the data from the overall cross-national WMHS, which found that physical and sexual abuse significantly increased the likelihood of suicidal ideation and attempts, while neglect was a risk factor for suicidal behaviour in multivariate additive analyses [20].

Of the adversities implicated, sexual and physical abuse were more significant risk factors than other adversities, highlighting the fact that intrusive and aggressive experiences in childhood may have more devastating and longer lasting effects [58]. This may be due to the extreme

For peer review only

Association between childhood adversities and suicidality

powerlessness and loss of control that such abuse causes, or to physically aggressive assaults resulting in the devaluation of one's body and consequent susceptibility to self harm [28]. In a country with high rates of sexual and physical abuse [46] this is particularly concerning. The impact of parental divorce on suicidality supports previous findings that parental divorce, if accompanied by other adversities such as childhood abuse, increases the risk of suicidal behaviour [59].

We also found that exposure to *two or more childhood adversities* significantly increased the risk of suicide attempts among ideators. This confirms earlier work showing exposure to multiple childhood adversities increases the risk of suicidal behaviour [21, 23, 24, 60, 61]. Bruffaerts et al (2010) found a sub-additive effect with regards to the onset of suicidal behaviour when considering multiple adversities [20]. Thus, the impact of multiple adversities was not equal to the sum of the odds ratios of individual adversities. In the overall WMHS analysis exposure to multiple childhood adversities had a significant effect on the persistence of suicide when considering every additional childhood adversity exposed to, however in the current study it was not possible to stratify the number of adversities beyond two or more adversities (i.e. into more than 2 categories) given the relatively small number of cases in the sample overall with non-fatal suicidal behaviour. Physical abuse, parental death, parental loss other than through death, and parental divorce emerged as independent risk factors for suicidal ideation in the total sample. Moreover, the effects of childhood adversities on suicidal tendencies tended to differ over the *life course*. Consistent with nationally representative data in WMHS, childhood adversities were associated with the highest risk of suicide attempts in childhood, with a decrease in risk in adolescence and young adulthood, followed by an increase in risk again during later adulthood [20].

For peer review only

Association between childhood adversities and suicidality

In *childhood*, sexual abuse was significantly associated with lifetime suicide attempts in the total sample, while sexual and physical abuse were significantly associated with suicidal ideation. Among suicidal ideators, parental death was significantly associated with lifetime suicide attempts. Exposure to childhood sexual abuse, physical abuse or parental divorce significantly increased suicide attempts during *teenage years*, while physical abuse and parental death were associated with suicidal ideation in teens. Among teen suicidal ideators, physical illness was significantly associated with suicidal plans, while parental divorce was associated with suicide attempts. These findings emphasize the need to focus suicide prevention strategies at youth in particular. In *young adulthood,* parental loss other than the death of a parent was significantly associated with suicidal ideation in the total sample. Interestingly, childhood physical abuse was identified as a significant risk factor for suicidal attempts in *later adulthood*, while childhood physical illness and parental loss other than the death of a parent significantly increased the risk for ideation.

Similar to findings from SASH, childhood sexual abuse emerged as a particularly robust risk factor for suicide attempts in younger participants in the WMH cross-national analysis, with a 10.9 times higher odds of suicide attempts in children, a 6.1 times higher likelihood in adolescents and a 2.9-fold risk in young adults who were exposed [20]. This is in keeping with Enns hypothesis that sexual abuse results in suicidal behaviour at a younger age [21]. Consistent with other studies, childhood physical and sexual abuse, in particular, emerged as risk factors for the emergence and persistence of suicidal behaviour, especially in adolescence. Loss of a parent, physical ill-health and family violence has also been found to be associated with persistence of suicidality [20, 28, 58]. These findings extend previous work done in other developing countries that have found childhood adversities to be a significant risk factor for suicidality [20, 62-64].

For peer review only

Association between childhood adversities and suicidality

Limitations

The following limitations need to be highlighted. First, recall bias might have impacted on the accuracy of recall of childhood adversities. This said, participants were asked questions about childhood adversities in sequence which may have facilitated more accurate recall [65]. Systematic reviews have also found that recall of past experiences can be accurate and can provide valuable data [66, 67]. Thus, there is evidence to support the validity of accurate recall of childhood adversities [67]. Further, studies have shown that responses to questions on childhood adversities, similar to those asked in the SASH study, generally remain stable over time [68, 69]. We recommend that future studies examine ethnicity in relation to adversity and suicidal outcomes. Second, in view of the cross-sectional design, more detailed, temporal information on childhood adversities and suicidal incidents was not obtained. Third, variables such as culture, ethnicity and mental status at the time of the interview may have influenced the recall and reporting of suicidal behaviour. It is possible that response bias may have been particularly skewed to disenfranchised South Africans (e.g. poor, young, urban an black respondents), who may have been too afraid to divulge information on suicidality. Stigma associated with mental health problems may have also played a role in the reporting suicidal tendencies. Thus, participants' mental health status, ethnicity, culture and generational factors may have also contributed to the under-reporting of suicidality. It is possible that individuals reporting childhood adversities may have also been more likely to report suicidal behaviour, while those not reporting childhood adversities may have underreported suicidality. Stigma and mental health status (e.g. depressed persons may be more inclined to report suicidality and more likely to remember negative childhood experiences) may also be contributory factors. In addition, some participants

For peer review only

may have been afraid to report suicidal behaviours. The role of ethnicity, culture and generational factors may have also contributed to the under-reporting of suicidality. Overall, it is much more likely that adversities and suicidality were under-reported rather than over-reported [9, 20, 67, 70]. Fourth, we do not assess for self-mutilating behavior. The importance of discriminating suicidal behaviour from non-suicidal self-mutilation cannot be underestimated. Fifth, the survey was conducted in adults living in households and hostel quarters thus the findings are not generalizable to homeless and institutionalized persons who were not included in the survey. Sixth, the CIDI instrument which was used in this study is a lay-administered instrument which does not include an assessment of several key DSM-IV diagnoses (such as bipolar disorder and psychosis), are associated with elevated rates of suicidality. As a result, some participants with suicidality may have not have been diagnosed with a disorder. Furthermore, in view of the large confidence intervals and small sample sizes for some of these analyses caution is required in drawing conclusions. In addition, we did not control for other unmeasured causes of childhood adversities and suicidality, or protective (resiliency) factors that may have contributed to the associations observed in these data. Both other risk and resiliency factors may have contributed to both the prevalence of non-fatal suicidal behaviours and to the associations with different forms of childhood adversity and warrant further investigation. Lastly, it is important to point out that these data were collected approximately 10 years ago. Notwithstanding these limitations, this study represents the first investigation among South Africans of a wide range of childhood adversities and their impact on the onset and persistence of suicidality over the life course.

Conclusions

Childhood adversities especially sexual abuse, physical abuse and parental divorce are associated with the onset and persistence of suicidal behaviour with the risk greatest in children and

For peer review only

adolescents. Public health efforts aimed at prevention of early childhood sexual and physical abuse, in particular, may have a significant impact on reducing suicidality over the life course and improving mental health outcomes.

For peer review only

Association between childhood adversities and suicidality

Funding: This work was funded by the South African Department of Health and the University of Michigan; Medical Research Council of South Africa

Contributorship:

Belinda Bruwer: Data interpretation, drafting manuscript, final approval of manuscript submitted for publication, ensuring that questions related to the accuracy of the work are appropriately resolved.

Ravi Govender: Data interpretation, drafting manuscript, final approval of manuscript submitted for publication, ensuring that questions related to the accuracy of the work are appropriately resolved

Melanie Bishop: Data interpretation, revising the manuscript, final approval of manuscript submitted for publication, ensuring that questions related to the accuracy of the work are appropriately resolved

David Williams: Substantial contributions to the conception or design of the work, data acquisition, data analysis and interpretation, critically revising of the manuscript, final approval of the version to be published; and accountability for all aspects of the work

Dan Stein: Substantial contributions to the conception or design of the work, data acquisition, data analysis and interpretation, critically revising of the manuscript, final approval of the version to be published; and accountability for all aspects of the work

Soraya Seedat: Substantial contributions to the conception or design of the work, data acquisition, data analysis and interpretation, critically revising of the manuscript, final approval of the version to be published; and accountability for all aspects of the work

Competing Interests:

For peer review only

Association between childhood adversities and suicidality

This study was funded by NIH. Additional funding was received from the South African Department of Health and the University of Michigan. D.J.S. and S.S. are also supported by the Medical Research Council of South Africa. D.J.S. has received research grants and / or consultancy honoraria from AstraZeneca, Eli Lilly, GlaxoSmithKline, Lundbeck, Orion, Pfizer, Pharmacia, Roche, Servier, Solvay, Sumitomo and Wyeth. S.S. has received research grants or travel sponsorship from AstraZeneca, Eli Lilly, GlaxoSmithKline, Lundbeck and Servier. S.S. is supported by the by the South African Research Chairs Initiative of the Department of Science and Technology and the National Research Foundation. B.B has received congress sponsorship from Janssen-Cilag.

Data Sharing Statement: No additional data are available

For peer review only

REFERENCES

Association between childhood adversities and suicidality

1. World Health Organization. Suicide Prevention (SUPRE). Geneva, Switzerland. 2007. http://www.who.int/mental_helath/prevention/suicide/suicideprevention/en/)

2. Murray, C.L., Lopez, A.D. The global burden of disease: a comprehensive assessment of mortality and disability from disease, injuries and risk factors in 1990 and projected to 2020. Cambridge, MA: Harvard University Press, 1996.

3. Burrows, S., Laflamme, L. Pattern analysis of suicide mortality surveillance data in urban South Africa. Suicide and Life- Threatening Behaviour 2008;**38**:209-220.

4. Meel, B.I. Epidemiology of suicide by hanging in Transkei. South Africa. Am J Forensic Med Pathol. 2006;**27**:75-78

5. Flisher, A.J., Liang, H., Laubscher, R. Suicide trends in South Africa, 1968-90. Scand J Public Health 2004;**32**:411-418.

6. Joe, S., Stein, DJ., Seedat, S., et al. non-fatal suicidal behavior among South Africans: Results from the South Africa Stress and Health Study. Social Psychiatry Epidemiology 2008;**43**(6):454–461.doi:10.1007/s00127-008-0348-7.

7. Beautrais, A.L., Joyce, P/R/. & Mulder, R.T. (1996). Risk factors for serious suicide attempts among youth aged 13 through 24 years. J Am Acad Child Adolesc Psychiatry 1996;**35**(9):1174-1182.

8. Harrison, EC, Barraclough, B. (1997). Suicide as an outcome for mental disorders: A meta-analysis. Br J Psychiatry 1997;**170**:205-228

9. Joe, S., Stein, D.J., Seedat, S., et al. Prevalence and correlates of non-fatal suicidal behaviour among South Africans. Br J Psychiatry 2008;**192**:310-311.

For peer review only

10. Nock, M.K., Borges, G., Bromet, E.J., et al. Suicide and Suicidal Behaviour. Epidemiologic Reviews 2008;**30**:133-154.

11. Nock, M.K., Borges, G., Bromet, E.J., et al. (2008b). Cross-national prevalence and risk factors for suicidal ideation, plans and attempts. *British Journal of Psychiatry*, 192, 98-105.

12. Nock, M.K., Hwang, I., Sampson, N.A., et al. Cross-national analysis of the associations among mental disorders and suicidal behaviour: Findings from the WHO World Mental Health Surveys. PLos Medicine 2009;**6**(8).e1000123.

13. Bondy, B., Buettner, A., Zill, P. Genetics of suicide. Molecular Psychiatry 2006;**11**:336-351.

14. Kohli, M.A., Salyakina, D., Pfennig, A., et al. Association of genetic variants in the neurotrophic receptor encoding gene NTRK2 and a lifetime history of suicide attempts in depressed patients. Arch Gen Psychiatry 2010;**67**:348-59.

15. Roy, A., Hu, X-Z., Janal, M.N., et al. Interaction between childhood trauma and serotonin transporter gene variation and suicide. Neuropsychopharmacology 2007;**32**:2046–2052

16. Risch, N., Herrell, R., Lehner, T., et al. Interaction between the serotonin transporter gene (5-HTTLPR), stressful life events, and the risk of depression: A meta-analysis. JAMA 2009;**301**:2462–2471.

17. Borges, G., Benjet, C., Medina-Mora, M.E., et al. Traumatic events and suicide related outcomes among Mexico City adolescents. J Child Psychol Psychiatry 2008;**6**:654-666.Weissman MM, Bland

18. RC, Canino GJ, Greenwald S, Hwu HG, Joyce PR, et al. (1999) Prevalence of suicide ideation and suicide attempts in nine countries. *Psychology Med, 29*: 9–17.

For peer review only

19. Brodsky, BS & Stanley, B. Adverse childhood experiences and suicidal behaviour. Psychiatry Clinical Northern America 2008;**31**:223-235

20. Bruffaerts, R., Demyttenaere, K., Borges, G., et al. Childhood adversities as risk factors for onset and persistence of suicidal beahviour. Br J Psychiatry 2010;**197**:20-27.

21. Enns, M.W., Cox, B.J., Afifi, T.O., et al. Childhood adversities and risk for suicidal ideation and attempts: a longitudinal population-based study. Psychological Medicine 2006;**36**:1769-1778.

22. Johnson, J.G., Cohen, P., Gould, M.S., et al. Childhood adversities, interpersonal difficulties, and risk for suicide attempts during late adolescence and early adulthood. Arch Gen Psychiatry 2002;**59**:741-749.

23. Dube, S.R., Anda, R.F., Felitti, V.J., et al. Childhood abuse, household dysfunction, and the risk of attempted suicide throughout the life span: Findings from the Adverse Childhood Experiences Study. JAMA 2001;**286**:3089-3096.

24. Afifi, T.O., Enns, M.W., Cox, B.J., et al. Population attributable fractions of psychiatric disorders and suicide ideation and attempts associated with adverse childhood experiences. Am J Public Health 2008;**98**:946-952.

25. Burke, A.K., Galfalvy, H., Everett, B., et al. Effect of exposure to suicidal behavior on suicide attempt in a high-risk sample of offspring of depressed parents. J Am Acad Child Adolesc Psychiatry 2010;**49**:114-121.

26. Labonte, B., Suderman, M., Maussion, G., et al. Genome-wide epigenetic regulation by early-life trauma. Arch Gen Psychiatry 2012;**69**(7):722-731.Doi:10.1001/archgenpsychiatry.2011.2287

For peer review only

27. Lipschitz, D.S., Winegar, R.K., Nicolaou, A.L., et al. (1999). Perceived abuse and neglect as risk factors for suicidal behaviour in adolescent inpatients. *The Journal of Nervous and Mental Disease*, 187, 32-39.

28. Ystgaard, M., Hestetun, I., Loeb, M., et al. Is there a specific relationship between childhood sexual and physical abuse and repeated suicidal behaviour? Child Abuse Neg 2004;**28**:863-875

29. Boudewyn, A., & Liem, J. Childhood sexual abuse as a presecutor to depression and self-destructive behavior in adulthood. J Trauma Stress 1995;**8**:445-459.

30. Brown, J., Cohen, P., Johnson, J.G., et al. Childhood abuse and neglect: Specificity of effects on adolescent and young adult depression and suicidality. J Am Acad Child Adolesc Psychiatry 1999;**38**:1490-1496.

31. Bryant, S.L., & Range, L.M. Suicidality in college women who were sexually and physically abused and physically punished by parents. Violence Vict 1995;**10**:195-201.

32. Davidson, J.R.T., Hughes, D.C., George, L.K., et al. The association of sexual assault and attempted suicide within the community. Arch Gen Psychiatry 1996;**53**:550-555

33. Fergusson, D.M., & Mullen, P.E. Childhood Sexual abuse – An evidence based perspective. Sage, CA: Thousand Oaks, 1999.

34. Finkelhor, D. Early and long-term effects of child sexual abuse: An update. Professional Psychology: Research & Practice 1990;**21**(5):325-330.

35. Finkelhor, D., & Hashima, P.Y. (2001). The victimization of children and youth: A comprehensive overview. In S.O. White (Ed.) Handbook of youth and justice. The Plenum series in crime and justice. Dordrecht: Plenum, 2001:49-78.

For peer review only

36. Holmes, W.C., & Slap, G.B. Sexual abuse of boys: Definition, prevalence, correlates, sequelae, and management. JAMA: JAMA 1998;**280**(21):1855-1862

37. Kendall-Tackett, K.A., Williams, L.M., & Finkelhor, D. Impact of sexual abuse on children: A review and synthesis of recent empirical studies. Psychol Bull 1993;**113**(1):164-180.

38. Martin, G. Reported family dynamics, sexual abuse, and suicidal behaviors in community adolescents. Arch Suicide Res 1996;**2**:183-195.

39. Peters, D.K., & Range, L.M. Childhood sexual abuse and current suicidality in college women and men. Child Abuse Negl 1995;**19**:335-341.

40. Putman, F.W. Ten-year research update review: Child sexual abuse. J Am Acad Child Adolesc Psychiatry 2003;**42**(3):269-278

41. Stepakoff, S. Effects of sexual victimization on suicidal ideation and behaviour in US college women. Suicide and Life-Threatening Behavior 1998;**28**:107-126.

42. Malinosky-Rummel, R., & Hansen, D.J. Long-term consequences of childhood physical abuse. Psychol Bull 1993;**144**:68-79

43. Silverman, A.B., Reinherz, H., & Giaconia, R.M. The long-term sequelae of child and adolescent abuse: A longitudinal community study. Child Abuse Negl 1996;**20**:709-723

44. Chapman, D.P., Whitfield, C.L., Felitti, V.J., et al. Adverse childhood experiences and the risk of depression in adulthood. J Affect Disord 2004;**82**:217-225

45. Dube, S.R., Felitti, V.J., Dong, M., et al. Childhood abuse, neglect, and household dysfunction and the risk of illicit drug use: The adverse childhood experiences study. Pediatricc 2003;**111**:564-572.

For peer review only

46. Jewkes, R.K., Dunkle, K., Nduna, M., et al. Associations between childhood adversity and depression, substance abuse and HIV and HSV2 incident infections in rural South African youth. Child Abuse Negl 2010;**34**:833-841.

47. Seedat, S., Stein, D.J., Jackson, P.B., et al. Life stress and mental disorders in the South African Stress and Health study. South African Medical Journal 2009a;**99:**375-382.

48. Williams, D.R., Herman, A., Kessler, R.C., et al. The South Africa Stress and Health Study: Rationale and Design. Metab Brain Dis 2004;**19**(1/2):135-147.

49. Statistics South Africa. Census 2001: Census in Brief. Pretoria: Statistics South Africa. 2001. Available from http://www.statssa.gov.za/census01/html/CInBrief/CIB2001.pdf (Accessed January 2014)

50. Kessler, R.C., Üstün, T.B. The World Mental Health (WMH) Survey Initiative Version of the World Health Organization (WHO) Composite International Diagnostic Interview (CIDI). Int J Methods Psychiatr Res 2004;**13**:61-98.

51. Seedat, S., Stein, D.J., Herman, A., et al. Twelve-month treatment of Psychiatric disorders in South Africa Stress and Health Study (World Mental Health Survey Initiative). Psychiatric Epidemiology 2008;**38**:211-220.

52. Seedat, S., Williams, D.R., Herman, A., et al. Mental health service use among South Africans for mood, anxiety and substance use disorders. South African Medical Journal 2009b;**99**:346-352.

53. World Health Organization. World Health Organization Manual of the international statistical classification of diseases, injuries and causes of death, ninth revision. Geneva, Switzerland, 1992.

54. American Psychiatric Association. Diagnostic and statistical manual of mental disorders (DSM-IV), 4th Edition. Washington: American Psychiatric Association Press, 1994.

For peer review only

55. Straus MA. Measuring Intrafamily Conflict and Violence: The Conflict Tactics (CT) Scales. Journal of Marriage and Family 1979;**41(1**):75

56. Kessler, R.C., McLaughlin, K.A., Green, J.G. Childhood adversities and adult psychopathology in the WHO World Mental Health Surveys. Br J Psychiatry 2010;**197**:378-385.

57. Stein, D.J., Chiu, W.T., Hwang, I., et al. Cross-national analysis of the associations between traumatic events and suicidal behavior: Findings from the WHO World Mental Health Surveys. PloS ONE 2010;**5**(5):e10574.

58. Joiner Jr, T.E., Sachs-Ericsson, N.J., Wingate, L.R. Childhood physical and sexual abuse and lifetime number of suicide attempts: A persistent and theoretically important relationship. Behav Res Ther 2007;**45**:539-547.

59. Afifi, T.O., Boman, J., Fleisher, W., et al. The relationship between child abuse, parental divorce, and lifetime mental disorders and suicidality in a nationally representative adult sample. Child Abuse Negl 2009;**33**:139–147.

60. Bebbington, P.E., Cooper, C.C., Minot, S., et al. Suicide attempts, gender, and sexual abuse: data from the 2000 British Psychiatric Morbidity Survey. Am J Psychiatry 2009;**166**:1135-1140.

61. Molner, B, Buka, S, & Kessler, R. Child sexual abuse and subsequent psychopathology: results from the National Comorbidity Survey. American Journal Public Health 2001;**91**:753-760.

62. Borges, G., Angst, J., Nock, M.K., et al. Risk factors for the incidence and persistence of suicide related outcomes: a 10 year follow up study using the National Comorbidity Surveys. J Affect Disord 2008;**105**:25-33

For peer review only

63. Xing, X-Y., Tao, F-B., Wan, Y-H., et al. Family factors associated with suicide attempts among Chinese adolescent students: A national cross-sectional survey. J Adolesc Health 2010;**46**:592-599.

64. Gureje, O., Kola, L., Uwakwe, R., et al. The profile and risks of suicidal behaviours in the Nigerian Survey of Mental Health and Well Being. Psychol Med 2007;**37**:821-830.

65. Knauper, BC., CF, Schwarz, N., et al. Improving the accuracy of major depression age of onset reports in the US National Comorbidity Survey. Int J Methods Psychiatr Res 1999;**8**(1):39-48

66. Brewin, CR., Andrews, B., Botlib, IH. Psychopathology and early experience: a reappraisal of retrospective reports. Psychol Bull 1993;**113**:82-98

67. Hardt, J., Rutter, M. Validity of adult retrospective reports of adverse childhood experiences: a review of the evidence. J Child Psychol Psychiatry 2004;**45**:260-273.

68. Dube, SR., Williamson, DF., Thompson, T., et al. Assessing the reliability of retrospective reports of adverse childhood experiences amond adult HMO members attending a primary care clinic. Child Abuse Negl 2004;**28**(7):729-737.

69. Yancura, LA., Aldwin, CM. (2009). Stability and change in retrospective reports of childhood experiences over a 5-year period: Findings from the David Longitudinal Study. Psychol Aging 2009;**24**(3):715-721

70. Wilsnack, S.C., Wonderlich, S.A., Kristjanson, A.F., et al. (2002). Self reports of forgetting and remembering childhood sexual abuse in a nationally representative sample of US women. Child Abuse Negl 2002;**26**:139-147.

For peer review only

Figure Legend

Figure 1: Schematic Representation

For peer review only

Association between childhood adversities and suicidality

Table 1: Descriptive Characteristics (N= 4351)

		N
Mean Age (years) (SE)	37.0 (0.26)	
**Age categories (years)**		
18 – 29	39.1%	1701
30 – 39	22.1%	962
40 - 49	18.1%	788
≥ 50	20.7%	901
**Sex**		
Male	46.3%	2015
Female	53.7%	2336
**Race**		
Black	76.2%	3315
Coloured (mixed race)	10.4%	453
White	10.0%	435
Indian/Asian	3.4%	148
**Married**	50.1%	2180
**Location**		
Rural	38.4%	1671
Urban	61.6%	2680
**Education**		
None	6.8 %	296
Grade 1-7	19.1%	831
Grade 8-11	35.4%	1540
Matric	23.5%	1022
Matric +	15.3%	665.7
**Employed**	31.0%	1349
**Income Category** (Rands), (mean SD)		
0	13.7%	596
1 - 2500	29.5%	1284
2501 – 5000	15.4%	670
5001 – 10 000	19.6%	853
≥ 10001	21.8%	949
**Province**		
Eastern Cape	13.1%	570
Free State	6.2%	270
Gauteng	23.0%	1001
Kwazulu Natal	19.5%	848
Limpopo	10.5%	457
Mpumalanga	6.6%	287
Northern Cape	1.9%	83
North West	8.3%	361
Western Cape	11.1%	483

For peer review only

Association between childhood adversities and suicidality

Table 2: Prevalence of childhood adversities and suicidal behaviour in South Africa

[%[b] (S.E.)]

	Total Sample		Total Sample		Suicidal Ideators		Suicidal Ideators	
	With Attempt	No attempt	With Ideation	No ideation	With Plan	No plan	With Attempt	No attempt
Physical Abuse	24.9 (4.6)	12.2 (0.8)	21.1 (2.5)	11.8 (0.7)	24.3 (4.6)	27.9 (4.0)	24.9 (4.6)	24.5 (3.6)
Sexual Abuse	2.1 (1.2)	0.1 (0.0)	0.7 (0.4)	0.1 (0.0)	1.6 (0.9)	0.0 (0.0)	2.1 (1.2)	0.0 (0.0)
Parent Died	11.6 (2.4)	11.3 (0.6)	13.9 (2.3)	11.3 (0.6)	12.2 (2.4)	16.1 (4.2)	11.6 (2.4)	15.6 (3.8)
Parent Divorced	14.2 (3.8)	4.8 (0.4)	7.9 (1.6)	4.7 (0.4)	9.7 (2.6)	9.2 (3.7)	14.2 (3.8)	6.7 (2.9)
Other Parent Loss	2.1 (1.2)	2.2 (0.4)	3.9 (1.2)	2.1 (0.4)	1.1 (0.6)	3.0 (1.4)	2.1 (1.2)	2.7 (1.3)
Family Violence	4.3 (1.5)	3.0 (0.3)	4.1 (0.9)	2.9 (0.3)	4.7 (1.5)	6.3 (1.8)	4.3 (1.5)	4.5 (1.4)
Physical Illness	5.0 (2.3)	2.5 (0.3)	4.0 (1.2)	2.4 (0.3)	4.4 (1.8)	4.7 (1.8)	5.0 (2.3)	4.3 (1.6)
Financial Adversity	6.1 (2.4)	5.6 (0.5)	4.1 (0.9)	5.8 (0.5)	6.0 (2.1)	3.3 (1.5)	6.1 (2.4)	2.9 (1.0)
1	35.4 (4.2)	23.4 (1.0)	35.9 (2.8)	22.7 (0.9)	32.9 (4.0)	41.7 (5.2)	35.4 (4.2)	40.5 (4.5)
2+	15.4 (3.4)	8.6 (0.5)	10.8 (1.7)	8.6 (0.5)	14.1 (3.2)	13.2 (3.3)	15.4 (3.4)	9.6 (2.3)
a	(140)	(107309)	(394)	(112243)	(171)	(1976)	(140)	(2212)

[a] Number of cases with the outcome variable; N represents the number of person years.
[b] % represents the percentage of people with the adversity among the cases with the outcome variable indicated in the column header. For example: the first cell is the % of those with physical abuse among those with attempts.

For peer review only

Association between childhood adversities and suicidality

Table 3: Multivariate and Bivariate models for associations between childhood adversities and lifetime suicidality[1]

	LT Attempts in total sample[b]				Ideators among total sample[c]				Suicidal Ideators with LT plans [d]				Suicidal Ideators with LT attempts [e]			
	Multivariate		Bivariate		Multivariate		Bivariate		Multivariate		Bivariate		Multivariate		Bivariate	
	OR(95% CI)	Chi-square	OR(95% CI)	Chi-square	OR(95% CI)	Chi-square	OR(95% CI)	Chi-square	OR(95% CI)	Chi-square	OR(95% CI)	Chi-square	OR(95% CI)	Chi-square	OR(95% CI)	Chi-square
Physical Abuse	2.0* (1.2-3.3)*	7.4(0.006)*	2.0* (1.2-3.2)*	7.3(0.007)*	1.7* (1.3-2.3)*	15.2(<.001)*	1.7* (1.3-2.3)*	16.7(<.01)*	0.6 (0.3-1.4)	1.3(0.25)	0.7 (0.3-1.4)	1.2(0.26)	1.0 (0.5-2.3)	0.0(0.93)	1.1 (0.5-2.5)	0.1(0.81)
Sexual Abuse	7.6* (2.0-29.9)*	8.9(0.003)*	7.9* (1.9-32.1)*	8.6(0.003)*	2.6 (0.6-10.6)	1.8(0.18)	3.0 (0.7-12.2)	2.5(0.11)	---	---	---	---	---	---	---	---
Parent Died	1.1 (0.6-1.8)	0.1(0.78)	1.1 (0.7-1.7)	0.1(0.76)	1.4 (0.9-2.1)	2.7(0.10)	1.3 (0.9-1.9)	2.0(0.16)	0.7 (0.3-1.7)	0.6(0.45)	0.8 (0.4-1.9)	0.3(0.62)	0.8 (0.5-1.5)	0.4(0.52)	0.8 (0.4-1.5)	0.4(0.53)
Parent Divorced	2.7* (1.5-5.0)*	10.8(0.001)*	2.8* (1.5-5.2)*	11.4(<.001)*	1.6* (1.0-2.4)*	4.3(0.038)*	1.5 (1.0-2.3)	3.7(0.05)	0.9 (0.3-3.3)	0.0(0.88)	1.2 (0.4-3.8)	0.1(0.78)	3.1* (1.2-8.6)*	5.2(0.023)*	3.0* (1.1-8.0)*	4.9(0.027)*
Other Parent Loss	1.0 (0.3-3.3)	0.0(0.95)	0.9 (0.3-2.8)	0.1(0.81)	1.7 (1.0-3.0)	3.6(0.06)	1.6 (0.9-2.7)	2.9(0.09)	0.4 (0.1-2.6)	0.9(0.34)	0.5 (0.1-2.7)	0.7(0.41)	2.0 (0.2-17.3)	0.4(0.51)	2.5 (0.6-11.0)	1.5(0.22)
Family Violence	0.7 (0.3-1.7)	0.6(0.42)	1.0 (0.4-2.2)	0.0(0.98)	0.8 (0.5-1.4)	0.5(0.47)	1.1 (0.6-1.8)	0.0(0.83)	1.0 (0.4-2.4)	0.0(0.97)	0.8 (0.4-2.0)	0.2(0.68)	2.4 (0.9-6.3)	3.5(0.06)	2.2 (0.9-5.5)	2.9(0.09)
Physical Illness	1.1 (0.4-3.5)	0.1(0.81)	1.5 (0.6-4.1)	0.7(0.39)	1.2 (0.6-2.3)	0.2(0.63)	1.3 (0.7-2.4)	0.7(0.42)	0.8 (0.2-3.1)	0.1(0.71)	0.9 (0.2-3.5)	0.0(0.86)	1.2 (0.3-3.9)	0.1(0.80)	1.2 (0.4-4.0)	0.1(0.77)
Financial Adversity	1.0 (0.4-2.7)	0.0(0.94)	1.2 (0.5-2.8)	0.1(0.73)	0.6 (0.4-1.1)	3.0(0.08)	0.7 (0.4-1.2)	1.4(0.23)	2.4 (0.7-8.4)	1.9(0.17)	1.9 (0.6-6.8)	1.1(0.29)	2.1 (0.7-6.0)	2.1(0.15)	2.0 (0.7-6.3)	1.6(0.21)
Group Significant test of controls: demographic variables		403.8(<.001)*				1102.1 (<.001)*				12.0 (0.002)*						
Group significance of controls: interactions between demographics and intervals		816.6(<.001)*				1374.8 (<.001)*				159.9 (<.001)*						
Group significance of controls: demographics and interactions between demographics and intervals		8369.9(<.001)*				6190.0 (<.001)*				529.0(<.001)*						
Group significance of controls: parent psychopathology		12.0(0.003)*				16.7 (<.001)*										

Association between childhood adversities and suicidality

*Significant at the .05 level, two-sided test
LT: lifetime

[1] Assessed in Part 2 sample due to having part 2 controls. Controls for the model include intervals (1-5 intervals), and also include significant variables from demographic and parent psychopathology models, details in the following footnotes.

[b] Models controls for intervals (1-5 intervals), demographics (sex, age, time-varying education), interaction between intervals(13-19,20-29,30+) and age, education. For parent psychopathology, controlling for number of parental disorders (dummies for 1, 2+ disorders).

[c] Models controls for intervals (1-5 intervals), demographics (sex, age, time-varying education), interaction between intervals(13-19,20-29,30+) and age, education. For parent psychopathology, controlling for number of parental disorders (dummies for 1, 2+ disorders).

[d] Models controls for intervals (1-5 intervals), demographics (sex, age, time-varying education), interaction between intervals(13-19,20-29,30+) and age, education. For parent psychopathology, controlling for types of parental disorders (6 dummies).

[e] Models controls for intervals (1-5 intervals), demographics (sex, age, time-varying education), interaction between intervals(13-19,20-29,30+) and age, education. Parent psychopathology not controlled for due to insignificance in previous models.

For peer review only

Association between childhood adversities and suicidality

Table 4: Associations between number of childhood adversities and lifetime suicidality[1]

	LT Attempts in total sample[b]		Ideators among total sample[c]		Ideators with LT plans [d]		Ideators with LT attempts [e]	
**Number of child adversities**	**OR(95% CI)**	**Chi-square**	**OR(95% CI)**	**Chi-square**	**OR(95 % CI)**	**Chi-square**	**OR(95% CI)**	**Chi-square**
1	1.9* (1.3-2.8)*		1.8* (1.5-2.3)*		0.5 (0.3-1.0)		0.9 (0.5-1.7)	
2+	2.1* (1.2-3.8)*	14.3(<.001)*	1.4* (1.0-2.0)*	28.3(<.001)*	1.1 (0.3-3.3)	4.5(0.10)	2.7* (1.3-5.9)*	8.3(0.016) *
Group significant test of controls: demographic variables		538.4(<.001) *		1146.6(<.001) *		600.3(<.001)*		1943.4(<.0 01)*
Group significance of controls: interactions between demographics and intervals		859.8(<.001) *		1473.5(<.001) *		1389.0(<.00 1)*		1657.7(<.0 01)*
Group significance of controls: demographics and interactions between demographics and intervals		11496.7(<.0 01)*		7255.3(<.001) *		11233.3(<.0 01)*		6714.6(<.0 01)*
Group significance of controls: parent psychopathology		12.7(0.002)*		19.1(<.001)*		14.1(0.029) *		

*Significant at the .05 level, two-sided test
LT: lifetime

[1] Assessed in Part 2 sample due to having part 2 controls. Controls for the model include intervals (1-5 intervals), and also include significant variables from demographic and parent psychopathology models, details in the following footnotes.

[b]Models controls for intervals (1-5 intervals), demographics (sex, age, time-varying education), interaction between intervals(13-19,20-29,30+) and age, education. For parent psychopathology, controlling for number of parental disorders (dummies for 1, 2+ disorders).

[c]Models controls for intervals (1-5 intervals), demographics (sex, age, time-varying education), interaction between intervals(13-19,20-29,30+) and age, education. For parent psychopathology, controlling for number of parental disorders (dummies for 1, 2+ disorders).

[d]Models controls for intervals (1-5 intervals), demographics (sex, age, time-varying education), interaction between intervals(13-19,20-29,30+) and age, education. For parent psychopathology, controlling for types of parental disorders (6 dummies).

[e]Models controls for intervals (1-5 intervals), demographics (sex, age, time-varying education), interaction between intervals(13-19,20-29,30+) and age, education. Parent psychopathology not controlled for due to insignificance in previous models.

Table 5a: Final multivariate model for associations between childhood adversities and lifetime suicidality[1]

	LT Attempts in total sample[b]		Ideators among total sample[c]		Ideators with LT plans [d]		Ideators with LT attempts [e]	
	**OR(95% CI)**	**Chi-square**	**OR(95% CI)**	**Chi-square**	**OR(95% CI)**	**Chi-square**	**OR(95% CI)**	**Chi-square**
Physical Abuse	2.2* (1.3-3.8)*	8.9(0.003)*	2.1* (1.6-2.8)*	25.4(<.001) *	0.4* (0.2-1.0)*	4.3(0.038) *	0.8 (0.3-2.1)	0.3(0.60)
Sexual Abuse	9.3* (2.5-35.2)*	11.2(<.001)*	3.7 (0.9-15.9)	3.3(0.07)	---	---	---	---

For peer review only - http://bmjopen.bmj.com/site/about/guidelines.xhtml

Association between childhood adversities and suicidality

Parent Died	1.2 (0.7-2.3)	0.4(0.51)	1.7* (1.1-2.6)*	6.6(0.010)*	0.4 (0.1-1.3)	2.2(0.14)	0.6 (0.3-1.1)	2.8(0.10)
Parent Divorced	3.1* (1.7-5.6)*	14.5(<.001)*	1.9* (1.2-3.0)*	8.1(0.004)*	0.7 (0.2-2.3)	0.4(0.51)	2.4 (0.9-6.4)	3.0(0.08)
Other Parent Loss	1.1 (0.3-4.3)	0.0(0.87)	2.1* (1.3-3.6)*	8.3(0.004)*	0.3 (0.0-2.0)	1.8(0.18)	1.3 (0.1-13.3)	0.1(0.79)
Family Violence	0.9 (0.3-2.3)	0.1(0.76)	1.1 (0.6-2.3)	0.2(0.69)	0.4 (0.1-1.8)	1.6(0.20)	1.2 (0.4-4.1)	0.1(0.76)
Physical Illness	1.4 (0.4-5.3)	0.2(0.63)	1.6 (0.7-3.3)	1.4(0.24)	0.6 (0.1-2.5)	0.5(0.46)	0.9 (0.2-3.3)	0.0(0.85)
Financial Adversity	1.3 (0.4-3.7)	0.2(0.65)	0.9 (0.4-1.7)	0.1(0.71)	1.6 (0.4-6.0)	0.6(0.44)	1.4 (0.5-4.3)	0.4(0.52)
group significance test for all types		29.4(<.001)*		43.0(<.001)*		833.9(<.001)*		11.5(0.18)
significance test for difference between types		13.1(0.07)		9.2(0.24)		805.7(<.001)*		11.8(0.11)
2+ adversities	0.7 (0.2-1.8)	0.7(0.41)	0.5* (0.3-0.9)*	4.9(0.028)*	4.7 (0.8-29.2)	2.9(0.09)	2.9 (0.8-10.6)	2.7(0.10)
Group significant test of controls: demographic variables		414.6(<.001)*		1112.0(<.001)*		214.0(<.001)*		10.4(0.005)*
Group significance of controls: demographics and interactions between demographics and intervals		831.6(<.001)*		1405.7(<.001)*		1063.6(<.001)*		174.7(<.001)*
Group significance of controls: demographics and interactions between demographics and intervals		8596.4(<.001)*		6292.0(<.001)*		4268.0(<.001)*		532.5(<.001)*
Group significance of controls: parent psychopathology		11.6(0.003)*		15.4(<.001)*		15.0(0.020)*		

*Significant at the .05 level, two-sided test
LT: lifetime

[1] Assessed in Part 2 sample due to having part 2 controls. Controls for the model include intervals (1-5 intervals), and also include significant variables from demographic and parent psychopathology models, details in the following footnotes.

[b] Models controls for intervals (1-5 intervals), demographics (sex, age, time-varying education), interaction between intervals(13-19,20-29,30+) and age, education. For parent psychopathology, controlling for number of parental disorders (dummies for 1, 2+ disorders).

[c] Models controls for intervals (1-5 intervals), demographics (sex, age, time-varying education), interaction between intervals(13-19,20-29,30+) and age, education. For parent psychopathology, controlling for number of parental disorders (dummies for 1, 2+ disorders).

[d] Models controls for intervals (1-5 intervals), demographics (sex, age, time-varying education), interaction between intervals(13-19,20-29,30+) and age, education. For parent psychopathology, controlling for types of parental disorders (6 dummies).

[e] Models controls for intervals (1-5 intervals), demographics (sex, age, time-varying education), interaction between intervals(13-19,20-29,30+) and age, education. Parent psychopathology not controlled for due to insignificance in previous models.

For peer review only - http://bmjopen.bmj.com/site/about/guidelines.xhtml

Association between childhood adversities and suicidality

Table 5b: Final multivariate model for associations between childhood adversities and lifetime suicidality, controlling for mental disorders

	LT Attempts in total sample[b]		Ideators among total sample[c]		Ideators with LT plans[d]		Ideators with LT attempts[e]	
	OR(95% CI)	Chi-square	OR(95% CI)	Chi-square	OR(95% CI)	Chi-square	OR(95% CI)	Chi-square
Physical Abuse	2.1* (1.2-3.7)*	6.1(0.013)*	2.0* (1.5-2.8)*	19.7.4(<.001)*	0.4* (0.2-0.9)*	5.7(0.017)*	0.7 (0.3-1.9)	0.4(0.53)
Sexual Abuse	11.7* (3.3-42.3)*	14.7(<.001)*	4.6* (1.2-18.1)*	4.9(0.027)*	---	---	---	---
Parent Died	1.3 (0.7-2.4)	0.6(0.46)	1.8* (1.2-2.7)*	7.6(0.006)*	0.4 (0.1-1.3)	2.5(0.11)	0.5 (0.3-1.1)	3.3(0.07)
Parent Divorced	3.4* (1.8-6.2)*	15.6(<.001)*	2.0* (1.2-3.1)*	8.8(0.003)*	0.7 (0.2-2.5)	0.2(0.63)	2.3 (0.8-6.5)	2.7(0.10)
Other Parent Loss	1.2 (0.3-4.3)	0.1(0.79)	2.1* (1.3-3.4)*	8.9(0.003)*	0.3 (0.0-2.2)	1.6(0.20)	1.5 (0.2-9.2)	0.2(0.66)
Family Violence	0.9 (0.3-2.7)	0.0(0.89)	1.1 (0.5-2.4)	0.1(0.75)	0.5 (0.1-2.8)	0.6(0.42)	1.4 (0.4-5.1)	0.3(0.59)
Physical Illness	1.4 (0.4-5.2)	0.3(0.61)	1.4 (0.6-3.1)	0.7(0.41)	0.6 (0.2-2.7)	0.4(0.54)	1.0 (0.3-3.7)	0.0(0.98)
Financial Adversity	1.5 (0.5-4.5)	0.6(0.45)	1.0 (0.5-2.0)	0.0(0.99)	1.8 (0.5-6.7)	0.8(0.37)	1.1 (0.3-3.8)	0.0(0.84)
group significance test for all types		32.5(<.001)*		44.5(<.001)*		586.1(<.001)*		18.4(0.018)*
significance test for difference between types		16.2(0.023)*		6.9(0.44)		569.3(<.001)*		12.6(0.08)
2+ adversities	0.7 (0.2-1.8)	1.4(0.25)	0.4* (0.2-0.8)*	6.4(0.011)*	3.2 (0.4-23.8)	1.4(0.24)	2.6 (0.7-9.6)	2.2(0.14)
Group significant test of controls: demographic variables		408.3(<.001)*		1173.4(<.001)*		247.8(<.001)*		188.1(<.001)*
Group significance of controls: interactions between demographics and intervals		819.9(<.001)*		1303.7(<.001)*		1230.7(<.001)*		391.3(<.001)*
Group significance of controls: demographics and interactions between demographics and intervals		9644.8(<.001)*		5395.1(<.001)*		3699.2(<.001)*		1530.7(<.001)*
Group significance of controls: parent psychopathology		5.2(0.07)		5.5(0.06)		19.5(0.003)*		
Group significance of controls: mental disorders		121.9(<.001)*		131.1(<.001)*		33.5(<.001)*		29.5(<.001)*

*Significant at the .05 level, two-sided test
LT: lifetime

[1] Assessed in Part 2 sample due to having part 2 controls. Controls for the model include intervals (1-5 intervals), and also include significant variables from demographic and parent psychopathology models, details in the following footnotes.
[b]Models controls for intervals (1-5 intervals), demographics (sex, age, time-varying education), interaction between intervals(13-19,20-29,30+) and age, education. For parent psychopathology, controlling for number of parental disorders (dummies for 1, 2+ disorders).
[c]Models controls for intervals (1-5 intervals), demographics (sex, age, time-varying education), interaction between intervals(13-19,20-29,30+) and age, education. For parent psychopathology, controlling for number of parental disorders (dummies for 1, 2+ disorders).
[d]Models controls for intervals (1-5 intervals), demographics (sex, age, time-varying education), interaction between intervals(13-19,20-29,30+) and age, education. For parent psychopathology, controlling for types of parental disorders (6 dummies).
[e]Models controls for intervals (1-5 intervals), demographics (sex, age, time-varying education), interaction between intervals(13-19,20-29,30+) and age, education. Parent psychopathology not controlled for due to insignificance in previous models.

For peer review only - http://bmjopen.bmj.com/site/about/guidelines.xhtml

Association between childhood adversities and suicidality

For peer review only - http://bmjopen.bmj.com/site/about/guidelines.xhtml

Association between childhood adversities and long-term suicidality among South Africans: Results from the South African Stress and Health Study: A cross sectional study design

For peer review only

For peer review only - http://bmjopen.bmj.com/site/about/guidelines.xhtml

ABSTRACT

Objective:

Suicide and suicidal behaviours are significant public health problems and a leading cause of death worldwide and in South Africa. We examined the association between childhood adversities and suicidal behaviour over the life course.

Methods:

A national probability sample of 4,351 South African adult participants (aged 18 years and older) in the South African Stress and Health (SASH) study was interviewed, as part of the World Mental Health Survey initiative. Respondents provided socio-demographic and diagnostic information, as well as an account of suicide-related thoughts and behaviours. Outcomes were defined as suicide attempts and suicidal ideation in the total sample, and suicide plans and attempts among ideators. Childhood adversities included physical abuse, sexual abuse, parental death, parental divorce, other parental loss, family violence, physical illness and financial adversity. The association between suicidality and childhood adversities was examined using discrete-time survival models.

Results:

More than a third of respondents with suicidal behaviour experienced at least 1 childhood adversity, with physical abuse, parental death and parental divorce the most prevalent adversities. Physical abuse, sexual abuse and parental divorce were identified as significant risk markers for lifetime suicide attempts, while physical abuse and parental divorce were significantly correlated with suicidal ideation. Two or more childhood adversities were associated with a 2-fold higher risk of lifetime suicide attempts. Sexual abuse (OR=9.3, childhood, parental divorce (OR=3.1)

For peer review only - http://bmjopen.bmj.com/site/about/guidelines.xhtml

and physical abuse (OR=2.2) had the strongest associations with lifetime suicide attempts. The effect of childhood adversities on suicidal tendencies varied over the *life course.* For example, sexual abuse was significantly associated with suicide attempts during childhood and teen years, but not during young and later adulthood.

Conclusions:

Childhood adversities, especially sexual abuse, physical abuse and parental divorce are important risk factors for the onset and persistence of suicidal behaviour, with this risk greatest in childhood and adolescence. The risk for suicidal behaviour was greatest in childhood and adolescence. Suicidal risk in childhood and adolescence was significantly associated with the following childhood adversities: sexual abuse, physical abuse and parental divorce.

Keywords: Childhood adversities, suicidal ideation, suicidal attempts

For peer review only

For peer review only - http://bmjopen.bmj.com/site/about/guidelines.xhtml

INTRODUCTION

Suicide and suicidal behaviour are significant public health problems. Suicide is one of the leading causes of death worldwide with almost 1 million people committing suicide each year [1]. This figure is likely to grow to approximately 1.2 million suicides in 2020 [2]. In South Africa, the annual rate of suicide is high [3, 4] mirroring international trends [5]. So, too, are rates of suicidal behaviour with an estimated prevalence of 9.1% for lifetime suicidal ideation and 2.9% for suicide attempts among South Africans according to the South African Stress and Health Survey (SASH) [6].

Despite the enormity of the problem, the aetiology of suicidal behaviour is not fully understood. There are controversies in the literature regarding prior psychiatric disorder and risk for suicide attempts. While some authors have argued that pre-existing disorder is an important risk factor (7-11], others have argued that suicide attempts are not neccessarily associated with prior psychopathology [12]. Genetic factors also play an important role in suicidal behaviour [13-16]. While there is stronger evidence pointing towards environmental or experiential factors [17, 18] such as exposure to childhood adversities (19-28]. Recent multi-level country data from the World Mental Health Surveys (WMHS) initiative has allowed for cross-national comparisons of suicidality. The WMHS investigated the association between childhood adversities and suicidal behaviour [20], the persistence of suicidality over time, and the extent to which associations between childhood trauma and suicidality changed over the life course. The WMHS found a dose-response relationship between the number of adversities and suicidal behaviour. Sexual abuse and physical abuse were the strongest risk factors for both the onset and persistence of suicidal behaviours, with the risk for suicidality greatest during childhood (age 4-12 years) and adolescence (age 13-19 years) [20].

For peer review only - http://bmjopen.bmj.com/site/about/guidelines.xhtml

Numerous studies have examined the link between childhood sexual abuse and suicidality [29-41]. All of these authors have found that exposure to childhood sexual abuse increases the risk for mental disorders, including suicidality. Furthermore, the majority of studies that have focused on the link between childhood physical abuse and suicidality have found that exposure to childhood physical abuse increases the risk for suicidality [42, 43]. There also appears to be an association between the number of childhood adversities experienced and the later suicidal behaviour [21, 23, 24, 44, 45].

Exposure to early life stress is prevalent among South Africans. In one sample of South African rural youth, the prevalence of physical and sexual abuse was shown to be very high with 94.4% of males exposed to physical abuse and 39.1% of females to sexual abuse [46]. More than a quarter of adults who were interviewed endorsed exposure to childhood adversity (parental death, parental separation or parental divorce) in the SASH study [47]. Significantly more females were prone to be victims of domestic violence than men [47]. Women also reported twice as many suicidal attempts than the male participants in the SASH study [9].

Objective

We report in more detail on data from a South African dataset gathered as part of the World Mental Health Surveys, which allowed for comparison with data from the overall cross-national sample. This data are particularly interesting as South Africa is a middle income African country with high rates of violent trauma exposure. The present study aimed to examine the relationship between the type and frequency of childhood adversity exposure to suicidal behaviour over the life trajectory of South Africans, given that there are no published nationally representative data that may be useful in informing both clinical practice and policy.

For peer review only - http://bmjopen.bmj.com/site/about/guidelines.xhtml

METHODS

Sample

Data for the SASH Study were collected between January 2002 and June 2004. WMH surveys were carried out in 21 countries which included Nigeria and South Africa [48]. For detailed information on study methods see Williams et al. (2004) [48]. The research protocol for the SASH study was approved by the Human Subjects Committee of the University of Michigan, by Harvard Medical School ethics committee and by a single project assurance of compliance from the Medical University of South Africa (MEDUNSA), and by the National Institute of Mental Health. It was a national probability sample of 4,351 South African adults (persons aged 18 years and older) living in households or in hostel accommodation. All racial and ethnic groups were represented, with the sample selected using a three-stage probability sample design. The response rate was 85.5%.

Sampling approach

Sampling was divided into three stages. Primary sampling units was selected during the first stage, which was based on the 2001 SA census Enumeration Areas (EAs). The second stage involved sampling of household units within clusters selected in each EA. South Africans in both urban and rural areas were sampled. Sampled residences were stratified into 10 diverse housing categories: Rural–commercial, agricultural, rural traditional subsistence areas, African townships, informal urban or peri-urban shack areas, Coloured townships, Indian townships, general metropolitan residential areas, general large metropolitan residential areas, and domestic servant accommodation in urban areas. During the third stage, one adult respondent in each sampled housing unit was selected. A total of 5089 households was selected. Field interviews were conducted with 4433 (87.1%) of designated respondents. Based on quality control, 4351

For peer review only - http://bmjopen.bmj.com/site/about/guidelines.xhtml

interviews were retained for use in the analysis. There were no differences in response rates across the four designated racial groups (white, Coloured [mixed racial origin], Indian, black). According to the 2001 Census statistics, 79.% people in South Africa are Black African, 8.9% are coloured, 9.6% are white, and 2.5% are Indian/Asian [49].

Diagnostic Interview

SASH used version 3 of the World Health Organization Composite Diagnostic Interview (WHO CIDI) [50]. Interviewers were trained within a one week period and conducted the interviews in seven different languages, namely English, Afrikaans, Zulu, Xhosa, Northern Sotho, Southern Sotho, and Tswana. Translations of the CIDI into several native South African languages were conducted in accordance with WHO requirements. Multilingual and bilingual expert panels conducted the back-translations [51, 52]. Informed consent was obtained from participants after a complete description of the study was provided. Respondents provided socio-demographic and diagnostic information, as well as an account of suicidal behaviours during the interviews. The core diagnostic assessment of mental disorders included anxiety disorders (panic disorder, agoraphobia, social phobia, generalized anxiety disorder, post-traumatic stress disorder), mood disorders (major depressive disorder, dysthymia), substance use disorders (alcohol abuse, alcohol dependence, drug abuse, drug dependence) and intermittent explosive disorder [53, 54].

Suicidal behaviour

The CIDI 3.0 module on suicidal behaviour was used to assess the age-of-first-onset, age of most recent episode, and lifetime occurrence of suicidal ideation, suicide plans and suicide attempts. Suicidal ideation, suicide plans and suicide attempts was assessed with questions such as “Have you ever seriously thought about committing suicide?”, “Have you ever made a plan for committing suicide?”, and “Have you ever attempted suicide?”, respectively. Ideators only

For peer review only - http://bmjopen.bmj.com/site/about/guidelines.xhtml

proceeded to answer questions about plans ("Have you ever made a plan for committing suicide?") and attempts ("Have you ever attempted suicide?"). Information on the age of first occurrence of the three main outcomes was obtained. To get a better understanding of the progression from ideation to attempt, the outcomes considered in this study were: suicide attempts in the total sample; suicide ideation in the total sample; suicide plans among ideators; suicide attempts among ideators with a plan (planned attempts), and suicide attempts among ideators in the absence of a plan (unplanned or impulsive attempts).

Childhood adversities

Physical abuse, sexual abuse, parental death, parental divorce, other parental loss, family violence, physical illness and financial adversity were the various childhood adversities assessed. Biological and non-biological parents were included in measures of parental death, divorce or other parental loss. Financial adversities were assessed with questions on whether the family had insufficient funds to pay for basic necessities. Questions about repeated fondling, attempted rape or rape were asked to assess for sexual abuse. This comprised the following "The next 2 questions are about sexual assault: (i) The first is about rape. We define this as someone either having sexual intercourse with you or penetrating your body with a finger or object when you did not want them to, either by threatening you or using force, or when you were so young that you didn't know what was happening. Did this ever happen to you?", and (ii)"Other than rape, were you ever sexually assaulted or molested?". A modified version of the Conflict Tactics Scale (CTS2) was used to assess family violence and physical abuse [55]. Respondents were classified as having experienced *physical abuse* when they indicated that, when they were growing up, their father or mother (includes biological, step, or adoptive parents) slapped, hit, pushed, grabbed, shoved, or threw something at them, or that they were beaten as a child by the persons

For peer review only - http://bmjopen.bmj.com/site/about/guidelines.xhtml

who raised them. Family violence was assessed as present when respondents indicated that they (i) "were often hit, shoved, pushed, grabbed, or slapped while growing up" *or* (ii) "witnessed physical fights at home, like when your father beat up your mother?" A standard chronic conditions checklist assessed for life-threatening physical illnesses in childhood [56].

Data analysis

All data analyses were processed and analysed centrally by a team of statisticians at the Harvard School of Public Health (Boston, USA) using the SAS version 9.1.3 software package. Discrete-time survival analysis with time-varying covariates was used to study the risk factors of lifetime suicide ideation, plans and attempts. Data were weighted to adjust for the stratified multistage sample design, differential probability of selection within households as a function of household size and clustering of data, and differential non-response. Overall, percentages were weighted to adjust for differences in selection probabilities, differential non-response, oversampling of cases, and residual differences on sociodemographic variables between the sample and the population [48, 57]. A post-stratification weight was also used to make the sample distribution comparable, for age, sex, and province, with the population distribution in the 2001 South African census. Both weighted and geographic clustering of data were taken into account in the data analyses by using a jackknife repeated replications simulation method implemented in SAS macro 14. The survival coefficients were exponentiated and are reported below in the form of odds ratios.

The association between suicidality and childhood adversity was examined using discrete-time survival models with the analysis unit being person-years. Bivariate analyses (considering one adversity at a time) and multivariate analyses (considering all adversities simultaneously) were conducted. Two types of multivariate models were tested: multivariate additive models (simultaneously considering all childhood adversities) and multivariate interactive models (with

For peer review only - http://bmjopen.bmj.com/site/about/guidelines.xhtml

number and type of childhood adversities experienced by each respondent included as dummy variables). The analysis also examined interactions between the life stage (13-19 years, 20-29 years, 30+ years) of respondents and each childhood adversity, as well as the influence each adversity had on early-, middle- and later- onset suicidality. Analyses were conducted using SUDAAN version 8.1 to adjust for clustering and weighting. Odds ratios (ORs) with a 95% confidence interval (CIs) are reported. Wald X^2- tests were used to examine multivariate significance. Associations between adversities and suicide outcomes were adjusted for sex-, age, educational level, marital status, interactions between demographic variables, life course, lifetime mental disorders and parental psychopathology. Analyses also examined the influence of respondents' lifetime mental disorders on suicidality, as well as interactions between sex and each childhood adversity. Statistical significance using two-sided tests was set at p <.05 [20]. Based on an N of 4000 (alpha of 0.05, 2 sided significance), the study was adequately powered (.99), to detect an OR of 2.0 of a continuously distributed normalized predictor and a 10% prevalence of suicidal behaviour.

RESULTS

Demographic details

In the sample, (n = 4351), there were slightly more female (53.7%) than male respondents. There were more black (76.2%) than coloured (10.4%), white (10%), and Indian/Asian (3.4%) respondents. Furthermore, half of the sample was married and most were unemployed (69.2%), had less than 12 years of education (62.7%) and lived in an urban area (59.7%) (see Table 1).

Prevalence of childhood adversities among the total sample

Figure 1 provides a schematic representation of the suicidality data reported in the sections which follow. In the total sample, 35.4% of participants with one adversity had a suicide attempt,

For peer review only - http://bmjopen.bmj.com/site/about/guidelines.xhtml

compared with 23.4% with one adversity who had not made an attempt. Physical abuse (24.9%), parental divorce (14.2%) and parental death (11.6%) were most prevalent among those suicide attempters. Among those exposed to one childhood adversity, without a suicide attempt, the two most prevalent adversities reported were physical abuse (12.2%) and parental death (11.3%). In the total sample 15.4% of participants exposed to two or more adversities had a suicide attempt. In contrast, 8.6% of participants exposed to two or more adversities had not made an attempt (Table 2).

Prevalence of childhood adversities among suicidal ideators in the total sample

In the sample as a whole, 35.9% of those with one adversity had suicidal ideation compared with 22 .7% of those with one adversity who had no ideation. The most prevalent adversities associated with suicidal ideation were physical abuse (21.1%), parental death (13.9%), and parental divorce (7.9%). Among those without suicidal ideation, physical abuse (11.8%) and parental death (11.3%) were the most commonly endorsed childhood adversities. Of those who endorsed two or more childhood adversities, 10.8% reported suicidal ideation and 8.6% did not (Table 2). In summary, the most prevalent childhood adversities reported among the total sample with/without suicidal ideation were firstly, physical abuse and secondly, the death of a parent.

Prevalence of suicide attempts in the total sample

In the total sample, 24.9% of those with childhood physical abuse had attempted suicide while 12.2% of respondents with no physical abuse had no attempt. Of those exposed to parental divorce, 14.2% had attempted suicide and 4.8% had made no attempt. The second most prevalent childhood adversity was parental death with 11.6% of those with parental death attempting suicide and 11.3% of those with parental death with no attempts (Table 2).

For peer review only - http://bmjopen.bmj.com/site/about/guidelines.xhtml

Prevalence of childhood adversities among suicidal ideators

With/without a plan

Among suicidal ideators with a plan, 32.9% had experienced one childhood adversity. Among ideators with no plan, 41.7% had one childhood adversity. Among ideators with a plan, the following were the most prevalent childhood adversities: physical abuse (24.3%), parental death (12.2%), and parental divorce (9.7%). Among ideators without a plan, 27.9% endorsed physical abuse, 16.1% parental death, and 9.2% parental divorce (see Table 2). In both groups (ideators with and without a plan), physical abuse was the most prevalent childhood adversity, followed by parental death and parental divorce.

With or without an attempt

Among suicidal ideators who had attempted suicide, 35.4% were exposed to one childhood adversity and 15.4% were exposed to two or more childhood adversities. In the group of ideators who had made an attempt, 24.9% had experienced physical abuse, 14.2% parental divorce, and 11.6% parental death (Table 2). 40.5% of those with one adversity, and 9.6% of those exposed to two or more adversities were suicidal ideators with no attempts. In this group, the most prevalent adversities were physical abuse (24.5%), parental death (15.6%) and parental divorce (6.7%) reported (Table 2).

Among all ideators (with/without a plan, with/without an attempt), the most prevalent childhood adversity was physical abuse, followed by parental death and parental divorce. Of note, in the group of ideators with an attempted suicide parental divorce was more prevalent than parental death.

Bivariate and multivariate results: Type of childhood adversity

For peer review only - http://bmjopen.bmj.com/site/about/guidelines.xhtml

Bivariate and multivariate analyses were performed to examine the associations between the different childhood adversities (physical abuse, sexual abuse, parental death, parental divorce, other parental loss, family violence, physical illness, financial adversity) and lifetime suicidal ideation, plans and attempts.

In the total sample, bivariate and multivariate analysis revealed significant associations between (i) sexual abuse (bivariate: OR=7.9, p=0.003; multivariate: OR=7.6, p=0.003), (ii) physical abuse (OR 2, p=0.007; OR 2.0, p=0.006) and (iii) parental divorce (OR 2.8, p<.001; OR 2.7, p=0.001), and lifetime suicide attempts. Among ideators in the sample, physical abuse (OR=1.7, p<.001; OR=1.7, p<.001) was significantly associated with suicidal ideation. Multivariate analyses revealed an additional association with suicidal ideation, namely parental divorce (OR = 1.6, p=0.038). The relationship between childhood adversities and lifetime plans was not statistically significant. However, a significant association was found between parental divorce and lifetime suicidal attempts among ideators (OR=3.0, p<.001; OR=3.1, p=0.023) (Table 3).

Findings from multivariate analysis, therefore, confirm findings of bivariate analysis for all groups, except for ideators. Among ideators bivariate analysis revealed a significant relationship between physical abuse and suicidal ideation. This was confirmed in multivariate analysis where the association between parental divorce and suicidal ideation was significant for the whole sample.

Bivariate associations between the number of adversities and lifetime suicidality

The relationship between the number of childhood adversities and lifetime suicidal ideation, plans and attempts was further examined. There was a significant relationship between the number of childhood adversities and lifetime suicide attempts. Two or more childhood

For peer review only - http://bmjopen.bmj.com/site/about/guidelines.xhtml

adversities were associated with a 2-fold higher risk of lifetime suicide attempts in the total sample (OR=2.1, p<.001). A significant relationship was also established between one, as well as two or more adversities with ideators in the total sample. Among ideators, no significant association was found between the number of childhood adversities and lifetime plans. A significant relationship was found between two or more adversities and lifetime attempts among ideators (OR=2.7, p=0.016), indicating a more than 2-fold higher risk of lifetime suicide attempts in this group (Table 4).

Multivariate associations between number of childhood adversities and lifetime suicidality

In the final multivariate model which included 2 or more adversities as a predictor variable, sexual abuse (OR=9.3, p<.001), childhood physical abuse (OR=2.2, p=0.003) and parental divorce (OR=3.1, p<.001) retained significant associations with lifetime suicide attempts in the total sample. Physical abuse (OR=2.1, p<.001), parental death (OR=1.7, p=0.010), parental divorce (OR=1.9, p=0.004) and other parental loss (OR = 2.1, p=0.004) were significant predictors of suicidal ideation (Table 5a). The same findings emerged after controlling for mental disorders, with the exception that sexual abuse was also significantly associated with suicidal ideation (Table 5b). Physical abuse was associated with a lower odds of lifetime suicide plans among ideators (OR = 0.4, p=0.038) (Table 5a). There were no significant associations between childhood adversities and lifetime attempts among those with suicidal ideation. (Table 5). The findings remain unchanged after controlling for mental disorders (Table 5b).

Associations between the types of childhood adversity and lifetime suicidality over the life course

Multivariate analyses were performed to examine the association between the types of childhood adversity and lifetime suicidal ideation, plans and attempts during childhood years (age 4- 12),

For peer review only - http://bmjopen.bmj.com/site/about/guidelines.xhtml

teenage years (age 13-19), young adulthood (age 20-29) and later adulthood (30 years and older) (Tables available from authors).

Childhood years (4-12). Sexual abuse (OR=61.6, CI=4.5-841.0, p=0.002) in early childhood (4-12 years of age) was significantly associated with lifetime suicide attempts in the total sample (OR = 61.6, CI=4.5-841.0, p=0.002). Both sexual abuse (OR=34.8, CI= 3.1-392.6, p=0.003) and physical abuse (OR=3.7, CI=1.0-13.4, p=0.041) were associated with a higher risk for suicidal ideation among the total sample. No significant associations were found between any of the childhood adversities and lifetime plans in the group of ideators. Among those with suicidal ideation, parental death (OR=2.2, CI=1.1-4.3, p=0.021) was significantly associated with suicide attempts in childhood years.

Teen years (13-19). Sexual abuse (OR=20.3, CI=2.0-210.2, p=0.010), physical abuse (OR=3.7, CI=1.5-9.2, p=0.004), and parental divorce (OR=4.6, CI=1.7-12.1, p=0.002) were significantly associated with suicide attempts in the total sample of teenagers. Physical abuse (OR=3.6, CI=2.2-5.9, p<.001) and parental death (OR=2.2, CI=1.1-4.3, p=0.021) significantly increased the risk for suicidal ideation among the total group of teens. Physical illness (OR=9.9, CI=1.8-54.0, p=0.007) significantly increased the risk of suicidal plans in teens with suicidal ideation. Suicide attempts among teens with suicidal ideation was significantly predicted by parental divorce (OR=4.3, CI=1.1-17.0, p=0.035).

Young adulthood (20-29). None of the childhood adversities were significantly associated with lifetime suicide attempts during young adulthood in the sample overall. An explanation could be that suicide attempts spike earlier and later in life among South Africans, contributing to the lack

For peer review only - http://bmjopen.bmj.com/site/about/guidelines.xhtml

of significance. Parental loss other than parental death was significantly associated with suicidal ideation (OR=2.9, CI=1.2-7.4, p=0.019).

Later adulthood (≥ 30). Childhood physical abuse (OR=2.2, CI=1.0-4.8, p=0.035) was significantly predictive of suicidal attempts. The likelihood of suicidal ideation significantly increased in later adulthood if parental loss other than parental death (OR=5.1, CI=2.1-12.1, p<.001) or physical illness had been present during childhood (OR=4.3, CI=1.1-15.9, p=0.028). No significant relationship was found between any of the childhood adversities and lifetime plans in the group of ideators although a significant relationship was found between two or more adversities and lifetime plans among those who were ideators (OR=44.5, CI=2.5-779.1, p<0.008). None of the childhood adversities were significantly associated with suicide attempts among ideators in this age group.

DISCUSSION

Rates of childhood adversities and suicidal behaviours were both high among South Africans, with more than a third of respondents in the total sample who attempted suicide experiencing one childhood adversity, and 15.4% experiencing two or more adversities. Overall, physical abuse, sexual abuse, parental divorce and physical illness were far more prevalent in those with a suicide attempt than in those without. The most prevalent childhood adversities endorsed overall were physical abuse followed by parental death. Physical abuse, parental divorce and death of a parent were also the most prevalent adversities experienced in those with a suicide attempt as well as in those with suicidal ideation. These findings are somewhat dissimilar to other country samples; for example in the 21 countries that participated in the WMHS, physical abuse (29.3%), family violence (24.8%) and neglect (19.3%) were the most prevalent childhood adversities among those with a lifetime suicide attempt, while physical abuse (20.6%), family violence (17.6%) and death

For peer review only - http://bmjopen.bmj.com/site/about/guidelines.xhtml

of a parent (14.2%) were most often reported among participants with lifetime suicidal ideation [20]. Cross-nationally, it would appear that physical abuse is the commonest childhood adversity associated with lifetime suicide attempts and ideation [20].

The estimate lifetime prevalence of 2.9% for attempted suicide among South Africans is close to the rates of 4.6% and 4.1% reported for general and Black populations respectively in USA. In addition the 9.1% estimated prevalence of suicide ideation is comparable with previous estimates from studies in South African clinical samples. Joe et al. (2008b) reported for the first time on the rates of suicide ideation, plan and attempts among the different ethnic groups, in data from the SASH study [6]. Overall, the results suggest that people in SA engage in suicidal thought and behaviours at levels nearly comparable with those of Western nations.

When examining suicidal behaviour risk in the context of childhood adversity, sexual abuse, physical abuse and parental divorce emerged as significant risk factors for lifetime suicide attempts in the total sample. Furthermore, physical abuse and parental divorce were significant risk factors for suicidal ideation in the total sample,. After adjusting for mental illness, sexual abuse was also a significant risk factor for suicidal ideation. Parental divorce emerged as a significant risk factor among ideators with lifetime suicide. These findings are largely consistent with the data from the overall cross-national WMHS, which found that physical and sexual abuse significantly increased the likelihood of suicidal ideation and attempts, while neglect was a risk factor for suicidal behaviour in multivariate additive analyses [20].

Of the adversities implicated, sexual and physical abuse were more significant risk factors than other adversities, highlighting the fact that intrusive and aggressive experiences in childhood may have more devastating and longer lasting effects [58]. This may be due to the extreme

For peer review only - http://bmjopen.bmj.com/site/about/guidelines.xhtml

powerlessness and loss of control that such abuse causes, or to physically aggressive assaults resulting in the devaluation of one's body and consequent susceptibility to self harm [28]. In a country with high rates of sexual and physical abuse [46] this is particularly concerning. The impact of parental divorce on suicidality supports previous findings that parental divorce, if accompanied by other adversities such as childhood abuse, increases the risk of suicidal behaviour [59].

We also found that exposure to *two or more childhood adversities* significantly increased the risk of suicide attempts among ideators. This confirms earlier work showing exposure to multiple childhood adversities increases the risk of suicidal behaviour [21, 23, 24, 60, 61]. Bruffaerts et al (2010) found a sub-additive effect with regards to the onset of suicidal behaviour when considering multiple adversities [20]. Thus, the impact of multiple adversities was not equal to the sum of the odds ratios of individual adversities. In the overall WMHS analysis exposure to multiple childhood adversities had a significant effect on the persistence of suicide when considering every additional childhood adversity exposed to, however in the current study it was not possible to stratify the number of adversities beyond two or more adversities (i.e. into more than 2 categories) given the relatively small number of cases in the sample overall with non-fatal suicidal behaviour. Physical abuse, parental death, parental loss other than through death, and parental divorce emerged as independent risk factors for suicidal ideation in the total sample. Moreover, the effects of childhood adversities on suicidal tendencies tended to differ over the *life course*. Consistent with nationally representative data in WMHS, childhood adversities were associated with the highest risk of suicide attempts in childhood, with a decrease in risk in adolescence and young adulthood, followed by an increase in risk again during later adulthood [20].

For peer review only - http://bmjopen.bmj.com/site/about/guidelines.xhtml

In *childhood*, sexual abuse was significantly associated with lifetime suicide attempts in the total sample, while sexual and physical abuse were significantly associated with suicidal ideation. Among suicidal ideators, parental death was significantly associated with lifetime suicide attempts. Exposure to childhood sexual abuse, physical abuse or parental divorce significantly increased suicide attempts during *teenage years*, while physical abuse and parental death were associated with suicidal ideation in teens. Among teen suicidal ideators, physical illness was significantly associated with suicidal plans, while parental divorce was associated with suicide attempts. These findings emphasize the need to focus suicide prevention strategies at youth in particular. In *young adulthood,* parental loss other than the death of a parent was significantly associated with suicidal ideation in the total sample. Interestingly, childhood physical abuse was identified as a significant risk factor for suicidal attempts in *later adulthood*, while childhood physical illness and parental loss other than the death of a parent significantly increased the risk for ideation.

Similar to findings from SASH, childhood sexual abuse emerged as a particularly robust risk factor for suicide attempts in younger participants in the WMH cross-national analysis, with a 10.9 times higher odds of suicide attempts in children, a 6.1 times higher likelihood in adolescents and a 2.9-fold risk in young adults who were exposed [20]. This is in keeping with Enns hypothesis that sexual abuse results in suicidal behaviour at a younger age [21]. Consistent with other studies, childhood physical and sexual abuse, in particular, emerged as risk factors for the emergence and persistence of suicidal behaviour, especially in adolescence. Loss of a parent, physical ill-health and family violence has also been found to be associated with persistence of suicidality [20, 28, 58]. These findings extend previous work done in other developing countries that have found childhood adversities to be a significant risk factor for suicidality [20, 62-64].

For peer review only - http://bmjopen.bmj.com/site/about/guidelines.xhtml

Limitations

The following limitations need to be highlighted. First, recall bias might have impacted on the accuracy of recall of childhood adversities. This said, participants were asked questions about childhood adversities in sequence which may have facilitated more accurate recall [65]. Systematic reviews have also found that recall of past experiences can be accurate and can provide valuable data [66, 67]. Thus, there is evidence to support the validity of accurate recall of childhood adversities [67]. Further, studies have shown that responses to questions on childhood adversities, similar to those asked in the SASH study, generally remain stable over time [68, 69]. We recommend that future studies examine ethnicity in relation to adversity and suicidal outcomes. Second, in view of the cross-sectional design, more detailed, temporal information on childhood adversities and suicidal incidents was not obtained. Third, variables such as culture, ethnicity and mental status at the time of the interview may have influenced the recall and reporting of suicidal behaviour. It is possible that response bias may have been particularly skewed to disenfranchised South Africans (e.g. poor, young, urban an black respondents), who may have been too afraid to divulge information on suicidality. Stigma associated with mental health problems may have also played a role in the reporting suicidal tendencies. Thus, participants' mental health status, ethnicity, culture and generational factors may have also contributed to the under-reporting of suicidality. It is possible that individuals reporting childhood adversities may have also been more likely to report suicidal behaviour, while those not reporting childhood adversities may have underreported suicidality. Stigma and mental health status (e.g. depressed persons may be more inclined to report suicidality and more likely to remember negative childhood experiences) may also be contributory factors. In addition, some participants

For peer review only - http://bmjopen.bmj.com/site/about/guidelines.xhtml

may have been afraid to report suicidal behaviours. The role of ethnicity, culture and generational factors may have also contributed to the under-reporting of suicidality. Overall, it is much more likely that adversities and suicidality were under-reported rather than over-reported [9, 20, 67, 70]. Fourth, we do not assess for self-mutilating behavior. The importance of discriminating suicidal behaviour from non-suicidal self-mutilation cannot be underestimated. Fifth, the survey was conducted in adults living in households and hostel quarters thus the findings are not generalizable to homeless and institutionalized persons who were not included in the survey. Sixth, the CIDI instrument which was used in this study is a lay-administered instrument which does not include an assessment of several key DSM-IV diagnoses (such as bipolar disorder and psychosis), are associated with elevated rates of suicidality. As a result, some participants with suicidality may have not have been diagnosed with a disorder. Furthermore, in view of the large confidence intervals and small sample sizes for some of these analyses caution is required in drawing conclusions. In addition, we did not control for other unmeasured causes of childhood adversities and suicidality, or protective (resiliency) factors that may have contributed to the associations observed in these data. Both other risk and resiliency factors may have contributed to both the prevalence of non-fatal suicidal behaviours and to the associations with different forms of childhood adversity and warrant further investigation. Lastly, it is important to point out that these data were collected approximately 10 years ago. Notwithstanding these limitations, this study represents the first investigation among South Africans of a wide range of childhood adversities and their impact on the onset and persistence of suicidality over the life course.

Conclusions

Childhood adversities especially sexual abuse, physical abuse and parental divorce are associated with the onset and persistence of suicidal behaviour with the risk greatest in children and

For peer review only - http://bmjopen.bmj.com/site/about/guidelines.xhtml

adolescents. Public health efforts aimed at prevention of early childhood sexual and physical abuse, in particular, may have a significant impact on reducing suicidality over the life course and improving mental health outcomes.

For peer review only

For peer review only - http://bmjopen.bmj.com/site/about/guidelines.xhtml

REFERENCES

1. World Health Organization. Suicide Prevention (SUPRE). Geneva, Switzerland. 2007. http://www.who.int/mental_helath/prevention/suicide/suicideprevention/en/)

2. Murray, C.L., Lopez, A.D. The global burden of disease: a comprehensive assessment of mortality and disability from disease, injuries and risk factors in 1990 and projected to 2020. Cambridge, MA: Harvard University Press, 1996.

3. Burrows, S., Laflamme, L. Pattern analysis of suicide mortality surveillance data in urban South Africa. Suicide and Life- Threatening Behaviour 2008;**38**:209-220.

4. Meel, B.I. Epidemiology of suicide by hanging in Transkei. South Africa. Am J Forensic Med Pathol. 2006;**27**:75-78

5. Flisher, A.J., Liang, H., Laubscher, R. Suicide trends in South Africa, 1968-90. Scand J Public Health 2004;**32**:411-418.

6. Joe, S., Stein, DJ., Seedat, S., Herman, A., Williams, DR. non-fatal suicidal behavior among South Africans: Results from the South Africa Stress and Health Study. Social Psychiatry Epidemiology 2008;**43**(6):454–461.doi:10.1007/s00127-008-0348-7.

7. Beautrais, A.L., Joyce, P/R/. & Mulder, R.T. (1996). Risk factors for serious suicide attempts among youth aged 13 through 24 years. J Am Acad Child Adolesc Psychiatry 1996;**35**(9):1174-1182.

8. Harrison, EC, Barraclough, B. (1997). Suicide as an outcome for mental disorders: A meta-analysis. Br J Psychiatry 1997;**170**:205-228

For peer review only - http://bmjopen.bmj.com/site/about/guidelines.xhtml

9. Joe, S., Stein, D.J., Seedat, S., et al. Prevalence and correlates of non-fatal suicidal behaviour among South Africans. Br J Psychiatry 2008;**192**:310-311.

10. Nock, M.K., Borges, G., Bromet, E.J., et al. Suicide and Suicidal Behaviour. Epidemiologic Reviews 2008;**30**:133-154.

11. Nock, M.K., Borges, G., Bromet, E.J., et al. (2008b). Cross-national prevalence and risk factors for suicidal ideation, plans and attempts. *British Journal of Psychiatry*, 192, 98-105.

12. Nock, M.K., Hwang, I., Sampson, N.A., et al. Cross-national analysis of the associations among mental disorders and suicidal behaviour: Findings from the WHO World Mental Health Surveys. PLos Medicine 2009;**6**(8).e1000123.

13. Bondy, B., Buettner, A., Zill, P. Genetics of suicide. Molecular Psychiatry 2006;**11**:336-351.

14. Kohli, M.A., Salyakina, D., Pfennig, A., et al. Association of genetic variants in the neurotrophic receptor encoding gene NTRK2 and a lifetime history of suicide attempts in depressed patients. Arch Gen Psychiatry 2010;**67**:348-59.

15. Roy, A., Hu, X-Z., Janal, M.N., & Goldman, D. Interaction between childhood trauma and serotonin transporter gene variation and suicide. Neuropsychopharmacology 2007;**32**:2046–2052

16. Risch, N., Herrell, R., Lehner, T., et al. Interaction between the serotonin transporter gene (5-HTTLPR), stressful life events, and the risk of depression: A meta-analysis. JAMA 2009;**301**:2462–2471.

17. Borges, G., Benjet, C., Medina-Mora, M.E., et al. Traumatic events and suicide related outcomes among Mexico City adolescents. J Child Psychol Psychiatry 2008;**6**:654-666.Weissman MM, Bland

For peer review only - http://bmjopen.bmj.com/site/about/guidelines.xhtml

RC, Canino GJ, Greenwald S, Hwu HG, Joyce PR, et al. (1999) Prevalence of suicide ideation and suicide attempts in nine countries. *Psychology Med, 29*: 9–17.

18. Brodsky, BS & Stanley, B. Adverse childhood experiences and suicidal behaviour. Psychiatry Clinical Northern America 2008;**31**:223-235

19. Bruffaerts, R., Demyttenaere, K., Borges, G., et al. Childhood adversities as risk factors for onset and persistence of suicidal beahviour. Br J Psychiatry 2010;**197**:20-27.

20. Enns, M.W., Cox, B.J., Afifi, T.O., et al. Childhood adversities and risk for suicidal ideation and attempts: a longitudinal population-based study. Psychological Medicine 2006;**36**:1769-1778.

21. Johnson, J.G., Cohen, P., Gould, M.S., et al. Childhood adversities, interpersonal difficulties, and risk for suicide attempts during late adolescence and early adulthood. Arch Gen Psychiatry 2002;**59**:741-749.

22. Dube, S.R., Anda, R.F., Felitti, V.J., et al. Childhood abuse, household dysfunction, and the risk of attempted suicide throughout the life span: Findings from the Adverse Childhood Experiences Study. JAMA 2001;**286**:3089-3096.

23. Afifi, T.O., Enns, M.W., Cox, B.J., et al. Population attributable fractions of psychiatric disorders and suicide ideation and attempts associated with adverse childhood experiences. Am J Public Health 2008;**98**:946-952.

24. Burke, A.K., Galfalvy, H., Everett, B., et al. Effect of exposure to suicidal behavior on suicide attempt in a high-risk sample of offspring of depressed parents. J Am Acad Child Adolesc Psychiatry 2010;**49**:114-121.

For peer review only

For peer review only - http://bmjopen.bmj.com/site/about/guidelines.xhtml

25. Labonte, B., Suderman, M., Maussion, G., Navaro, L., Yerko, V., Mahar, I., & Turecki, G. Genome-wide epigenetic regulation by early-life trauma. Arch Gen Psychiatry 2012;**69**(7):722-731.Doi:10.1001/archgenpsychiatry.2011.2287

26. Lipschitz, D.S., Winegar, R.K., Nicolaou, A.L., et al. (1999). Perceived abuse and neglect as risk factors for suicidal behaviour in adolescent inpatients. *The Journal of Nervous and Mental Disease*, 187, 32-39.

27. Ystgaard, M., Hestetun, I., Loeb, M., & Mehlum, L. Is there a specific relationship between childhood sexual and physical abuse and repeated suicidal behaviour? Child Abuse Neg 2004;**28**:863-875

28. Boudewyn, A., & Liem, J. Childhood sexual abuse as a presecutor to depression and self-destructive behavior in adulthood. J Trauma Stress 1995;**8**:445-459.

29. Brown, J., Cohen, P., Johnson, J.G., & Smailes, E.M. Childhood abuse and neglect: Specificity of effects on adolescent and young adult depression and suicidality. J Am Acad Child Adolesc Psychiatry 1999;**38**:1490-1496.

30. Bryant, S.L., & Range, L.M. Suicidality in college women who were sexually and physically abused and physically punished by parents. Violence Vict 1995;**10**:195-201.

31. Davidson, J.R.T., Hughes, D.C., George, L.K., & Blazer, D.G. The association of sexual assault and attempted suicide within the community. Arch Gen Psychiatry 1996;**53**:550-555

32. Fergusson, D.M., & Mullen, P.E. Childhood Sexual abuse – An evidence based perspective. Sage, CA: Thousand Oaks, 1999.

For peer review only - http://bmjopen.bmj.com/site/about/guidelines.xhtml

33. Finkelhor, D. Early and long-term effects of child sexual abuse: An update. Professional Psychology: Research & Practice 1990;**21**(5):325-330.

34. Finkelhor, D., & Hashima, P.Y. (2001). The victimization of children and youth: A comprehensive overview. In S.O. White (Ed.) Handbook of youth and justice. The Plenum series in crime and justice. Dordrecht: Plenum, 2001:49-78.

35. Holmes, W.C., & Slap, G.B. Sexual abuse of boys: Definition, prevalence, correlates, sequelae, and management. JAMA: JAMA 1998;**280**(21):1855-1862

36. Kendall-Tackett, K.A., Williams, L.M., & Finkelhor, D. Impact of sexual abuse on children: A review and synthesis of recent empirical studies. Psychol Bull 1993;**113**(1):164-180.

37. Martin, G. Reported family dynamics, sexual abuse, and suicidal behaviors in community adolescents. Arch Suicide Res 1996;**2**:183-195.

38. Peters, D.K., & Range, L.M. Childhood sexual abuse and current suicidality in college women and men. Child Abuse Negl 1995;**19**:335-341.

39. Putman, F.W. Ten-year research update review: Child sexual abuse. J Am Acad Child Adolesc Psychiatry 2003;**42**(3):269-278

40. Stepakoff, S. Effects of sexual victimization on suicidal ideation and behaviour in US college women. Suicide and Life-Threatening Behavior 1998;**28**:107-126.

41. Malinosky-Rummel, R., & Hansen, D.J. Long-term consequences of childhood physical abuse. Psychol Bull 1993;**144**:68-79

For peer review only - http://bmjopen.bmj.com/site/about/guidelines.xhtml

For peer review only

42. Silverman, A.B., Reinherz, H., & Giaconia, R.M. The long-term sequelae of child and adolescent abuse: A longitudinal community study. Child Abuse Negl 1996;**20**:709-723

43. Chapman, D.P., Whitfield, C.L., Felitti, V.J., Dube, S.R., Edwards, V.J., & Anda, R.F. Adverse childhood experiences and the risk of depression in adulthood. J Affect Disord 2004;**82**:217-225

44. Dube, S.R., Felitti, V.J., Dong, M., Chapman, D.P., Giles, W.H., & Anda, R.F. Childhood abuse, neglect, and household dysfunction and the risk of illicit drug use: The adverse childhood experiences study. Pediatricc 2003;**111**:564-572.

45. Jewkes, R.K., Dunkle, K., Nduna, M., et al. Associations between childhood adversity and depression, substance abuse and HIV and HSV2 incident infections in rural South African youth. Child Abuse Negl 2010;**34**:833-841.

46. Seedat, S., Stein, D.J., Jackson, P.B., Heeringa, S.G., Williams, D.R., Myer, L. Life stress and mental disorders in the South African Stress and Health study. South African Medical Journal 2009a;**99:**375-382.

47. Williams, D.R., Herman, A., Kessler, R.C., et al. The South Africa Stress and Health Study: Rationale and Design. Metab Brain Dis 2004;**19**(1/2):135-147.

48. Statistics South Africa. Census 2001: Census in Brief. Pretoria: Statistics South Africa. 2001. Available from http://www.statssa.gov.za/census01/html/CInBrief/CIB2001.pdf (Accessed January 2014)

49. Kessler, R.C., Üstün, T.B. The World Mental Health (WMH) Survey Initiative Version of the World Health Organization (WHO) Composite International Diagnostic Interview (CIDI). Int J Methods Psychiatr Res 2004;**13**:61-98.

For peer review only - http://bmjopen.bmj.com/site/about/guidelines.xhtml

50. Seedat, S., Stein, D.J., Herman, A., et al. Twelve-month treatment of Psychiatric disorders in South Africa Stress and Health Study (World Mental Health Survey Initiative). Psychiatric Epidemiology 2008;**38**:211-220.

51. Seedat, S., Williams, D.R., Herman, A., et al. Mental health service use among South Africans for mood, anxiety and substance use disorders. South African Medical Journal 2009b;**99**:346-352.

52. World Health Organization. World Health Organization Manual of the international statistical classification of diseases, injuries and causes of death, ninth revision. Geneva, Switzerland, 1992.

53. American Psychiatric Association. Diagnostic and statistical manual of mental disorders (DSM-IV), 4th Edition. Washington: American Psychiatric Association Press, 1994.

54. Straus MA. Measuring Intrafamily Conflict and Violence: The Conflict Tactics (CT) Scales. Journal of Marriage and Family 1979;**41(1)**:75

55. Kessler, R.C., McLaughlin, K.A., Green, J.G. Childhood adversities and adult psychopathology in the WHO World Mental Health Surveys. Br J Psychiatry 2010;**197**:378-385.

56. Stein, D.J., Chiu, W.T., Hwang, I., et al. Cross-national analysis of the associations between traumatic events and suicidal behavior: Findings from the WHO World Mental Health Surveys. PloS ONE 2010;**5**(5):e10574.

57. Joiner Jr, T.E., Sachs-Ericsson, N.J., Wingate, L.R. Childhood physical and sexual abuse and lifetime number of suicide attempts: A persistent and theoretically important relationship. Behav Res Ther 2007;**45**:539-547.

For peer review only - http://bmjopen.bmj.com/site/about/guidelines.xhtml

58. Afifi, T.O., Boman, J., Fleisher, W., et al. The relationship between child abuse, parental divorce, and lifetime mental disorders and suicidality in a nationally representative adult sample. Child Abuse Negl 2009;**33**:139–147.

59. Bebbington, P.E., Cooper, C.C., Minot, S., et al. Suicide attempts, gender, and sexual abuse: data from the 2000 British Psychiatric Morbidity Survey. Am J Psychiatry 2009;**166**:1135-1140.

60. Molner, B, Buka, S, & Kessler, R. Child sexual abuse and subsequent psychopathology: results from the National Comorbidity Survey. American Journal Public Health 2001;**91**:753-760.

61. Borges, G., Angst, J., Nock, M.K., et al. Risk factors for the incidence and persistence of suicide related outcomes: a 10 year follow up study using the National Comorbidity Surveys. J Affect Disord 2008;**105**:25-33

62. Xing, X-Y., Tao, F-B., Wan, Y-H., et al. Family factors associated with suicide attempts among Chinese adolescent students: A national cross-sectional survey. J Adolesc Health 2010;**46**:592-599.

63. Gureje, O., Kola, L., Uwakwe, R., et al. The profile and risks of suicidal behaviours in the Nigerian Survey of Mental Health and Well Being. Psychol Med 2007;**37**:821-830.

64. Knauper, BC., CF, Schwarz, N., Bruce, ML., Keesler, RC. Improving the accuracy of major depression age of onset reports in the US National Comorbidity Survey. Int J Methods Psychiatr Res 1999;**8**(1):39-48

65. Brewin, CR., Andrews, B., Botlib, IH. Psychopathology and early experience: a reappraisal of retrospective reports. Psychol Bull 1993;**113**:82-98

66. Hardt, J., Rutter, M. Validity of adult retrospective reports of adverse childhood experiences: a review of the evidence. J Child Psychol Psychiatry 2004;**45**:260-273.

For peer review only - http://bmjopen.bmj.com/site/about/guidelines.xhtml

For peer review only

67. Dube, SR., Williamson, DF., Thompson, T., Felitti, VJ, Anda, RF. Assessing the reliability of retrospective reports of adverse childhood experiences amond adult HMO members attending a primary care clinic. Child Abuse Negl 2004;**28**(7):729-737.

68. Yancura, LA., Aldwin, CM. (2009). Stability and change in retrospective reports of childhood experiences over a 5-year period: Findings from the David Longitudinal Study. Psychol Aging 2009;**24**(3):715-721

69. Wilsnack, S.C., Wonderlich, S.A., Kristjanson, A.F., et al. (2002). Self reports of forgetting and remembering childhood sexual abuse in a nationally representative sample of US women. Child Abuse Negl 2002;**26**:139-147.

For peer review only

Figure 1: Schematic representation

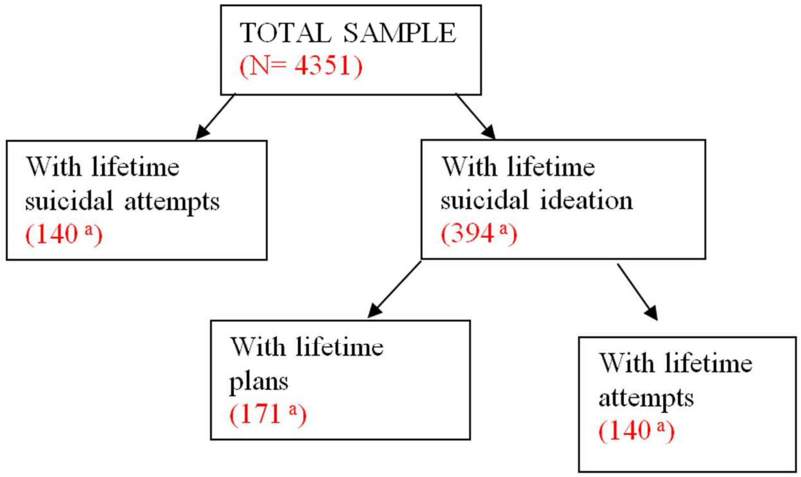

[a] Number of cases with the outcome variable; N represents the number of person years.

Schematic representation of Suicidal behaviour in the total sample
289x172mm (300 x 300 DPI)

South Africa Web Table 1. Multivariate model for associations between child adversity and LT suicidality[1]

	Life stage	Int range 4-12							
		LT Attempts in total sample[2]		Ideators among total sample[3]		Among Ideators, LT Plans[4]		Among Ideators, LT Attempts[5]	
		OR(95% CI)	Chisquare	OR(95% CI)	Chisquare	OR(95% CI)	Chisquare	OR(95% CI)	Chisquare
Physical Abuse	Childhood	0.6 (0.1-4.3)	0.3(0.60)	**3.7* (1.0-13.4)***	**4.2(0.041)***	---	---	**0.0* (0.0-0.0)***	**1177.3(<.001)***
	Teen years	**3.7* (1.5-9.2)***	**8.5(0.004)***	**3.6* (2.2-5.9)***	**26.1(<.001)***	0.3 (0.0-2.5)	1.2(0.28)	1.1 (0.3-4.7)	0.0(0.90)
	Young adult	1.6 (0.8-3.5)	1.7(0.20)	1.1 (0.7-1.8)	0.2(0.64)	0.4 (0.1-1.2)	2.7(0.10)	0.9 (0.3-3.2)	0.0(0.88)
	Later adult	**2.2* (1.0-4.8)***	**4.4(0.035)***	1.8 (0.9-3.5)	3.2(0.07)	0.4 (0.1-1.2)	2.8(0.10)	1.4 (0.4-5.3)	0.2(0.65)
Sexual Abuse	Childhood	**61.6* (4.5-841.0)***	**9.9(0.002)***	**34.8* (3.1-392.6)***	**8.6(0.003)***	---	---	---	---
	Teen years	**20.3* (2.0-210.2)***	**6.6(0.010)***	4.6 (0.3-61.6)	1.4(0.24)	---	---	---	---
	Young adult	5.1 (0.4-66.1)	1.6(0.20)	2.2 (0.3-17.5)	0.5(0.46)	---	---	**0.0* (0.0-0.0)***	**68.1(<.001)***
	Later adult	**0.0* (0.0-0.0)***	**81.7(<.001)***	**0.0* (0.0-0.0)***	**218.1(<.001)***	---	---	0.8 (0.0-16.7)	0.0(0.88)
Parent Died	Childhood	2.6 (0.1-52.0)	0.4(0.53)	1.5 (0.1-16.9)	0.1(0.76)	8.6 (0.3-234.7)	1.7(0.19)	**22.7* (1.5-338.3)***	**5.3(0.021)***
	Teen years	1.8 (0.5-6.5)	0.9(0.35)	**2.2* (1.1-4.3)***	**5.3(0.021)***	0.7 (0.1-6.8)	0.1(0.72)	0.5 (0.1-2.2)	0.8(0.38)
	Young adult	0.6 (0.2-2.4)	0.4(0.51)	1.4 (0.7-2.8)	0.7(0.40)	0.4 (0.0-2.8)	1.0(0.32)	0.6 (0.2-2.2)	0.7(0.42)
	Later adult	1.3 (0.3-4.9)	0.1(0.70)	1.6 (0.7-3.6)	1.5(0.22)	0.2 (0.0-1.6)	2.3(0.13)	0.6 (0.2-1.9)	0.8(0.37)
Parent Divorced	Childhood	3.0 (0.2-38.0)	0.7(0.39)	2.9 (0.3-24.8)	0.9(0.33)	4.6 (0.1-215.6)	0.6(0.43)	**0.0* (0.0-0.0)***	**135.3(<.001)***
	Teen years	**4.6* (1.7-12.1)***	**9.8(0.002)***	2.5 (1.0-6.1)	3.8(0.05)	0.4 (0.1-3.1)	0.8(0.37)	**4.3* (1.1-17.0)***	**4.5(0.035)***
	Young adult	1.7 (0.7-4.5)	1.3(0.25)	1.1 (0.5-2.6)	0.1(0.74)	0.8 (0.2-4.2)	0.0(0.84)	2.9 (0.7-12.7)	2.2(0.14)
	Later adult	**4.6* (1.0-21.6)***	**3.9(0.049)***	2.4 (0.9-6.2)	3.5(0.06)	0.3 (0.0-2.5)	1.2(0.28)	1.9 (0.1-31.6)	0.2(0.65)

For peer review only - http://bmjopen.bmj.com/site/about/guidelines.xhtml

Other Parent Loss	Childhood	**0.0* (0.0-0.0)***	**53.4(<.001)***	**0.0* (0.0-0.0)***	**77.1(<.001)***	---	---	---	---
	Teen years	**0.0* (0.0-0.0)***	**238.2(<.001)***	0.2 (0.0-1.5)	2.6(0.11)	---	---	**0.0* (0.0-0.0)***	**60.3(<.001)***
	Young adult	1.2 (0.2-7.6)	0.1(0.82)	**2.9* (1.2-7.4)***	**5.5(0.019)***	0.1 (0.0-1.5)	3.0(0.08)	10.5 (0.7-160.1)	3.0(0.09)
	Later adult	1.3 (0.2-9.4)	0.1(0.80)	**5.1* (2.1-12.1)***	**14.1(<.001)***	0.2 (0.0-4.0)	1.1(0.30)	0.6 (0.1-6.9)	0.2(0.70)
Family Violence	Childhood	**0.0* (0.0-0.1)***	**12.9(<.001)***	1.9 (0.3-13.1)	0.5(0.48)	---	---	---	---
	Teen years	1.9 (0.5-7.2)	1.0(0.33)	2.1 (0.6-7.6)	1.5(0.23)	2.3 (0.1-46.2)	0.3(0.59)	0.9 (0.1-5.7)	
	Young adult	0.4 (0.1-1.5)	2.0(0.16)	0.5 (0.2-1.8)	1.1(0.30)	0.3 (0.0-2.6)	1.4(0.24)	2.1 (0.2-25.7)	0.4(0.55)
	Later adult	1.0 (0.2-6.5)	0.0(0.96)	0.9 (0.2-3.4)	0.0(0.86)	**0.0* (0.0-0.9)***	**4.3(0.037)***	0.8 (0.0-25.2)	0.0(0.92)
Physical Illness	Childhood	**0.0* (0.0-0.0)***	**44.3(<.001)***	1.4 (0.2-13.2)	0.1(0.75)	---	---	---	---
	Teen years	2.9 (0.3-27.8)	0.9(0.34)	1.5 (0.4-5.4)	0.3(0.56)	**9.9* (1.8-54.0)***	**7.3(0.007)***	1.5 (0.2-11.6)	0.1(0.71)
	Young adult	0.3 (0.0-5.1)	0.8(0.36)	1.0 (0.4-2.6)	0.0(0.96)	0.2 (0.0-4.6)	1.0(0.32)	0.1 (0.0-1.4)	3.0(0.08)
	Later adult	5.5 (0.9-32.1)	3.7(0.05)	**4.3* (1.1-15.9)***	**4.8(0.028)***	**0.0* (0.0-0.9)***	**4.1(0.042)***	1.6 (0.1-20.8)	0.1(0.73)
Financial Adversity	Childhood	**0.0* (0.0-0.0)***	**64.2(<.001)***	2.0 (0.2-22.3)	0.3(0.57)	**0.0* (0.0-0.0)***	**26.8(<.001)***	**0.0* (0.0-0.2)***	**10.0(0.002)***
	Teen years	1.9 (0.2-14.5)	0.4(0.53)	0.6 (0.2-2.3)	0.6(0.45)	1.0 (0.1-19.3)	0.0(1.00)	4.0 (0.4-42.9)	
	Young adult	0.8 (0.2-3.6)	0.1(0.76)	0.5 (0.2-1.4)	1.9(0.17)	1.7 (0.3-10.9)	0.3(0.57)	1.3 (0.2-7.7)	0.1(0.78)
	Later adult	2.1 (0.3-15.5)	0.6(0.44)	2.0 (0.5-8.4)	1.0(0.31)	0.7 (0.1-4.9)	0.1(0.75)	0.8 (0.1-4.6)	0.1(0.78)
group significance test for all types	Childhood		**347.6(<.001)***		**822.4(<.001)***		**204.6(<.001)***		**1425.4(<.001)***
	Teen years		**1168.3(<.001)***		**37.5(<.001)***		**421.4(<.001)***		**1337.0(<.001)***
	Young adult		9.9(0.27)		9.6(0.30)		**1038.1(<.001)***		**97.5(<.001)***

	Later adult		**338.1(<.001)***		**525.7(<.001)***		7.9(0.34)		5.9(0.66)
significance test for difference between types	Childhood		**301.9(<.001)***		**637.3(<.001)***		**203.1(<.001)***		**1123.3(<.001)***
	Teen years		**1004.7(<.001)***		12.4(0.09)		**374.6(<.001)***		**1283.5(<.001)***
	Young adult		5.2(0.64)		10.2(0.18)		**973.6(<.001)***		**99.6(<.001)***
	Later adult		**272.0(<.001)***		**477.0(<.001)***		4.8(0.57)		5.7(0.57)
2+ adversities	Childhood	0.6 (0.0-13.1)	0.1(0.73)	0.1 (0.0-1.3)	3.2(0.07)	---	---	---	---
	Teen years	0.2 (0.0-1.4)	2.6(0.11)	**0.3* (0.1-1.0)***	**3.9(0.048)***	0.9 (0.0-32.5)	0.0(0.93)	2.5 (0.3-18.7)	
	Young adult	3.1 (0.8-12.3)	2.7(0.10)	1.3 (0.7-2.6)	0.7(0.41)	9.1 (0.5-169.9)	2.3(0.13)	3.5 (0.4-28.8)	1.4(0.24)
	Later adult	0.2 (0.0-1.6)	2.4(0.12)	0.2 (0.1-1.1)	3.6(0.06)	**44.5* (2.5-779.1)***	**7.0(0.008)***	2.1 (0.2-18.5)	0.5(0.49)

*Significant at the .05 level, two-sided test

[1]Assessed in Part 2 sample due to having part 2 controls. Controls for the model include int (1-5 intervals), and also include significant variables from demographic and parent psychopathology, details in following footnotes

[2]Models controls for int(1-5 intervals), countries, demographics (sex, age, time-varying education), interaction between int intervals(13-19,20-29,30+) and age, education. For parent psychopathology, controlling for number of parental disorders (dummies for 1, 2+ disorders).

[3]Models controls for int(1-5 intervals), countries, demographics (sex, age, time-varying education), interaction between int intervals(13-19,20-29,30+) and age, education. For parent psychopathology, controlling for number of parental disorders (dummies for 1, 2+ disorders).

[4]Models controls for int(1-5 intervals), countries, demographics (sex, age, time-varying education), interaction between int intervals(13-19,20-29,30+) and age, education. For parent psychopathology, controlling for types of parental disorders (6 dummies).

[5]Models controls for int(1-5 intervals), countries, demographics (sex, age, time-varying education), interaction between int intervals(13-19,20-29,30+) and age, education. Parent psychopathology not controlled for due to insignificance in previous models.

For peer review only - http://bmjopen.bmj.com/site/about/guidelines.xhtml

STROBE Statement—checklist of items that should be included in reports of observational studies

	Item No	Recommendation
**Title and abstract**	1	(*a*) Indicate the study's design with a commonly used term in the title or the abstract
		(*b*) Provide in the abstract an informative and balanced summary of what was done and what was found
**Introduction**		
Background/rationale	2	Explain the scientific background and rationale for the investigation being reported
Objectives	3	State specific objectives, including any prespecified hypotheses
**Methods**		
Study design	4	Present key elements of study design early in the paper
Setting	5	Describe the setting, locations, and relevant dates, including periods of recruitment, exposure, follow-up, and data collection
Participants	6	(*a*) *Cohort study*—Give the eligibility criteria, and the sources and methods of selection of participants. Describe methods of follow-up<br>*Case-control study*—Give the eligibility criteria, and the sources and methods of case ascertainment and control selection. Give the rationale for the choice of cases and controls<br>*Cross-sectional study*—Give the eligibility criteria, and the sources and methods of selection of participants
		(*b*) *Cohort study*—For matched studies, give matching criteria and number of exposed and unexposed<br>*Case-control study*—For matched studies, give matching criteria and the number of controls per case
Variables	7	Clearly define all outcomes, exposures, predictors, potential confounders, and effect modifiers. Give diagnostic criteria, if applicable
Data sources/ measurement	8*	For each variable of interest, give sources of data and details of methods of assessment (measurement). Describe comparability of assessment methods if there is more than one group
Bias	9	Describe any efforts to address potential sources of bias
Study size	10	Explain how the study size was arrived at
Quantitative variables	11	Explain how quantitative variables were handled in the analyses. If applicable, describe which groupings were chosen and why
Statistical methods	12	(*a*) Describe all statistical methods, including those used to control for confounding
		(*b*) Describe any methods used to examine subgroups and interactions
		(*c*) Explain how missing data were addressed
		(*d*) *Cohort study*—If applicable, explain how loss to follow-up was addressed<br>*Case-control study*—If applicable, explain how matching of cases and controls was addressed<br>*Cross-sectional study*—If applicable, describe analytical methods taking account of sampling strategy
		(*e*) Describe any sensitivity analyses

Continued on next page

For peer review only

**Results**		
Participants	13*	(a) Report numbers of individuals at each stage of study—eg numbers potentially eligible, examined for eligibility, confirmed eligible, included in the study, completing follow-up, and analysed
		(b) Give reasons for non-participation at each stage
		(c) Consider use of a flow diagram
Descriptive data	14*	(a) Give characteristics of study participants (eg demographic, clinical, social) and information on exposures and potential confounders
		(b) Indicate number of participants with missing data for each variable of interest
		(c) *Cohort study*—Summarise follow-up time (eg, average and total amount)
Outcome data	15*	*Cohort study*—Report numbers of outcome events or summary measures over time
		Case-control study—Report numbers in each exposure category, or summary measures of exposure
		Cross-sectional study—Report numbers of outcome events or summary measures
Main results	16	(*a*) Give unadjusted estimates and, if applicable, confounder-adjusted estimates and their precision (eg, 95% confidence interval). Make clear which confounders were adjusted for and why they were included
		(*b*) Report category boundaries when continuous variables were categorized
		(*c*) If relevant, consider translating estimates of relative risk into absolute risk for a meaningful time period
Other analyses	17	Report other analyses done—eg analyses of subgroups and interactions, and sensitivity analyses
**Discussion**		
Key results	18	Summarise key results with reference to study objectives
Limitations	19	Discuss limitations of the study, taking into account sources of potential bias or imprecision. Discuss both direction and magnitude of any potential bias
Interpretation	20	Give a cautious overall interpretation of results considering objectives, limitations, multiplicity of analyses, results from similar studies, and other relevant evidence
Generalisability	21	Discuss the generalisability (external validity) of the study results
**Other information**		
Funding	22	Give the source of funding and the role of the funders for the present study and, if applicable, for the original study on which the present article is based

*Give information separately for cases and controls in case-control studies and, if applicable, for exposed and unexposed groups in cohort and cross-sectional studies.

Note: An Explanation and Elaboration article discusses each checklist item and gives methodological background and published examples of transparent reporting. The STROBE checklist is best used in conjunction with this article (freely available on the Web sites of PLoS Medicine at http://www.plosmedicine.org/, Annals of Internal Medicine at http://www.annals.org/, and Epidemiology at http://www.epidem.com/). Information on the STROBE Initiative is available at www.strobe-statement.org.

For peer review only
